# Investigating the multi-target pharmacological mechanism of danhong injection acting on unstable angina by combined network pharmacology and molecular docking

**DOI:** 10.1186/s12906-020-2853-5

**Published:** 2020-03-02

**Authors:** Siyu Guo, Jiarui Wu, Wei Zhou, Xinkui Liu, Jingyuan Zhang, Shanshan Jia, Ziqi Meng, Shuyu Liu, Mengwei Ni, Yingying Liu

**Affiliations:** Department of Clinical Chinese Pharmacy, School of Chinese Materia Medica, Beijing University of Chinese Medicine, No. 11 of North Three-ring East Road, Chao Yang District, Beijing, China

**Keywords:** DHI, Network pharmacology, UA, Target prediction, Signaling pathway, Molecular docking

## Abstract

**Background:**

Danhong injection (DHI), which is one of the most well-known Traditional Chinese Medicine (TCM) injections, widely used to treat unstable angina (UA). However, its underlying pharmacological mechanisms need to be further clarified.

**Methods:**

In the present study, network pharmacology was adopted. Firstly, the relative compounds were obtained by a wide-scaled literatures-mining and potential targets of these compounds by target fishing were collected. Then, we built the UA target database by DisGeNET, DigSee, TTD, OMIM. Based on data, protein-protein interaction (PPI) analysis, GO and KEGG pathway enrichment analysis were performed and screen the hub targets by topology. Furthermore, evaluation of the binding potential of key targets and compounds through molecular docking.

**Results:**

The results showed that 12 ingredients of DHI and 27 putative known therapeutic targets were picked out. By systematic analysis, identified 4 hub targets (TNF, TLR4, NFKB1 and SERPINE1) mainly involved in the complex treating effects associated with coagulation and hemostasis, cell membrane region, platelet alpha granule, NF-kappa B signaling pathway and TNF signaling pathway.

**Conclusion:**

The results of this study preliminarily explained the potential targets and signaling pathways of DHI in the treatment of UA, which may help to laid a good foundation for experimental research and further clinical application.

## Background

According to American Heart Association statistics, cardiovascular and cerebrovascular diseases are the leading causes of death, imposing immense health, financial and emotional burdens on the world [1, 2]. UA, ST-segment elevation myocardial infarction (STEMI) and non–ST-segment elevation myocardial infarction (NSTEMI) have been collectively described acute coronary syndromes (ACS) [3]. However, although UA has clinical evidence of myocardial ischemia, the key characteristic is without significant myocardial injury [4–6]. Previous studies have shown that the pathogenesis of unstable angina is mainly related to platelet activation and aggregation and the inflammatory response-induced decline in the stability of atherosclerotic plaque [7, 8]. Generally, UA is usually controlled by antiplatelet medications, antithrombin, antianginal and thrombolytic therapy in clinic [3, 9]. For example, as a thiophene pyridine derivative, clopidogrel can block the activation of P2Y12 adenosine diphosphate (ADP) receptor on platelets and effectively diminish platelet aggregation [10]. Nevertheless, if patients had received clopidogrel within 5 days before undergoing coronary artery bypass grafting (CABG), the risk of major bleeding will increase [11].

TCM is a clinically proven medical practice that has been in existence for thousands of years [12–14]. DHI is a mixed extraction of *Salvia miltiorrhiza* (DS; Dan Shen; family: Lamiaceae) and *Carthami Flos* (HH; Hong Hua; family: Compositae /Asteraceae) [15–17]. DHI has been widely used as an important adjuvant for the treatment of cardiovascular and cerebrovascular diseases in China [14, 18, 19]. Even more importantly, a previous study demonstrated that DHI combined with conventional medicines could improve the electrocardiogram and reduce the symptoms of angina for the treatment of UA [20].

**Fig. 1:**
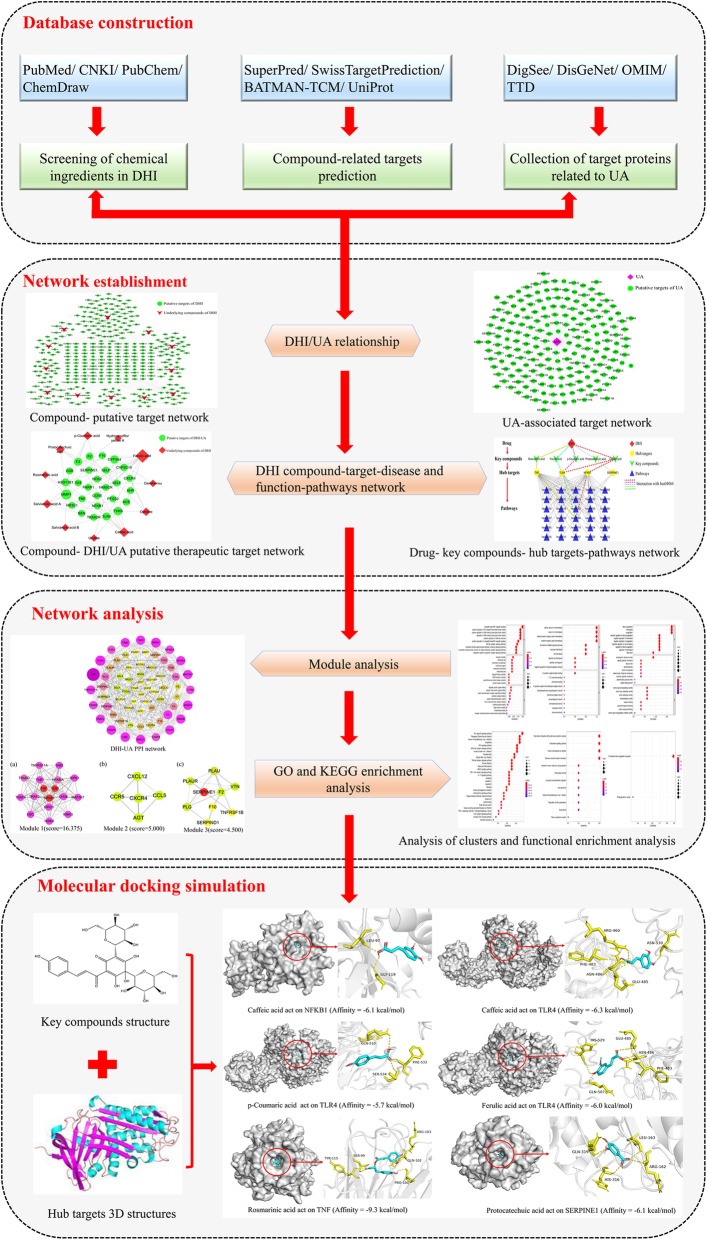
Network pharmacology and molecular docking workflow of DHI for the treatment of UA. (The software of PowerPoint was used to generate the figure)

Network pharmacology with systematic and holistic characteristics has become a promising method to explain the complex interactions between herbs and diseases at the system level [21–23]. To summarize, this study aimed to identify the potential targets and pathways of DHI as a therapy against UA using the network pharmacology approach, and systematically elucidate the mechanism of DHI in the treatment of UA. The detailed workflow was shown in Fig. 1.

## Methods

### Identification of DHI compound

To collect the chemical ingredients of DHI, PubMed (https://www.ncbi.nlm.nih.gov/pubmed/), and China National Knowledge Infrastructure Database (CNKI, http://www.cnki.net/) were applied. Furthermore, the PubChem [24] (https://pubchem.ncbi.nlm.nih.gov/), and ChemDraw [25] (http://www.chemdraw.com.cn/) was used to find Canonical simplified molecular input line entry specification (SMILES) information of the compounds.

### Screening compound targets for DHI

We searched the SuperPred [26] (http://prediction.charite.de/), SwissTargetPrediction [27] (http://www.swisstargetprediction.ch/) and BATMAN-TCM [28] (http://bionet.ncpsb.org/batman-tcm/) for potential targets related to DHI compounds. What’s more, the UniProt [29] (http://www.uniprot.org/) was utilized to convert the protein name of the DHI bioactive ingredients to the gene names. The search results were filtered to retain only studies conducted on “*Homo sapiens* (Human)” so that the names can be standardized and data duplicates deleted.

### Collection of target proteins associated with UA

Upload “Unstable angina”, “UA”, and “Unstable angina Pectori” as search terms to the DigSee (http://210.107.182.61/geneSearch/) [30], DisGeNET (http://www.disgenet.org/search) [31], OMIM (https://omim.org/) [32] and Therapeutic Target Database (TTD, https://db.idrblab.org/ttd/) [33]. In addition, the species was set to “*Homo sapiens* (Human)”. The intersection between the UA-related human gene and the target gene of the active compound was retained for further analysis.

### Protein-protein interaction (PPI) network construction

The names of putative UA/compound targets were submitted to STRING 11.0 database [34] (https://string-db.org/) as a central protein, which stores information about protein interactions. Only “*Homo sapiens*” proteins with the confidence score higher than 0.7 were picked out.

### Network establishment and module analysis

To characterize the therapeutic mechanisms of DHI against UA from a network target perspective, the Cytoscape 3.7.1 [35] (https://cytoscape.org/) were employed to construct six visualization networks as follows: (1) DHI compound-predicted target network; (2) UA-associated target network; (3) Compound- DHI/UA putative therapeutic target network; (4) DHI-UA PPI network; (5) Module analysis network; (6) Drug- key compounds- hub targets-pathways network. The “degree” is regarded as the number of edges connected to it [21, 36]. The “edges” stand for the interaction, association, or any other well-defined relationship [37]. Moreover, the “betweenness” indicates the amount of shortest paths that go through a given node [38, 39]. Besides, the “closeness” emblematizes the inverse of the sum of the distances from one node to the other [40, 41]. The higher the quantitative value of a node’s network parameters, such as degree, betweenness, and closeness, the more important the node is.

Molecular Complex Detection (MCODE) algorithms can find dense regions of interaction in PPI networks based on complex connection data [42]. In the present study, we identified the dense regions of DHI-UA PPI network according to the default parameters of MCODE (Degree Cutoff = 2; Node Score Cutoff = 0.2; K-Core = 2; Max. Depth = 100) [43]. Whereafter, the hub genes in each significant module were further analyzed.

### Functional enrichment analysis

To evaluate the role of potential core targets by bioinformatic annotation, the R 3.6.1 software with the Bioconductor package was manipulated, including Gene Ontology (GO) knowledgebase (http://geneontology.org/), Kyoto Encyclopedia of Genes and Genomes (KEGG) pathway enrichment analysis (https://www.genome.jp/kegg/) [44–46].

### Molecular docking simulation

Initially, AutoDockTools 1.5.6 was employed to set the number of rotatable bonds for 12 small molecule compounds obtained [47]. Subsequently, collecting the protein conformation is performed in the Protein Data Bank database (PDB, https://www.rcsb.org/) [48]. The screening conditions were set as follows: (1) the protein structure is obtained by X-crystal diffraction; (2) the crystal resolution of the protein is less than 3 Å; (3) preferential selection of protein structures reported in the literature of molecular docking; (4) the organism comes from *Homo sapiens*. Based on the above conditions, a total of 11 core target protein PDB IDs were gathered. At the same time, the Notepad++ (https://notepad-plus-plus.org/) and AutoDockTools were applied to not only remove water molecules and pro-ligand small molecules, but also hydrogenate and charge. Finally, molecular docking calculations were performed using Autodock Vina 1.1.2 [49]. The PyMol 2.3.2 (https://pymol.org/2/) software were wielded to visualize the docking results [50, 51].

## Results

### Compound- putative target network

**Fig. 2:**
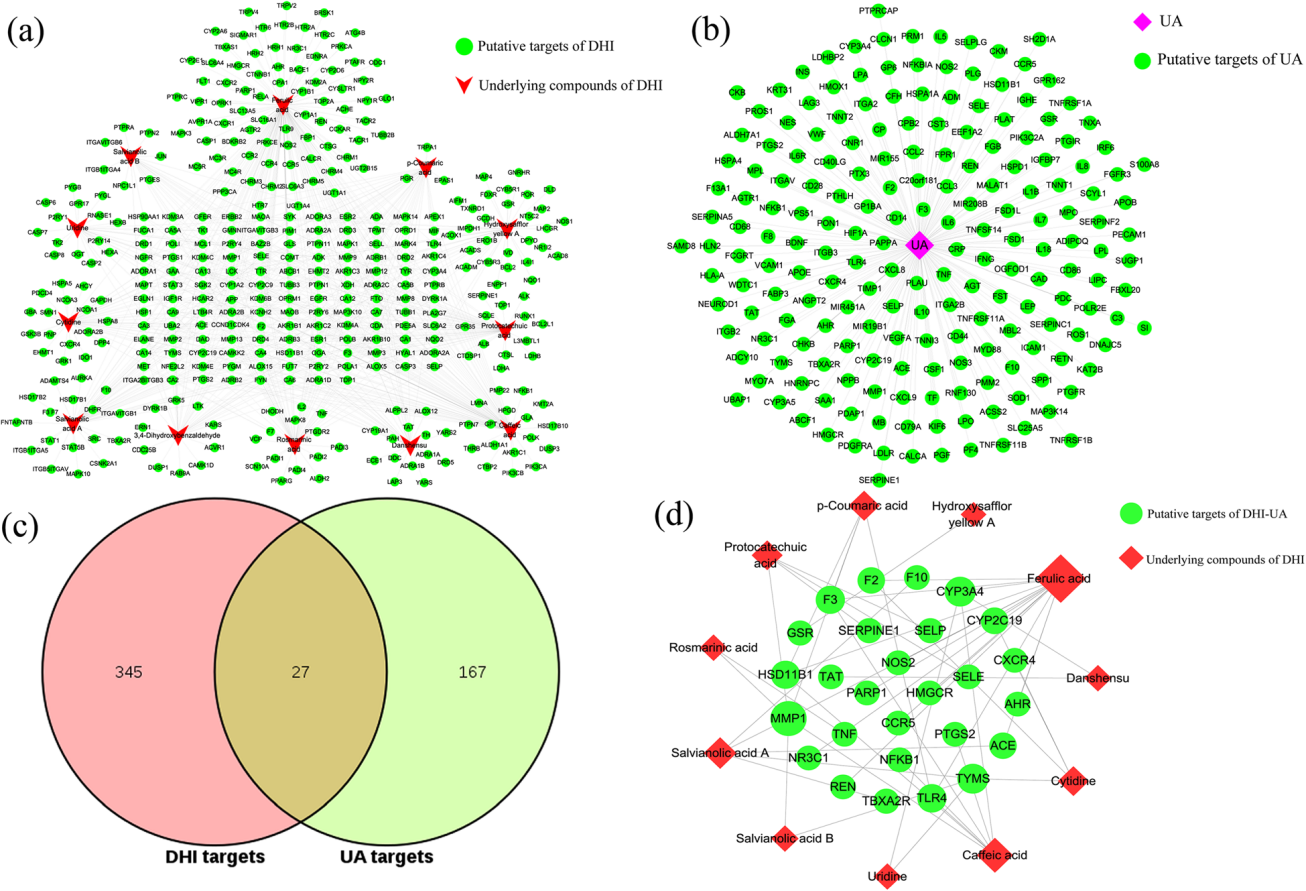
Analysis of the active compounds of DHI and putative DHI-UA targets. (**a**) Compound- predicted target network of DHI. (**b**) The UA targets’ network plotting. (**c**) Compound- DHI/UA putative therapeutic target network plotting. The size of the nodes is directly proportional to the degree of the nodes. (The software of Cytoscape 3.7.1 was used to generate the figure)

After deleting duplicate data, a total of 12 major compounds were collected as candidate compounds (Table 1) [52]. And all of the chemical constituents of DHI in Table S1. After supplementing and eliminating the targets obtained, a total of 372 compound-associated targets were identified. By analyzing the DHI compound- predicted target network, we found that the number of nodes was 384 (12 compound nodes, 372 compound-associated target nodes), and the number of edges was 708 (Fig. 2(a)). As shown in Fig. 2(a), a single target can be co-regulated by a variety of compounds to trigger the biological effects, which may play a vital role in treating UA. For example, CA12 were modulated by Rosmarinic acid, Salvianolic acid B and so on.

**Table 1:** Analysis of the 12 underlying compounds in DHI

NO.	Compound	Molecular Formula	Degree	Structure
1	Salvianolic acid B	C_36_H_30_O_16_	27	
2	Salvianolic acid A	C_26_H_21_O_10_	59	
3	Rosmarinic acid	C_18_H_15_O_8_	57	
4	Danshensu	C_9_H_9_O_5_	41	
5	Uridine	C_9_H_11_N_2_O_6_	34	
6	Hydroxysafflor yellow A	C_27_H_31_O_16_	49	
7	Caffeic acid	C_9_H_7_O_4_	95	
8	Protocatechuic acid	C_7_H_5_O_4_	53	
9	3,4-Dihydroxybenzaldehyde	C_7_H_5_O_3_	39	
10	Cytidine	C_9_H_12_N_3_O_5_	47	
11	p-Coumaric acid	C_9_H_7_O_3_	40	
12	Ferulic acid	C_10_H_9_O_4_	167

### UA targets’ network and DHI-UA PPI network

**Fig. 3:**
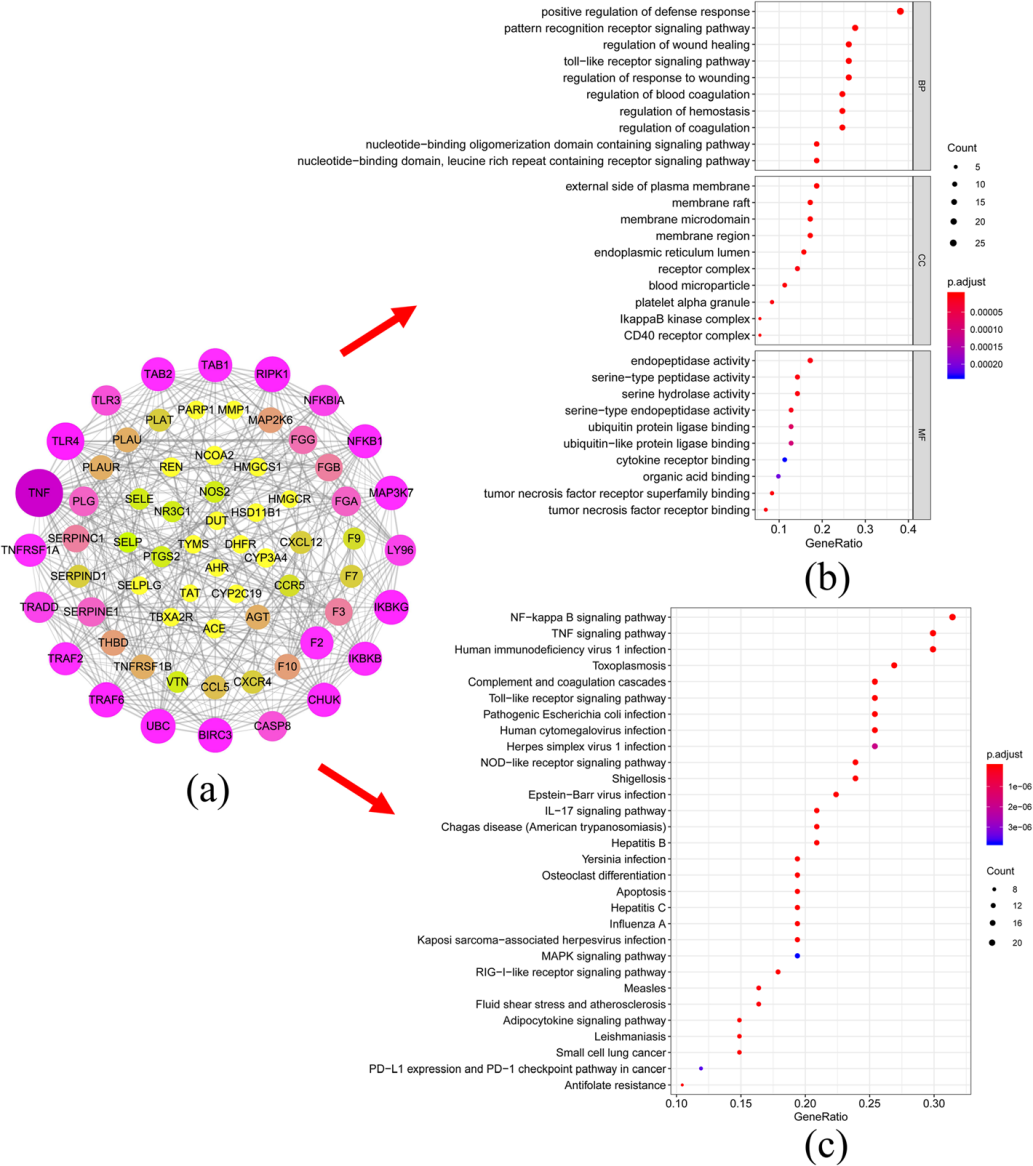
Analysis of DHI-UA PPI network and functional enrichment analysis. (**a**) DHI-UA PPI network plotting. From yellow to purple, the degree was increasing, and thicker edges denoted the stronger interactions. The size of the nodes is directly proportional to the degree of the nodes. (**b**) GO enrichment analysis of DHI-UA PPI network. *p*-value< 0.01 and *q*-value< 0.05. (**c**) KEGG pathway analysis of DHI-UA PPI network. *p*-value< 0.05 and *q*-value< 0.05. (The software of Cytoscape 3.7.1 and R 3.6.1were used to generate the figure)

There are 194 targets related to UA retrieved from DigSee, DisGeNet, OMIM and TTD database, which uploaded to the Cytoscape for network mapping (Fig. 2(b)). The UA targets’ network was constructed of 195 nodes (1 UA nodes, 194 UA target nodes) and 194 edges. Furthermore, by intersecting the two networks of Fig. 2(a) and Fig. 2(b), 27 DHI/UA putative therapeutic targets were obtained (Fig. 2(c, d)). Then, these targets brought in the STRING database to set up the PPI network (Fig. 3(a)). The network had 66 nodes, which interacted with 340 edges. From yellow to purple, the degree was increasing, and thicker edges denoted the stronger interactions. According to average degree value > 10, average betweenness > 0.024 and average closeness > 0.44, a total of 8 potential core targets were procured (Table 2). Based on topological analysis, the results together indicated that the top mutual target proteins had multiple beneficial biological functions for treating UA at the molecular level.

**Table 2:** Topological information of 8 potential core targets

UniProt ID	Targets name	Protein name	Betweenness Centrality	Closeness Centrality	Degree
P01375	TNF	Tumor necrosis factor	0.377818	0.632653	34
O00206	TLR4	Toll-like receptor 4	0.109735	0.53913	23
P19838	NFKB1	Nuclear factor NF-kappa-B p105 subunit	0.03903	0.492063	19
Q9Y6Y9	LY96	Lymphocyte antigen 96	0.037379	0.442857	16
P02671	FGA	Fibrinogen alpha chain	0.037342	0.459259	14
P05121	SERPINE1	Plasminogen activator inhibitor 1	0.058741	0.492063	14
P02679	FGG	Fibrinogen gamma chain	0.031082	0.455882	13
P13726	F3	Tissue factor	0.043337	0.484375	12

### Module analysis and functional enrichment analysis

**Fig. 4:**
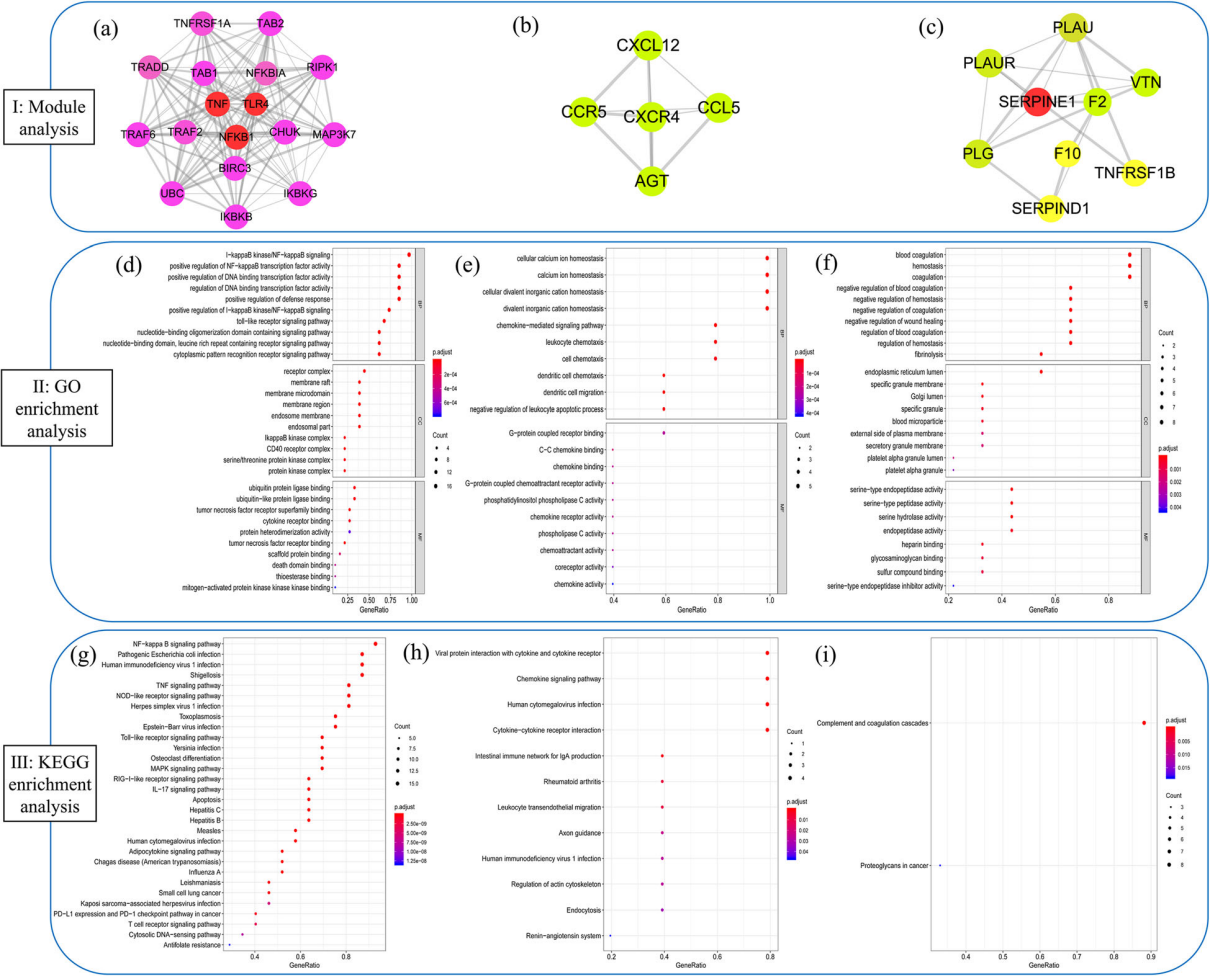
Analysis of clusters and functional enrichment analysis. I: Clusters of the DHI-UA PPI network. (**a**) Module 1 (score = 16.375). (**b**) Module 2 (score = 5.000). (**c**) Module 3(score = 4.500). Red circles represent DHI-UA targets, and the remaining circles represent other human protein targets associated with disease treatment targets. II: GO enrichment analysis for each cluster. The top 10 GO terms were shown in the figure; *p*-value< 0.01 and *q*-value< 0.05; (**d**) GO enrichment analysis of Module 1. (**e**) GO enrichment analysis of Module 2. (**f**) GO enrichment analysis of Module 3. III: KEGG pathway enrichment analysis for each cluster. *p*-value< 0.05 and *q*-value< 0.05; (**g**): Pathway analysis of Module 1. (**h**) Pathway analysis of Module 2. (**i**) Pathway analysis of Module 3. The top 30 KEGG pathways were shown in the figure. The y-axis shows significantly enriched KEGG pathways, and x-axis shows the gene counts. (The software of Cytoscape 3.7.1 and R 3.6.1 were used to generate the figure)

A network module or cluster is defined as a highly interconnected set of nodes that helps discover and reveal hidden biological information within the network [53]. In order to identify the potential mechanism of the 8 key targets, the DHI-UA PPI network was divided into 6 clusters. Ultimately, 3 modules with a score of ≥4.5were selected (Fig. 4(I)). And a total of 4 core targets (TNF, TLR4, NFKB1, SERPINE1) were clustered in these 3 modules.

Next, we performed GO enrichment analysis (*p*-value< 0.01 and *q*-value< 0.05) of the identified DHI-UA PPI network and 3 core modules to gain insights into the cellular component (CC), molecular function (MF) and biological processes (BP) that are affected in UA (Fig. 3(b) and Fig. 4(II)). The results indicated that module 1 was highly correlated with signal transduction, transcription factor activity, cell membrane region, tumor necrosis factor, and protein kinase. Module 2 was highly associated with chemokine. Module 3 was highly linked to blood coagulation, hemostasis, platelet alpha granule and serine-type peptidase activity (Table S2 and S3). Overall, the potential targets were highly connected with regulation of coagulation and hemostasis, cell membrane region, platelet alpha granule, peptidase activity and cofactor binding.

Furthermore, KEGG pathway enrichment analysis were carried out for the DHI-UA PPI network and 3 modules (*p*-value< 0.05 and *q*-value< 0.05) (Fig. 3(c) and Fig. 4(III)). The results demonstrated that module 1 was highly correlated with signal transduction, immune system, cardiovascular disease and infectious disease. Module 2 was highly associated with immune system, organismal systems, human diseases and cellular processes. Module 3 was highly linked to immune system and cancer (Table S4 and S5). In conclusion, we recognized 78 UA-related signaling pathways, NF-kappa B, TNF, complement and coagulation cascades, and toll-like receptor signaling pathway et al. Therefore, the results imply that DHI treats UA by participating in above BP, CC and MF and signaling pathway.

### Molecular docking simulation

**Fig. 5:**
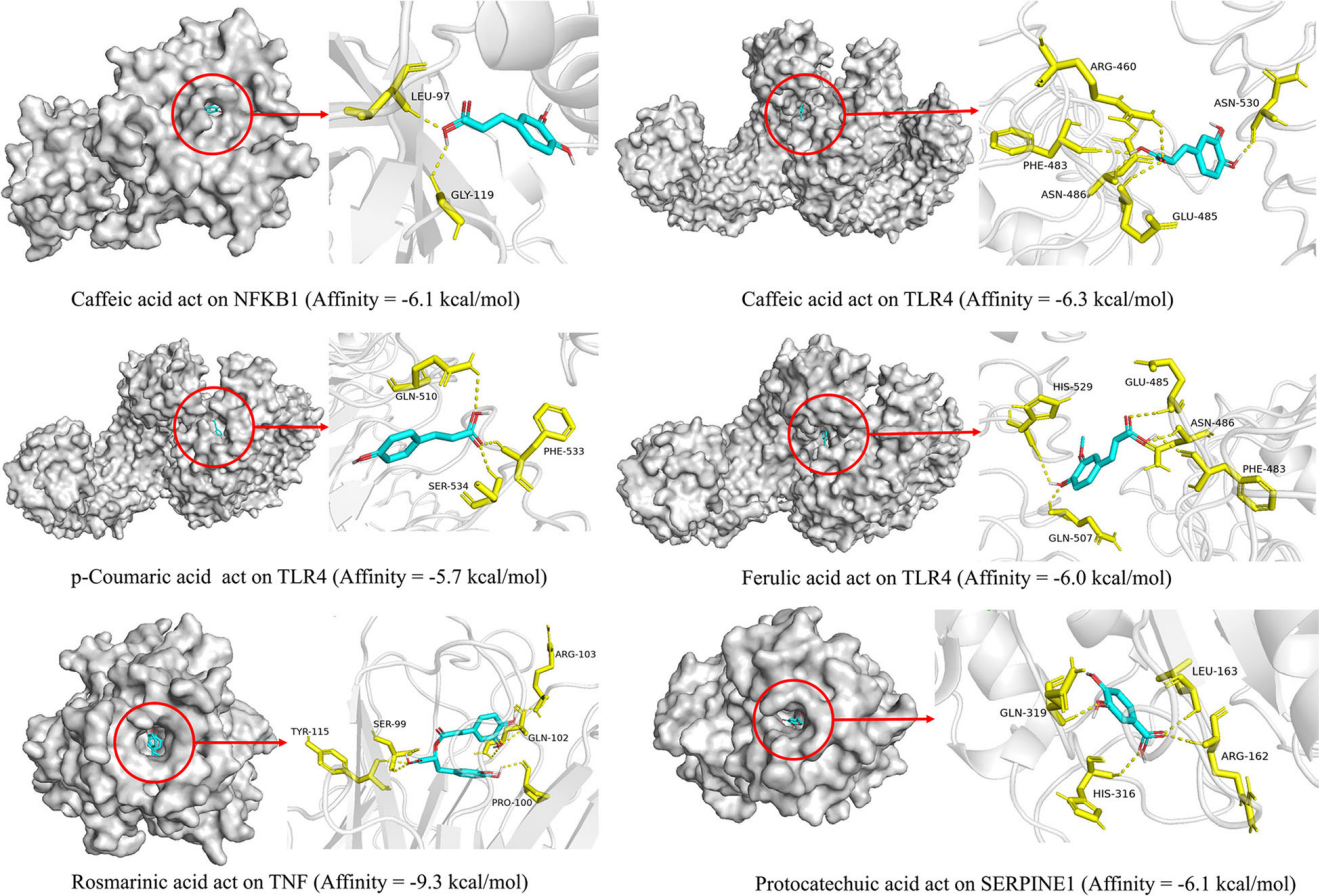
Analysis of target-compound docking simulation. (The software of PyMol 2.3.2 was used to generate the figure)

In this paper, four potential targets with five corresponding compounds were simulated by molecular docking, and the docking results were analyzed. Using Pymol software, these five compounds were observed to enter the active pocket of the protein (Fig. 5). Taking the top 2 predicted target-compound pairs in affinity (kcal/mol) as an example for analysis (Table 3). Rosmarinic acid small molecule mainly forms 9 hydrogen bonds with GLN-102, PRO-100, SER-99, TYR-115 and ARG-103 residues on TNF. Caffeic acid small molecule mainly forms 5 hydrogen bonds with ARG-460, PHE-483, ASN-486, GLU-485 and ASN-530 residues on TLR4.

**Table 3:** The molecular docking results analysis. (The software of PyMol 2.3.2 was used to generate the protein structure figures)

No.	Proteins	PDB ID	Protein structure	Test compounds	Affinity (kcal/mol)
1	NFKB1	2O61		Caffeic acid	−6.1
2	TLR4	4G8A		Caffeic acid	−6.3
				p-Coumaric acid	−5.7
				Ferulic acid	−6.0
3	TNF	1TNF		Rosmarinic acid	−9.3
4	SERPINE1	1A7C		Protocatechuic acid	−6.1

### Drug-key compounds- hub targets-pathway network construction

**Fig. 6:**
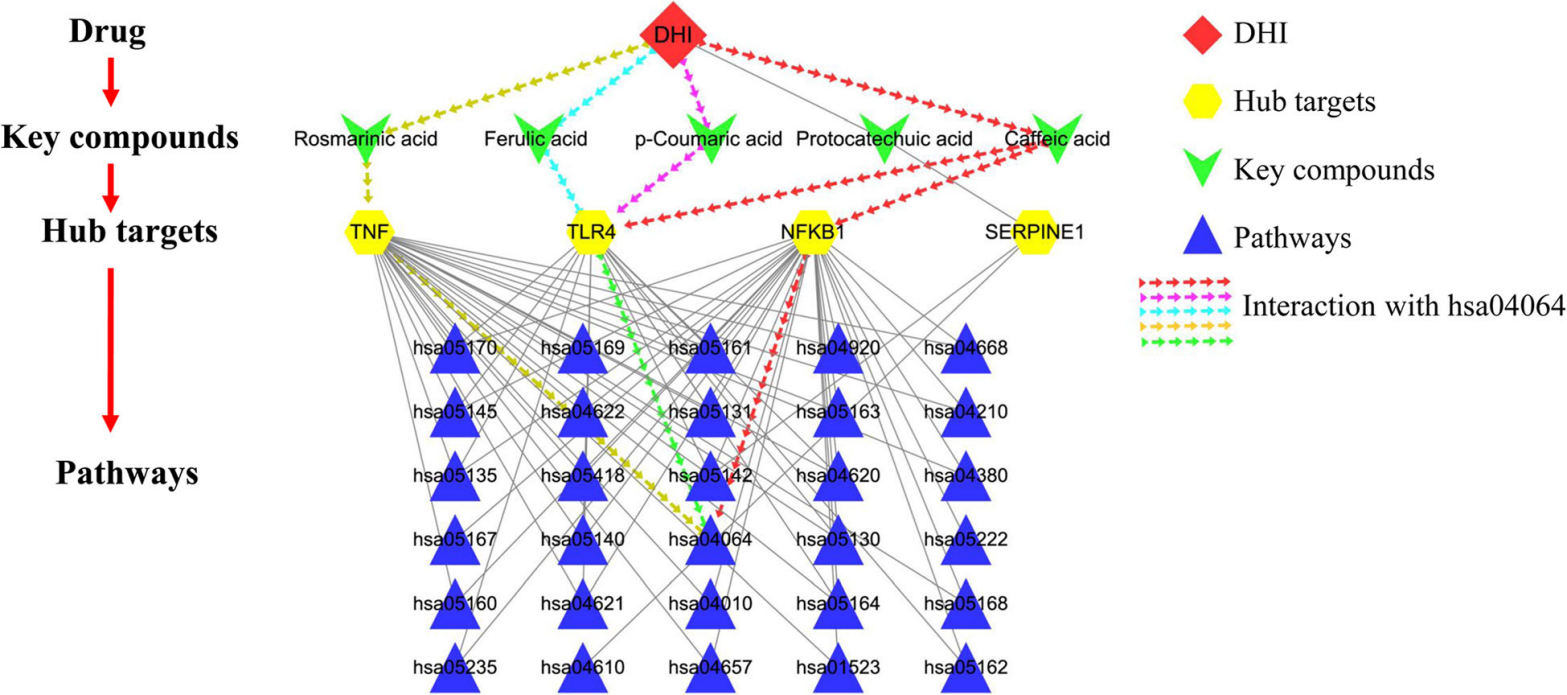
Drug- key compounds- hub targets-pathways network. (The software of Cytoscape 3.7.1 was used to generate the figure)

**Fig. 7:**
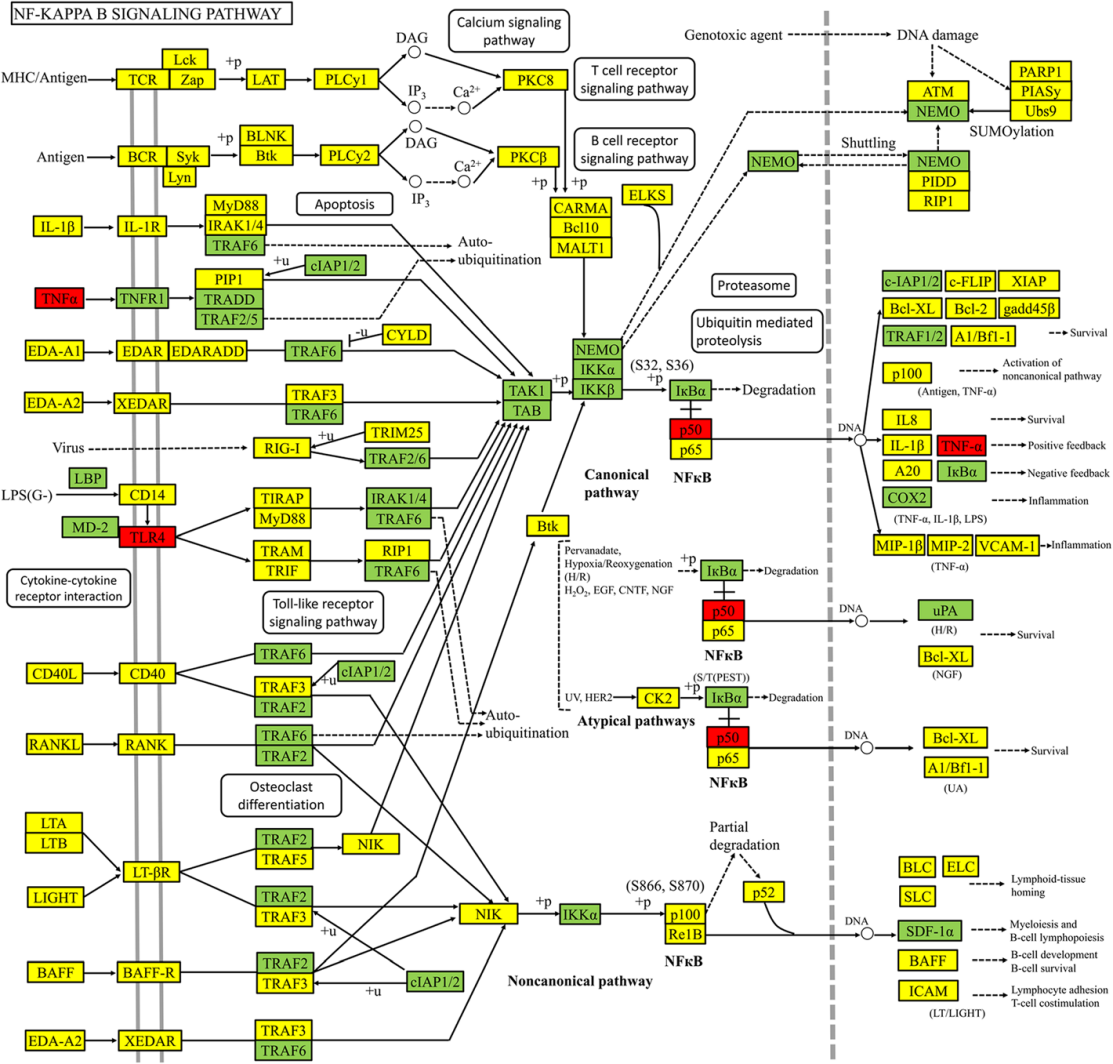
Modulating NF-kappa B signaling pathway of DHI against UA. Hub targets were colored in red, targets of DHI-UA were colored in green, and other protein targets in the pathway were colored in yellow. (The software of PowerPoint was used to generate the figure)

In order to systematically and holistically explain the mechanism of DHI in the treatment of UA, Cytoscape software was operated to construct a drug-key compounds- hub targets -pathway network (Fig. 6). As shown in Fig. 6, there were a total of 40 nodes and 81 edges. As a consequence, those pathways were tightly interacted with 4 hub targets (TNF, TLR4, NFKB1, SERPINE1). It’s worth noting that the compound with the highest degree value was caffeic acid (degree = 4). The target with the highest degree value was NFKB1 (degree = 30). The KEGG pathway with the highest degree value was chagas disease (American trypanosomiasis) (hsa05142, degree = 3). However, the NF-kappa B signaling pathway (hsa04064) with the smallest *p*-value and *q*-value will be analyzed as an important pathway (Fig. 7).

## Discussion

TCM, a complex mixed system with multiple ingredients and multiple targets, has traditionally used to prevent and treat various cardiovascular diseases (CVDs) for a long time [54–56]. Although DHI can effectively cure UA, its pharmacological mechanism of action remains unclear. Consequently, in the present study, a pharmacology network method was executed to identify bioactive compounds, potential targets and the pathways modulated by these compounds in DHI treatment of UA.

Based on module and network topology analysis, the four potential hub targets were found: TNF, TLR4, NFKB1 and SERPINE1. The studies of Biasucci et al. and Huang et al. showed that patients with UA have significantly increased proinflammatory cytokines compared to healthy individuals [57, 58]. Notably, this enhanced inflammatory activity appears to be not only an apparent phenomenon, but may also be related to the pathogenesis of UA. For example, proinflammatory cytokines such as TNF-α may enhance thrombus activity by increasing the expression of monocyte/macrophage tissue factor, as well as cause plaque instability by enhancing apoptosis and degradation of matrix metalloproteinases in atherosclerotic plaques [59–61]. More and more studies have shown that TLR4 was one of the important factors leading to the inflammatory process of atherosclerosis, intimal hyperplasia and accelerated formation of atherosclerotic plaque [62, 63]. Meanwhile, a previous study demonstrated that TLR4 were more frequently expressed in classical monocytes of UA patients than control group individuals [64]. In addition, binding of the ligand to the extracellular domain of TLR4 triggers the production of pro-inflammatory cytokines such as TNF-α and interleukin 6 (IL-6) [65]. Earlier studies from this laboratory demonstrated that nuclear factor NF-kappa-B p105 subunit (NFKB1, p50/p105), one of the five subunits of NF-κB, widely implicated in many biological processes such as immunity, inflammation, cell growth, differentiation, apoptosis and tumorigenesis [66, 67]. Importantly, Jin et al. shown that NFKB1 gene mutant is significantly connected with the severity of coronary artery in ACS patients [68]. What’s more, Lanfear et al. indicated that SERPINE1-668delG genotypes have been associated with risk of myocardial infarction [69]. Overall, based on the series of results, this study preliminarily hypothesized that DHI can treat UA by regulating proinflammatory cytokines, NFKB1 and SERPINE1.

To understand potential biological mechanism of DHI against UA, GO and KEGG functional enrichment analysis were applied. Through the KEGG pathway analysis (*p*-value< 0.05 and q-value< 0.05), we recognized 78 UA-related signaling pathways, NF-kappa B, TNF, complement and coagulation cascades, and toll-like receptor signaling pathway et al. Accordingly, these pathways may be involved in the progress of UA. Based on *p*-value and *q*-value, we choose NF-kappa B signaling pathway as most candidate signal for further study. Furthermore, nuclear factor kappa-B (NF-κB), which is a key transcription factor, may play a pivotal role in plaque instability by promoting the cascade expression of procoagulant genes and the regulation of pro-inflammatory genes in response to various stimuli [70, 71]. More importantly, NF-κB transcriptionally activates interferon, interleukins, TNF-α and adhesion molecules [72]. Activated NF-κB had been shown to reside in monocytes/macrophages, endothelial cells and smooth muscle cells in human atherosclerotic vessels, and is enhanced in coronary plaques with UA patients [73]. Notably, deficiency of NFKB1 (p50) had been demonstrated that it plays a regulatory role in NF-κB activity, leading to more inflammatory atherosclerotic lesions in low-density lipoprotein receptor (LDL-R)−/− mice [74]. C-reactive protein (CRP) and amyloid A protein are elevated in patients with UA and can predict the occurrence of subsequent unstable coronary artery events [75]. Other studies suggested that at least some of the deleterious effects of CRP in promoting plaque instability may be mediated through activation of the NF-κB signaling pathway [76]. To conclude, the results suggest that DHI may produce therapeutic effects by regulating NF-κB signaling pathway.

In this study, GO enrichment analysis was adopted to statistically analyze the modules. These potential targets (such as TNF, TLR4, NFKB1) were highly connected with regulation of coagulation, hemostasis, cell membrane region, platelet alpha granule, peptidase activity and cofactor binding. Therefore, the results suggest that DHI treats UA by participating in these BP, CC and MF.

Molecular docking analysis simulation provided a visual interpretation of the interaction between key compounds and their potential protein targets. For example, rosmarinic acid small molecule mainly forms 9 hydrogen bonds with GLN-102, PRO-100, SER-99, TYR-115 and ARG-103 residues on TNF. Rosmarinic acid, which is considered one of the most important polyphenols, has several pharmacological effects: anti-oxidant, inhibition of oxidative stress, anti-inflammatory, anti-cancer and immunomodulatory [77, 78]. Besides, it has been reported that rosmarinic acid may inhibit the expression of NF-κB promoter-related genes. Especially, TNF-α-induced NF-κB activation can be inhibited by rosmarinic acid [79]. Overall, it was speculated that the main compositions of DHI may play a significant role in the treatment of UA through hub targets in these top-ranking signaling pathways. However, some limitations of our study should be considered. For instance, the results are only based on screening already known chemical constituents of DHI, related targets, and signaling pathways from literatures and existing databases. Consequently, more in-depth researches are required for characterization of the underlying mechanisms.

## Conclusion

Via the method of network pharmacology and molecular docking, 12 ingredients of DHI and 27 putative known therapeutic targets were collected and explored the underlying mechanism of DHI in treatment of UA. Then, DHI exerted treatment effects on UA by regulating 4 hub targets: TNF, TLR4, NFKB1 and SERPINE1. Based on the results of GO and KEGG pathway enrichment analysis, we found that these hub targets ameliorated UA by participating in regulating coagulation, hemostasis, peptidase activity, signal transduction and immune system. In conclusion, the results of the study preliminarily predicted the related underlying mechanism of DHI against UA, proving that the characteristics of multi-target synergy. However, animal experiments, molecular biological experiments and clinical investigations should be performed to verify the mechanism of DHI against UA in future studies.

## Supplementary information

**Additional file 1 Supplementary 1. Table S1.** The information of all ingredients of DHI.

**Additional file 2 Supplementary 2. Table S2.** The information of GO enrichment analysis of DHI-UA PPI network.

**Additional file 3 Supplementary 3. Table S3.** The information of GO enrichment analysis for each cluster.

**Additional file 4 Supplementary 4. Table S4.** The information of KEGG pathway analysis of DHI-UA PPI network.

**Additional file 5 Supplementary 5. Table S5.** The information of KEGG pathway enrichment analysis for each cluster.

## Data Availability

All data obtained or analyzed during this study are available from published article and supplementary materials. The datasets during the current study are available from the corresponding author upon reasonable request.

## Abbreviations

ACS: Acute coronary syndromes
ADP: Adenosine diphosphate
BATMAN-TCM: Bioinformatics analysis tool for molecular mechanism of Traditional Chinese Medicine
BP: Biological processes
CA12: Carbonic anhydrase 12
CABG: Coronary artery bypass grafting
CC: Cellular component
CNKI: China National Knowledge Infrastructure Database
CRP: C-reactive protein
CVDs: Cardiovascular diseases
DHI: Danhong Injection
DigSee: Disease Gene Search Engine with Evidence Sentences
DS: Dan Shen
F3: Tissue factor
FGA: Fibrinogen alpha chain
FGG: Fibrinogen gamma chain
GO: Gene Ontology
HH: Hong Hua
IL-6: Interleukin 6
KEGG: Kyoto Encyclopedia of Genes and Genomes
LDL-R: Low-density lipoprotein receptor
LY96: Lymphocyte antigen 96
MCODE: Molecular Complex Detection
MF: Molecular function
NFKB1: Nuclear factor NF-kappa-B p105 subunit
NF-κB: Nuclear factor kappa-B
NSTEMI: Non–ST-segment elevation myocardial infarction
OMIM: Online Mendelian Inheritance in Man
PDB: Protein Data Bank
PPI: Protein-protein interaction
SERPINE1: Plasminogen activator inhibitor 1
SMILES: Simplified molecular input line entry specification
STEMI: ST-segment elevation myocardial infarction
TCM: Traditional Chinese Medicine
TLR4: Toll-like receptor 4
TNF: Tumor necrosis factor
TTD: Therapeutic Target Database
UA: Unstable angina
