# Supplementary material for: Investigating the multi-target pharmacological mechanism of danhong injection acting on unstable angina by combined network pharmacology and molecular docking
Source: BMC Complement Med Ther. 2020 Mar 2;20:66. doi: 10.1186/s12906-020-2853-5 (PMC7076845; doi:10.1186/s12906-020-2853-5)
Supplement: Supplementary file 1 — Additional file 1 Supplementary 1. Table S1. The information of all ingredients of DHI. [file 12906_2020_2853_MOESM1_ESM.docx]

Table S1. The information of all ingredients of DHI [25].

| Number | Compound |
| --- | --- |
| 1 | Danshensu |
| 2 | Protocatechuic acid |
| 3 | Chlorogenic acid |
| 4 | 3, 4-Dihydroxy benzenepropionic acid |
| 5 | Caffeic acid-O-hexoside isomer |
| 6 | *p*-Hydroxycinnamic acid isomer |
| 7 | 4-(2-Carboxyethenyl)-2-(3, 4-Dihydroxyphenyl)-2,3-dihydro- 7-hydroxy-3-methylester, [2α, 3β, 4(E)-3-benzofurancarboxylic acid |
| 8 | Coumaric acid-O-hexoside isomer |
| 9 | Protocatechuic acid isomer |
| 10 | Przewalskinic acid A/isomer |
| 11 | Caffeic acid-O-hexoside |
| 12 | Chlorogenic acid isomer |
| 13 | *p*-Hydroxycinnamic acid isomer |
| 14 | Coumaric acid-O-hexoside isomer |
| 15 | Ferulic acid isomer |
| 16 | Cryptochlorogenic acid |
| 17 | Caffeic acid |
| 18 | Salvianic acid C isomer |
| 19 | Salvianolic acid K isomer |
| 20 | Prolithospermic acid |
| 21 | Salvianic acid C |
| 22 | 4-(2-Carboxyethenyl)-2-(3,4-dihydroxyphenyl)-2,3-dihydro- 7-hydroxy-3-methylester, [2α, 3β, 4(E)-3-benzofurancarboxylic acid isomer |
| 23 | Coumaric acid-O-hexoside |
| 24 | *p*-Hydroxycinnamic acid |
| 25 | Salvianolic acid K isomer |
| 26 | Lithospermic acid isomer |
| 27 | Ferulic acid |
| 28 | Lithospermic acid isomer |
| 29 | Lithospermic acid isomer |
| 30 | Lithospermate-9-O-glucoside |
| 31 | Salviaflaside |
| 32 | 8-Hydroxy-Salvianolic acid B |
| 33 | Salvianolic acid F |
| 34 | Lithospermic acid isomer |
| 35 | 8-Hydroxy-salvianolic acid B isomer |
| 36 | Salvianolic acid D |
| 37 | Litherospermic acid monomethyl ester isomer |
| 38 | Litherospermic acid monomethyl ester |
| 39 | Salvianolic acid B isomer |
| 40 | Salvianolic acid E |
| 41 | Rosmarinic acid |
| 42 | Salvianolic acid A isomer |
| 43 | Lithospermic acid isomer |
| 44 | Salvianolic acid A isomer |
| 45 | Lithospermic acid |
| 46 | 9”-Methyl lithospermate B isomer |
| 47 | 9”-Methyl lithospermate B isomer |
| 48 | 3”'-Deoxy-salvianolic acid B isomer |
| 49 | Salvianolic acid A isomer |
| 50 | Salvianolic acid C isomer |
| 51 | Salvianolic acid B |
| 52 | Salvianolic acid L |
| 53 | Salvianolic acid A isomer |
| 54 | Lithospermic acid isomer |
| 55 | Litherospermic acid monomethyl ester isomer |
| 56 | Salvianolic acid B isomer |
| 57 | Litherospermic acid dimethyl ester |
| 58 | 9′′-Methyl lithospermate |
| 59 | Litherospermic acid dimethyl ester isomer |
| 60 | Salvianolic acid B isomer |
| 61 | Methyl rosmarinate isomer |
| 62 | 3”'-Deoxy-salvianolic acid B isomer |
| 63 | Salvianolic acid C |
| 64 | Salvianolic acid A |
| 65 | Methyl rosmarinate |
| 66 | 3”'-Deoxy-salvianolic acid B |
| 67 | 9”-Methyl lithospermate B |
| 68 | Salvianolic acid A isomer |
| 69 | Salvianolic acid A isomer |
| 70 | Litherospermic acid dimethyl ester isomer |
| 71 | Salvianolic acid C isomer |
| 72 | Salvianolic acid A isomer |
| 73 | Litherospermic acid dimethyl ester isomer |
| 74 | Litherospermic acid dimethyl ester isomer |
| 75 | Methyl salvianolic acid C |
| 76 | Paramiltioic acid isomer |
| 77 | Hydroxysafflor yellow A isomer |
| 78 | 6-Hydroxykaempferol-3,6,7-tri-O-glucoside |
| 79 | Flavonoid C-glycoside |
| 80 | Hydroxysafflor yellow A |
| 81 | Flavonoid C-glycoside |
| 82 | Iriflophenone 3-C-β-glucoside isomer |
| 83 | 6-Hydroxykaempferol-3,6-di-O-glucoside isomer |
| 84 | Hydroxysafflor yellow A isomer |
| 85 | 6-Hydroxykaempferol-3,6-di-O-glucoside |
| 86 | Eriocitrin |
| 87 | Iriflophenone 3-C-β-glucoside |
| 88 | Carthamidin-di-O-glucoside |
| 89 | Carthamidin-di-O-glucoside isomer |
| 90 | 6-Hydroxykaempferol-3-O-rutinoside-6-O-glucoside |
| 91 | 6-Hydroxykaempferol 3,6-di-O-β-D-glucoside |
| 92 | Rutin |
| 93 | Kaempferol 3-O-β-D-sophoroside |
| 94 | Carthamidin-di-O-glucoside isomer |
| 95 | Eriocitrin isomer |
| 96 | Kaempferol 3-O-Rutinoside |
| 97 | Cytidine |
| 98 | Uridine |
| 99 | Deoxyguanosine |
| 100 | Guanosine |
| 101 | Benzoic acid 4-O-β-D-glucoside |
| 102 | Phenylalanine |
| 103 | Morroniside isomer |
| 104 | Benzoic acid 4-O-β-glucoside isomer |
| 105 | Benzoic acid 4-O-β-glucoside isomer |
| 106 | Benzoic acid 4-O-β-glucoside isomer |
| 107 | Morroniside isomer |
| 108 | 3,4-Dihydroxybenzaldehyde |
| 109 | 2-Isopropylmalic acid |
| 110 | Sweroside isomer |
| 111 | 2-Methylbenzoic acid |
| 112 | Morroniside isomer |
| 113 | Sweroside |
| 114 | Morroniside |
| 115 | Cartormin |
| 116 | Neocryptotanshinone |
| 117 | Tanshinone V |
