# Supplementary material for: Investigating the multi-target pharmacological mechanism of danhong injection acting on unstable angina by combined network pharmacology and molecular docking
Source: BMC Complement Med Ther. 2020 Mar 2;20:66. doi: 10.1186/s12906-020-2853-5 (PMC7076845; doi:10.1186/s12906-020-2853-5)
Supplement: Supplementary file 2 — Additional file 2 Supplementary 2. Table S2. The information of GO enrichment analysis of DHI-UA PPI network. [file 12906_2020_2853_MOESM2_ESM.docx]

Table S2. The information of GO enrichment analysis of DHI-UA PPI network

| ONTOLOGY | ID | Description | pvalue | qvalue | Count |
| --- | --- | --- | --- | --- | --- |
| BP | GO:0030193 | regulation of blood coagulation | 3.07E-26 | 2.08E-23 | 17 |
| BP | GO:1900046 | regulation of hemostasis | 3.07E-26 | 2.08E-23 | 17 |
| BP | GO:0050818 | regulation of coagulation | 1.23E-25 | 5.56E-23 | 17 |
| BP | GO:0031349 | positive regulation of defense response | 2.86E-24 | 9.68E-22 | 26 |
| BP | GO:0061041 | regulation of wound healing | 1.08E-23 | 2.82E-21 | 18 |
| BP | GO:0002224 | toll-like receptor signaling pathway | 1.25E-23 | 2.82E-21 | 18 |
| BP | GO:0070423 | nucleotide-binding oligomerization domain containing signaling pathway | 2.64E-23 | 5.10E-21 | 13 |
| BP | GO:0035872 | nucleotide-binding domain, leucine rich repeat containing receptor signaling pathway | 3.99E-23 | 6.76E-21 | 13 |
| BP | GO:0002221 | pattern recognition receptor signaling pathway | 6.99E-23 | 1.05E-20 | 19 |
| BP | GO:1903034 | regulation of response to wounding | 3.39E-22 | 4.59E-20 | 18 |
| BP | GO:0051092 | positive regulation of NF-kappaB transcription factor activity | 1.47E-21 | 1.81E-19 | 17 |
| BP | GO:0007249 | I-kappaB kinase/NF-kappaB signaling | 1.80E-21 | 2.03E-19 | 20 |
| BP | GO:0002758 | innate immune response-activating signal transduction | 1.35E-20 | 1.40E-18 | 20 |
| BP | GO:0002753 | cytoplasmic pattern recognition receptor signaling pathway | 3.58E-20 | 3.47E-18 | 13 |
| BP | GO:0045089 | positive regulation of innate immune response | 4.54E-20 | 3.90E-18 | 21 |
| BP | GO:0002218 | activation of innate immune response | 4.60E-20 | 3.90E-18 | 20 |
| BP | GO:0032496 | response to lipopolysaccharide | 2.38E-19 | 1.90E-17 | 20 |
| BP | GO:0007596 | blood coagulation | 5.45E-19 | 4.10E-17 | 20 |
| BP | GO:0002237 | response to molecule of bacterial origin | 6.86E-19 | 4.89E-17 | 20 |
| BP | GO:0007599 | hemostasis | 7.26E-19 | 4.92E-17 | 20 |
| BP | GO:0050817 | coagulation | 7.68E-19 | 4.96E-17 | 20 |
| BP | GO:0042730 | fibrinolysis | 2.10E-18 | 1.29E-16 | 10 |
| BP | GO:0045088 | regulation of innate immune response | 2.29E-18 | 1.35E-16 | 21 |
| BP | GO:0035666 | TRIF-dependent toll-like receptor signaling pathway | 4.95E-18 | 2.80E-16 | 10 |
| BP | GO:0002756 | MyD88-independent toll-like receptor signaling pathway | 1.58E-17 | 8.57E-16 | 10 |
| BP | GO:0051091 | positive regulation of DNA binding transcription factor activity | 2.07E-17 | 1.08E-15 | 17 |
| BP | GO:0061045 | negative regulation of wound healing | 3.35E-17 | 1.64E-15 | 12 |
| BP | GO:0030195 | negative regulation of blood coagulation | 3.52E-17 | 1.64E-15 | 11 |
| BP | GO:1900047 | negative regulation of hemostasis | 3.52E-17 | 1.64E-15 | 11 |
| BP | GO:0070266 | necroptotic process | 6.16E-17 | 2.78E-15 | 10 |
| BP | GO:0050819 | negative regulation of coagulation | 1.09E-16 | 4.74E-15 | 11 |
| BP | GO:0016485 | protein processing | 1.41E-16 | 5.97E-15 | 18 |
| BP | GO:0030194 | positive regulation of blood coagulation | 2.40E-16 | 9.58E-15 | 9 |
| BP | GO:1900048 | positive regulation of hemostasis | 2.40E-16 | 9.58E-15 | 9 |
| BP | GO:0030522 | intracellular receptor signaling pathway | 2.64E-16 | 1.01E-14 | 17 |
| BP | GO:0097300 | programmed necrotic cell death | 2.68E-16 | 1.01E-14 | 10 |
| BP | GO:1903035 | negative regulation of response to wounding | 2.76E-16 | 1.01E-14 | 12 |
| BP | GO:0010803 | regulation of tumor necrosis factor-mediated signaling pathway | 3.00E-16 | 1.07E-14 | 11 |
| BP | GO:0032103 | positive regulation of response to external stimulus | 3.33E-16 | 1.14E-14 | 17 |
| BP | GO:0070555 | response to interleukin-1 | 3.35E-16 | 1.14E-14 | 15 |
| BP | GO:0050820 | positive regulation of coagulation | 3.60E-16 | 1.16E-14 | 9 |
| BP | GO:0072378 | blood coagulation, fibrin clot formation | 3.60E-16 | 1.16E-14 | 9 |
| BP | GO:0031098 | stress-activated protein kinase signaling cascade | 4.97E-16 | 1.57E-14 | 17 |
| BP | GO:0034612 | response to tumor necrosis factor | 8.19E-16 | 2.52E-14 | 17 |
| BP | GO:0050878 | regulation of body fluid levels | 8.88E-16 | 2.67E-14 | 20 |
| BP | GO:0043123 | positive regulation of I-kappaB kinase/NF-kappaB signaling | 1.34E-15 | 3.94E-14 | 14 |
| BP | GO:0071347 | cellular response to interleukin-1 | 1.72E-15 | 4.96E-14 | 14 |
| BP | GO:0051403 | stress-activated MAPK cascade | 2.08E-15 | 5.87E-14 | 16 |
| BP | GO:0051604 | protein maturation | 2.77E-15 | 7.65E-14 | 18 |
| BP | GO:0070265 | necrotic cell death | 3.66E-15 | 9.91E-14 | 10 |
| BP | GO:0090303 | positive regulation of wound healing | 5.50E-15 | 1.46E-13 | 10 |
| BP | GO:1903036 | positive regulation of response to wounding | 3.38E-14 | 8.82E-13 | 10 |
| BP | GO:0033209 | tumor necrosis factor-mediated signaling pathway | 6.36E-14 | 1.62E-12 | 13 |
| BP | GO:0043122 | regulation of I-kappaB kinase/NF-kappaB signaling | 7.58E-14 | 1.90E-12 | 14 |
| BP | GO:0070498 | interleukin-1-mediated signaling pathway | 9.10E-14 | 2.24E-12 | 11 |
| BP | GO:0071356 | cellular response to tumor necrosis factor | 1.30E-13 | 3.14E-12 | 15 |
| BP | GO:0051090 | regulation of DNA binding transcription factor activity | 1.35E-13 | 3.20E-12 | 17 |
| BP | GO:0032102 | negative regulation of response to external stimulus | 3.98E-13 | 9.30E-12 | 15 |
| BP | GO:0008625 | extrinsic apoptotic signaling pathway via death domain receptors | 5.14E-13 | 1.18E-11 | 10 |
| BP | GO:0032147 | activation of protein kinase activity | 9.01E-13 | 2.03E-11 | 15 |
| BP | GO:1902042 | negative regulation of extrinsic apoptotic signaling pathway via death domain receptors | 1.10E-12 | 2.45E-11 | 8 |
| BP | GO:0097191 | extrinsic apoptotic signaling pathway | 1.32E-12 | 2.89E-11 | 13 |
| BP | GO:0001959 | regulation of cytokine-mediated signaling pathway | 1.55E-12 | 3.34E-11 | 12 |
| BP | GO:0050727 | regulation of inflammatory response | 1.65E-12 | 3.49E-11 | 16 |
| BP | GO:0071222 | cellular response to lipopolysaccharide | 1.78E-12 | 3.71E-11 | 12 |
| BP | GO:0060759 | regulation of response to cytokine stimulus | 2.66E-12 | 5.47E-11 | 12 |
| BP | GO:0071219 | cellular response to molecule of bacterial origin | 3.04E-12 | 6.14E-11 | 12 |
| BP | GO:0000187 | activation of MAPK activity | 6.64E-12 | 1.32E-10 | 11 |
| BP | GO:0007252 | I-kappaB phosphorylation | 1.05E-11 | 2.07E-10 | 6 |
| BP | GO:0071216 | cellular response to biotic stimulus | 1.17E-11 | 2.26E-10 | 12 |
| BP | GO:0031638 | zymogen activation | 1.38E-11 | 2.63E-10 | 8 |
| BP | GO:2001234 | negative regulation of apoptotic signaling pathway | 1.94E-11 | 3.65E-10 | 12 |
| BP | GO:0052548 | regulation of endopeptidase activity | 2.50E-11 | 4.64E-10 | 15 |
| BP | GO:0002223 | stimulatory C-type lectin receptor signaling pathway | 2.83E-11 | 5.17E-10 | 10 |
| BP | GO:0002220 | innate immune response activating cell surface receptor signaling pathway | 3.60E-11 | 6.50E-10 | 10 |
| BP | GO:1902041 | regulation of extrinsic apoptotic signaling pathway via death domain receptors | 4.70E-11 | 8.37E-10 | 8 |
| BP | GO:0002755 | MyD88-dependent toll-like receptor signaling pathway | 5.64E-11 | 9.92E-10 | 7 |
| BP | GO:0052547 | regulation of peptidase activity | 6.09E-11 | 1.06E-09 | 15 |
| BP | GO:0060544 | regulation of necroptotic process | 8.05E-11 | 1.38E-09 | 6 |
| BP | GO:2001233 | regulation of apoptotic signaling pathway | 1.12E-10 | 1.90E-09 | 14 |
| BP | GO:2001237 | negative regulation of extrinsic apoptotic signaling pathway | 1.54E-10 | 2.58E-09 | 9 |
| BP | GO:0007254 | JNK cascade | 1.87E-10 | 3.09E-09 | 11 |
| BP | GO:0043406 | positive regulation of MAP kinase activity | 2.79E-10 | 4.53E-09 | 12 |
| BP | GO:2001236 | regulation of extrinsic apoptotic signaling pathway | 2.81E-10 | 4.53E-09 | 10 |
| BP | GO:0001819 | positive regulation of cytokine production | 2.84E-10 | 4.53E-09 | 14 |
| BP | GO:0043405 | regulation of MAP kinase activity | 3.76E-10 | 5.93E-09 | 13 |
| BP | GO:0031639 | plasminogen activation | 4.70E-10 | 7.32E-09 | 6 |
| BP | GO:0071902 | positive regulation of protein serine/threonine kinase activity | 5.53E-10 | 8.51E-09 | 13 |
| BP | GO:0097756 | negative regulation of blood vessel diameter | 8.58E-10 | 1.31E-08 | 8 |
| BP | GO:0038061 | NIK/NF-kappaB signaling | 9.46E-10 | 1.42E-08 | 9 |
| BP | GO:0038095 | Fc-epsilon receptor signaling pathway | 1.15E-09 | 1.72E-08 | 10 |
| BP | GO:0010939 | regulation of necrotic cell death | 1.20E-09 | 1.76E-08 | 6 |
| BP | GO:0035296 | regulation of tube diameter | 1.34E-09 | 1.93E-08 | 9 |
| BP | GO:0097746 | regulation of blood vessel diameter | 1.34E-09 | 1.93E-08 | 9 |
| BP | GO:0072376 | protein activation cascade | 1.36E-09 | 1.93E-08 | 10 |
| BP | GO:0050880 | regulation of blood vessel size | 2.27E-09 | 3.20E-08 | 9 |
| BP | GO:0035150 | regulation of tube size | 2.42E-09 | 3.36E-08 | 9 |
| BP | GO:0019229 | regulation of vasoconstriction | 2.43E-09 | 3.36E-08 | 7 |
| BP | GO:0043281 | regulation of cysteine-type endopeptidase activity involved in apoptotic process | 7.09E-09 | 9.70E-08 | 10 |
| BP | GO:0003018 | vascular process in circulatory system | 9.73E-09 | 1.32E-07 | 9 |
| BP | GO:0042310 | vasoconstriction | 1.31E-08 | 1.76E-07 | 7 |
| BP | GO:0071260 | cellular response to mechanical stimulus | 1.58E-08 | 2.10E-07 | 7 |
| BP | GO:0050900 | leukocyte migration | 1.62E-08 | 2.13E-07 | 13 |
| BP | GO:0050729 | positive regulation of inflammatory response | 1.93E-08 | 2.51E-07 | 8 |
| BP | GO:2001267 | regulation of cysteine-type endopeptidase activity involved in apoptotic signaling pathway | 2.00E-08 | 2.58E-07 | 5 |
| BP | GO:0014909 | smooth muscle cell migration | 2.24E-08 | 2.87E-07 | 7 |
| BP | GO:0038093 | Fc receptor signaling pathway | 2.45E-08 | 3.10E-07 | 10 |
| BP | GO:0050901 | leukocyte tethering or rolling | 2.52E-08 | 3.16E-07 | 5 |
| BP | GO:2000116 | regulation of cysteine-type endopeptidase activity | 2.75E-08 | 3.41E-07 | 10 |
| BP | GO:0051918 | negative regulation of fibrinolysis | 3.68E-08 | 4.53E-07 | 4 |
| BP | GO:0031663 | lipopolysaccharide-mediated signaling pathway | 4.86E-08 | 5.93E-07 | 6 |
| BP | GO:0014812 | muscle cell migration | 5.42E-08 | 6.53E-07 | 7 |
| BP | GO:0050852 | T cell receptor signaling pathway | 5.44E-08 | 6.53E-07 | 9 |
| BP | GO:0017187 | peptidyl-glutamic acid carboxylation | 5.77E-08 | 6.79E-07 | 4 |
| BP | GO:0018214 | protein carboxylation | 5.77E-08 | 6.79E-07 | 4 |
| BP | GO:0045861 | negative regulation of proteolysis | 6.83E-08 | 7.98E-07 | 11 |
| BP | GO:0009612 | response to mechanical stimulus | 7.94E-08 | 9.20E-07 | 9 |
| BP | GO:0034116 | positive regulation of heterotypic cell-cell adhesion | 8.62E-08 | 9.90E-07 | 4 |
| BP | GO:0032874 | positive regulation of stress-activated MAPK cascade | 8.98E-08 | 1.02E-06 | 8 |
| BP | GO:0070304 | positive regulation of stress-activated protein kinase signaling cascade | 9.45E-08 | 1.07E-06 | 8 |
| BP | GO:0010543 | regulation of platelet activation | 1.17E-07 | 1.31E-06 | 5 |
| BP | GO:0097296 | activation of cysteine-type endopeptidase activity involved in apoptotic signaling pathway | 1.24E-07 | 1.38E-06 | 4 |
| BP | GO:0042742 | defense response to bacterium | 1.26E-07 | 1.39E-06 | 10 |
| BP | GO:0045907 | positive regulation of vasoconstriction | 1.37E-07 | 1.50E-06 | 5 |
| BP | GO:0002718 | regulation of cytokine production involved in immune response | 1.65E-07 | 1.79E-06 | 6 |
| BP | GO:0051917 | regulation of fibrinolysis | 1.73E-07 | 1.86E-06 | 4 |
| BP | GO:0030335 | positive regulation of cell migration | 2.11E-07 | 2.25E-06 | 12 |
| BP | GO:0061756 | leukocyte adhesion to vascular endothelial cell | 2.16E-07 | 2.28E-06 | 5 |
| BP | GO:0032637 | interleukin-8 production | 2.35E-07 | 2.47E-06 | 6 |
| BP | GO:0014910 | regulation of smooth muscle cell migration | 3.29E-07 | 3.43E-06 | 6 |
| BP | GO:0010951 | negative regulation of endopeptidase activity | 3.76E-07 | 3.89E-06 | 9 |
| BP | GO:0046330 | positive regulation of JNK cascade | 3.93E-07 | 4.03E-06 | 7 |
| BP | GO:0050708 | regulation of protein secretion | 4.63E-07 | 4.71E-06 | 11 |
| BP | GO:0035690 | cellular response to drug | 4.99E-07 | 5.04E-06 | 10 |
| BP | GO:0046883 | regulation of hormone secretion | 5.22E-07 | 5.18E-06 | 9 |
| BP | GO:0007250 | activation of NF-kappaB-inducing kinase activity | 5.24E-07 | 5.18E-06 | 4 |
| BP | GO:2001269 | positive regulation of cysteine-type endopeptidase activity involved in apoptotic signaling pathway | 5.24E-07 | 5.18E-06 | 4 |
| BP | GO:1901224 | positive regulation of NIK/NF-kappaB signaling | 5.40E-07 | 5.30E-06 | 5 |
| BP | GO:0010466 | negative regulation of peptidase activity | 5.74E-07 | 5.60E-06 | 9 |
| BP | GO:0007597 | blood coagulation, intrinsic pathway | 6.62E-07 | 6.41E-06 | 4 |
| BP | GO:0032757 | positive regulation of interleukin-8 production | 6.81E-07 | 6.55E-06 | 5 |
| BP | GO:0002367 | cytokine production involved in immune response | 7.51E-07 | 7.17E-06 | 6 |
| BP | GO:0048661 | positive regulation of smooth muscle cell proliferation | 8.05E-07 | 7.63E-06 | 6 |
| BP | GO:0002791 | regulation of peptide secretion | 8.85E-07 | 8.33E-06 | 11 |
| BP | GO:0051346 | negative regulation of hydrolase activity | 9.04E-07 | 8.45E-06 | 11 |
| BP | GO:0050851 | antigen receptor-mediated signaling pathway | 9.95E-07 | 9.23E-06 | 9 |
| BP | GO:0034114 | regulation of heterotypic cell-cell adhesion | 1.02E-06 | 9.31E-06 | 4 |
| BP | GO:0071677 | positive regulation of mononuclear cell migration | 1.02E-06 | 9.31E-06 | 4 |
| BP | GO:0006919 | activation of cysteine-type endopeptidase activity involved in apoptotic process | 1.13E-06 | 1.02E-05 | 6 |
| BP | GO:2000379 | positive regulation of reactive oxygen species metabolic process | 1.20E-06 | 1.08E-05 | 6 |
| BP | GO:0045123 | cellular extravasation | 1.29E-06 | 1.15E-05 | 5 |
| BP | GO:0016579 | protein deubiquitination | 1.29E-06 | 1.15E-05 | 9 |
| BP | GO:0032872 | regulation of stress-activated MAPK cascade | 1.38E-06 | 1.22E-05 | 8 |
| BP | GO:0070302 | regulation of stress-activated protein kinase signaling cascade | 1.43E-06 | 1.26E-05 | 8 |
| BP | GO:0046879 | hormone secretion | 1.95E-06 | 1.70E-05 | 9 |
| BP | GO:0070646 | protein modification by small protein removal | 1.95E-06 | 1.70E-05 | 9 |
| BP | GO:0030168 | platelet activation | 2.01E-06 | 1.74E-05 | 7 |
| BP | GO:0006465 | signal peptide processing | 2.12E-06 | 1.82E-05 | 4 |
| BP | GO:0009615 | response to virus | 2.17E-06 | 1.85E-05 | 9 |
| BP | GO:0072577 | endothelial cell apoptotic process | 2.26E-06 | 1.91E-05 | 5 |
| BP | GO:0009914 | hormone transport | 2.68E-06 | 2.26E-05 | 9 |
| BP | GO:1901222 | regulation of NIK/NF-kappaB signaling | 2.92E-06 | 2.44E-05 | 5 |
| BP | GO:0046328 | regulation of JNK cascade | 3.15E-06 | 2.62E-05 | 7 |
| BP | GO:2000377 | regulation of reactive oxygen species metabolic process | 3.68E-06 | 3.04E-05 | 7 |
| BP | GO:0032677 | regulation of interleukin-8 production | 3.72E-06 | 3.06E-05 | 5 |
| BP | GO:0002761 | regulation of myeloid leukocyte differentiation | 4.21E-06 | 3.44E-05 | 6 |
| BP | GO:0002700 | regulation of production of molecular mediator of immune response | 4.43E-06 | 3.59E-05 | 6 |
| BP | GO:0030198 | extracellular matrix organization | 4.73E-06 | 3.82E-05 | 9 |
| BP | GO:0072593 | reactive oxygen species metabolic process | 4.87E-06 | 3.90E-05 | 8 |
| BP | GO:2000352 | negative regulation of endothelial cell apoptotic process | 5.20E-06 | 4.14E-05 | 4 |
| BP | GO:0006809 | nitric oxide biosynthetic process | 5.85E-06 | 4.63E-05 | 5 |
| BP | GO:0002429 | immune response-activating cell surface receptor signaling pathway | 6.17E-06 | 4.86E-05 | 10 |
| BP | GO:0043507 | positive regulation of JUN kinase activity | 6.28E-06 | 4.89E-05 | 5 |
| BP | GO:1903524 | positive regulation of blood circulation | 6.28E-06 | 4.89E-05 | 5 |
| BP | GO:0002687 | positive regulation of leukocyte migration | 6.54E-06 | 5.06E-05 | 6 |
| BP | GO:0002573 | myeloid leukocyte differentiation | 7.06E-06 | 5.38E-05 | 7 |
| BP | GO:0010810 | regulation of cell-substrate adhesion | 7.06E-06 | 5.38E-05 | 7 |
| BP | GO:0097305 | response to alcohol | 7.06E-06 | 5.38E-05 | 7 |
| BP | GO:0018200 | peptidyl-glutamic acid modification | 7.59E-06 | 5.75E-05 | 4 |
| BP | GO:0046209 | nitric oxide metabolic process | 7.73E-06 | 5.82E-05 | 5 |
| BP | GO:0042368 | vitamin D biosynthetic process | 8.06E-06 | 6.03E-05 | 3 |
| BP | GO:0002576 | platelet degranulation | 8.22E-06 | 6.12E-05 | 6 |
| BP | GO:0019216 | regulation of lipid metabolic process | 8.38E-06 | 6.20E-05 | 9 |
| BP | GO:0043280 | positive regulation of cysteine-type endopeptidase activity involved in apoptotic process | 8.60E-06 | 6.33E-05 | 6 |
| BP | GO:2001057 | reactive nitrogen species metabolic process | 9.42E-06 | 6.90E-05 | 5 |
| BP | GO:0002720 | positive regulation of cytokine production involved in immune response | 9.59E-06 | 6.99E-05 | 4 |
| BP | GO:0031100 | animal organ regeneration | 1.01E-05 | 7.28E-05 | 5 |
| BP | GO:0090276 | regulation of peptide hormone secretion | 1.05E-05 | 7.52E-05 | 7 |
| BP | GO:0010876 | lipid localization | 1.06E-05 | 7.52E-05 | 9 |
| BP | GO:0002002 | regulation of angiotensin levels in blood | 1.07E-05 | 7.52E-05 | 3 |
| BP | GO:0002003 | angiotensin maturation | 1.07E-05 | 7.52E-05 | 3 |
| BP | GO:1901550 | regulation of endothelial cell development | 1.07E-05 | 7.52E-05 | 3 |
| BP | GO:1903140 | regulation of establishment of endothelial barrier | 1.07E-05 | 7.52E-05 | 3 |
| BP | GO:0002768 | immune response-regulating cell surface receptor signaling pathway | 1.12E-05 | 7.85E-05 | 10 |
| BP | GO:0007584 | response to nutrient | 1.19E-05 | 8.26E-05 | 7 |
| BP | GO:0071675 | regulation of mononuclear cell migration | 1.20E-05 | 8.26E-05 | 4 |
| BP | GO:0097193 | intrinsic apoptotic signaling pathway | 1.21E-05 | 8.33E-05 | 8 |
| BP | GO:1905952 | regulation of lipid localization | 1.26E-05 | 8.66E-05 | 6 |
| BP | GO:0090066 | regulation of anatomical structure size | 1.32E-05 | 8.99E-05 | 10 |
| BP | GO:0051047 | positive regulation of secretion | 1.34E-05 | 9.05E-05 | 9 |
| BP | GO:0046165 | alcohol biosynthetic process | 1.37E-05 | 9.26E-05 | 6 |
| BP | GO:0050829 | defense response to Gram-negative bacterium | 1.54E-05 | 0.000103 | 5 |
| BP | GO:0043062 | extracellular structure organization | 1.54E-05 | 0.000103 | 9 |
| BP | GO:0048660 | regulation of smooth muscle cell proliferation | 1.55E-05 | 0.000103 | 6 |
| BP | GO:2001056 | positive regulation of cysteine-type endopeptidase activity | 1.61E-05 | 0.000106 | 6 |
| BP | GO:1904036 | negative regulation of epithelial cell apoptotic process | 1.63E-05 | 0.000106 | 4 |
| BP | GO:0043506 | regulation of JUN kinase activity | 1.63E-05 | 0.000106 | 5 |
| BP | GO:1900407 | regulation of cellular response to oxidative stress | 1.63E-05 | 0.000106 | 5 |
| BP | GO:0042445 | hormone metabolic process | 1.72E-05 | 0.000111 | 7 |
| BP | GO:0048659 | smooth muscle cell proliferation | 1.74E-05 | 0.000112 | 6 |
| BP | GO:0002726 | positive regulation of T cell cytokine production | 1.76E-05 | 0.000113 | 3 |
| BP | GO:0042362 | fat-soluble vitamin biosynthetic process | 1.76E-05 | 0.000113 | 3 |
| BP | GO:0045429 | positive regulation of nitric oxide biosynthetic process | 1.79E-05 | 0.000113 | 4 |
| BP | GO:1904407 | positive regulation of nitric oxide metabolic process | 1.79E-05 | 0.000113 | 4 |
| BP | GO:0002697 | regulation of immune effector process | 1.88E-05 | 0.000118 | 9 |
| BP | GO:0071214 | cellular response to abiotic stimulus | 1.96E-05 | 0.000122 | 8 |
| BP | GO:0104004 | cellular response to environmental stimulus | 1.96E-05 | 0.000122 | 8 |
| BP | GO:0008202 | steroid metabolic process | 2.01E-05 | 0.000125 | 8 |
| BP | GO:0035821 | modification of morphology or physiology of other organism | 2.27E-05 | 0.000141 | 6 |
| BP | GO:0044764 | multi-organism cellular process | 2.37E-05 | 0.000146 | 4 |
| BP | GO:1902882 | regulation of response to oxidative stress | 2.66E-05 | 0.000163 | 5 |
| BP | GO:0042035 | regulation of cytokine biosynthetic process | 2.80E-05 | 0.000171 | 5 |
| BP | GO:0031960 | response to corticosteroid | 2.93E-05 | 0.000177 | 6 |
| BP | GO:0070371 | ERK1 and ERK2 cascade | 2.93E-05 | 0.000177 | 8 |
| BP | GO:0070374 | positive regulation of ERK1 and ERK2 cascade | 3.01E-05 | 0.000182 | 7 |
| BP | GO:0042136 | neurotransmitter biosynthetic process | 3.25E-05 | 0.000194 | 5 |
| BP | GO:0051023 | regulation of immunoglobulin secretion | 3.27E-05 | 0.000194 | 3 |
| BP | GO:0090026 | positive regulation of monocyte chemotaxis | 3.27E-05 | 0.000194 | 3 |
| BP | GO:0023061 | signal release | 3.29E-05 | 0.000195 | 9 |
| BP | GO:0010038 | response to metal ion | 3.34E-05 | 0.000196 | 8 |
| BP | GO:0006979 | response to oxidative stress | 3.35E-05 | 0.000196 | 9 |
| BP | GO:0030072 | peptide hormone secretion | 3.35E-05 | 0.000196 | 7 |
| BP | GO:1904019 | epithelial cell apoptotic process | 3.42E-05 | 0.000199 | 5 |
| BP | GO:0045637 | regulation of myeloid cell differentiation | 3.44E-05 | 0.000199 | 7 |
| BP | GO:0032655 | regulation of interleukin-12 production | 3.61E-05 | 0.000206 | 4 |
| BP | GO:0034113 | heterotypic cell-cell adhesion | 3.61E-05 | 0.000206 | 4 |
| BP | GO:2000351 | regulation of endothelial cell apoptotic process | 3.61E-05 | 0.000206 | 4 |
| BP | GO:0046890 | regulation of lipid biosynthetic process | 3.72E-05 | 0.000212 | 6 |
| BP | GO:0002703 | regulation of leukocyte mediated immunity | 3.85E-05 | 0.000218 | 6 |
| BP | GO:0002763 | positive regulation of myeloid leukocyte differentiation | 3.90E-05 | 0.000218 | 4 |
| BP | GO:0001991 | regulation of systemic arterial blood pressure by circulatory renin-angiotensin | 3.91E-05 | 0.000218 | 3 |
| BP | GO:0010544 | negative regulation of platelet activation | 3.91E-05 | 0.000218 | 3 |
| BP | GO:1900409 | positive regulation of cellular response to oxidative stress | 3.91E-05 | 0.000218 | 3 |
| BP | GO:0019932 | second-messenger-mediated signaling | 3.95E-05 | 0.000219 | 8 |
| BP | GO:0006869 | lipid transport | 4.03E-05 | 0.000223 | 8 |
| BP | GO:0032615 | interleukin-12 production | 4.22E-05 | 0.000232 | 4 |
| BP | GO:0010950 | positive regulation of endopeptidase activity | 4.25E-05 | 0.000233 | 6 |
| BP | GO:1903706 | regulation of hemopoiesis | 4.28E-05 | 0.000233 | 9 |
| BP | GO:0001666 | response to hypoxia | 4.29E-05 | 0.000233 | 8 |
| BP | GO:1903409 | reactive oxygen species biosynthetic process | 4.33E-05 | 0.000235 | 5 |
| BP | GO:0042089 | cytokine biosynthetic process | 4.53E-05 | 0.000244 | 5 |
| BP | GO:1903428 | positive regulation of reactive oxygen species biosynthetic process | 4.55E-05 | 0.000244 | 4 |
| BP | GO:0034138 | toll-like receptor 3 signaling pathway | 4.63E-05 | 0.000248 | 3 |
| BP | GO:0042107 | cytokine metabolic process | 4.74E-05 | 0.000253 | 5 |
| BP | GO:0051817 | modification of morphology or physiology of other organism involved in symbiotic interaction | 4.96E-05 | 0.000263 | 5 |
| BP | GO:0002685 | regulation of leukocyte migration | 4.99E-05 | 0.000263 | 6 |
| BP | GO:2001235 | positive regulation of apoptotic signaling pathway | 4.99E-05 | 0.000263 | 6 |
| BP | GO:0036293 | response to decreased oxygen levels | 5.04E-05 | 0.000264 | 8 |
| BP | GO:0008217 | regulation of blood pressure | 5.15E-05 | 0.000269 | 6 |
| BP | GO:0019218 | regulation of steroid metabolic process | 5.19E-05 | 0.00027 | 5 |
| BP | GO:0032368 | regulation of lipid transport | 5.42E-05 | 0.000278 | 5 |
| BP | GO:0032727 | positive regulation of interferon-alpha production | 5.44E-05 | 0.000278 | 3 |
| BP | GO:0048305 | immunoglobulin secretion | 5.44E-05 | 0.000278 | 3 |
| BP | GO:1901889 | negative regulation of cell junction assembly | 5.44E-05 | 0.000278 | 3 |
| BP | GO:1902884 | positive regulation of response to oxidative stress | 5.44E-05 | 0.000278 | 3 |
| BP | GO:0033627 | cell adhesion mediated by integrin | 5.65E-05 | 0.000288 | 4 |
| BP | GO:1903532 | positive regulation of secretion by cell | 5.78E-05 | 0.000293 | 8 |
| BP | GO:0002532 | production of molecular mediator involved in inflammatory response | 6.06E-05 | 0.000305 | 4 |
| BP | GO:0042108 | positive regulation of cytokine biosynthetic process | 6.06E-05 | 0.000305 | 4 |
| BP | GO:1901654 | response to ketone | 6.20E-05 | 0.000311 | 6 |
| BP | GO:0045428 | regulation of nitric oxide biosynthetic process | 6.50E-05 | 0.000325 | 4 |
| BP | GO:0006694 | steroid biosynthetic process | 6.79E-05 | 0.000337 | 6 |
| BP | GO:0031099 | regeneration | 6.79E-05 | 0.000337 | 6 |
| BP | GO:0050918 | positive chemotaxis | 6.95E-05 | 0.000344 | 4 |
| BP | GO:0010952 | positive regulation of peptidase activity | 7.20E-05 | 0.000355 | 6 |
| BP | GO:0070723 | response to cholesterol | 7.31E-05 | 0.000357 | 3 |
| BP | GO:0090025 | regulation of monocyte chemotaxis | 7.31E-05 | 0.000357 | 3 |
| BP | GO:0034121 | regulation of toll-like receptor signaling pathway | 7.42E-05 | 0.000362 | 4 |
| BP | GO:0048511 | rhythmic process | 7.50E-05 | 0.000364 | 7 |
| BP | GO:0070482 | response to oxygen levels | 7.97E-05 | 0.000386 | 8 |
| BP | GO:0002407 | dendritic cell chemotaxis | 8.38E-05 | 0.0004 | 3 |
| BP | GO:0002724 | regulation of T cell cytokine production | 8.38E-05 | 0.0004 | 3 |
| BP | GO:0009110 | vitamin biosynthetic process | 8.38E-05 | 0.0004 | 3 |
| BP | GO:0042359 | vitamin D metabolic process | 8.38E-05 | 0.0004 | 3 |
| BP | GO:0032635 | interleukin-6 production | 8.57E-05 | 0.000407 | 5 |
| BP | GO:0007623 | circadian rhythm | 8.81E-05 | 0.000417 | 6 |
| BP | GO:0034599 | cellular response to oxidative stress | 9.37E-05 | 0.000442 | 7 |
| BP | GO:0022407 | regulation of cell-cell adhesion | 9.56E-05 | 0.000448 | 8 |
| BP | GO:1902930 | regulation of alcohol biosynthetic process | 9.57E-05 | 0.000448 | 4 |
| BP | GO:0032479 | regulation of type I interferon production | 9.63E-05 | 0.00045 | 5 |
| BP | GO:0032606 | type I interferon production | 0.0001 | 0.000464 | 5 |
| BP | GO:0045834 | positive regulation of lipid metabolic process | 0.0001 | 0.000464 | 5 |
| BP | GO:0033619 | membrane protein proteolysis | 0.000102 | 0.00047 | 4 |
| BP | GO:1903522 | regulation of blood circulation | 0.000102 | 0.000471 | 7 |
| BP | GO:0045785 | positive regulation of cell adhesion | 0.000103 | 0.000471 | 8 |
| BP | GO:0030099 | myeloid cell differentiation | 0.000106 | 0.000486 | 8 |
| BP | GO:0030595 | leukocyte chemotaxis | 0.000107 | 0.000486 | 6 |
| BP | GO:0050920 | regulation of chemotaxis | 0.000107 | 0.000486 | 6 |
| BP | GO:0006801 | superoxide metabolic process | 0.000108 | 0.000486 | 4 |
| BP | GO:0045670 | regulation of osteoclast differentiation | 0.000108 | 0.000486 | 4 |
| BP | GO:0003081 | regulation of systemic arterial blood pressure by renin-angiotensin | 0.000108 | 0.000486 | 3 |
| BP | GO:0050927 | positive regulation of positive chemotaxis | 0.000108 | 0.000486 | 3 |
| BP | GO:0032760 | positive regulation of tumor necrosis factor production | 0.000114 | 0.000512 | 4 |
| BP | GO:0032647 | regulation of interferon-alpha production | 0.000122 | 0.000539 | 3 |
| BP | GO:0036314 | response to sterol | 0.000122 | 0.000539 | 3 |
| BP | GO:0050926 | regulation of positive chemotaxis | 0.000122 | 0.000539 | 3 |
| BP | GO:1990776 | response to angiotensin | 0.000122 | 0.000539 | 3 |
| BP | GO:0050921 | positive regulation of chemotaxis | 0.000125 | 0.00055 | 5 |
| BP | GO:0014068 | positive regulation of phosphatidylinositol 3-kinase signaling | 0.000128 | 0.000563 | 4 |
| BP | GO:0007160 | cell-matrix adhesion | 0.000129 | 0.000563 | 6 |
| BP | GO:0046683 | response to organophosphorus | 0.00013 | 0.000565 | 5 |
| BP | GO:1903557 | positive regulation of tumor necrosis factor superfamily cytokine production | 0.000136 | 0.000585 | 4 |
| BP | GO:0033002 | muscle cell proliferation | 0.000136 | 0.000585 | 6 |
| BP | GO:0051701 | interaction with host | 0.000136 | 0.000585 | 6 |
| BP | GO:0032607 | interferon-alpha production | 0.000137 | 0.000585 | 3 |
| BP | GO:0036336 | dendritic cell migration | 0.000137 | 0.000585 | 3 |
| BP | GO:2001025 | positive regulation of response to drug | 0.000137 | 0.000585 | 3 |
| BP | GO:0071496 | cellular response to external stimulus | 0.000145 | 0.000619 | 7 |
| BP | GO:0002526 | acute inflammatory response | 0.00015 | 0.000639 | 6 |
| BP | GO:0070372 | regulation of ERK1 and ERK2 cascade | 0.000157 | 0.000667 | 7 |
| BP | GO:0032481 | positive regulation of type I interferon production | 0.00016 | 0.000673 | 4 |
| BP | GO:0001889 | liver development | 0.00016 | 0.000673 | 5 |
| BP | GO:0002702 | positive regulation of production of molecular mediator of immune response | 0.000168 | 0.000702 | 4 |
| BP | GO:0032755 | positive regulation of interleukin-6 production | 0.000168 | 0.000702 | 4 |
| BP | GO:0034109 | homotypic cell-cell adhesion | 0.000168 | 0.000702 | 4 |
| BP | GO:0010743 | regulation of macrophage derived foam cell differentiation | 0.00017 | 0.000705 | 3 |
| BP | GO:0032743 | positive regulation of interleukin-2 production | 0.00017 | 0.000705 | 3 |
| BP | GO:0042133 | neurotransmitter metabolic process | 0.000177 | 0.000729 | 5 |
| BP | GO:0061008 | hepaticobiliary system development | 0.000177 | 0.000729 | 5 |
| BP | GO:0002440 | production of molecular mediator of immune response | 0.000183 | 0.000753 | 6 |
| BP | GO:0031589 | cell-substrate adhesion | 0.000184 | 0.000753 | 7 |
| BP | GO:1904035 | regulation of epithelial cell apoptotic process | 0.000187 | 0.000762 | 4 |
| BP | GO:0051384 | response to glucocorticoid | 0.000195 | 0.000795 | 5 |
| BP | GO:0002824 | positive regulation of adaptive immune response based on somatic recombination of immune receptors built from immunoglobulin superfamily domains | 0.000196 | 0.000797 | 4 |
| BP | GO:0015850 | organic hydroxy compound transport | 0.000207 | 0.000837 | 6 |
| BP | GO:0034142 | toll-like receptor 4 signaling pathway | 0.000208 | 0.000839 | 3 |
| BP | GO:0046677 | response to antibiotic | 0.00021 | 0.000845 | 7 |
| BP | GO:0071674 | mononuclear cell migration | 0.000217 | 0.000869 | 4 |
| BP | GO:0014074 | response to purine-containing compound | 0.000222 | 0.000888 | 5 |
| BP | GO:0002708 | positive regulation of lymphocyte mediated immunity | 0.000228 | 0.000902 | 4 |
| BP | GO:0002711 | positive regulation of T cell mediated immunity | 0.000229 | 0.000902 | 3 |
| BP | GO:0032735 | positive regulation of interleukin-12 production | 0.000229 | 0.000902 | 3 |
| BP | GO:0071354 | cellular response to interleukin-6 | 0.000229 | 0.000902 | 3 |
| BP | GO:0090322 | regulation of superoxide metabolic process | 0.000229 | 0.000902 | 3 |
| BP | GO:0002821 | positive regulation of adaptive immune response | 0.000239 | 0.000935 | 4 |
| BP | GO:1901888 | regulation of cell junction assembly | 0.000239 | 0.000935 | 4 |
| BP | GO:0007159 | leukocyte cell-cell adhesion | 0.000248 | 0.000969 | 7 |
| BP | GO:0050810 | regulation of steroid biosynthetic process | 0.00025 | 0.000972 | 4 |
| BP | GO:0070098 | chemokine-mediated signaling pathway | 0.00025 | 0.000972 | 4 |
| BP | GO:0050714 | positive regulation of protein secretion | 0.000261 | 0.001009 | 6 |
| BP | GO:0042446 | hormone biosynthetic process | 0.000262 | 0.001012 | 4 |
| BP | GO:1901617 | organic hydroxy compound biosynthetic process | 0.000273 | 0.001043 | 6 |
| BP | GO:1903426 | regulation of reactive oxygen species biosynthetic process | 0.000275 | 0.001043 | 4 |
| BP | GO:0002369 | T cell cytokine production | 0.000275 | 0.001043 | 3 |
| BP | GO:0097421 | liver regeneration | 0.000275 | 0.001043 | 3 |
| BP | GO:1900015 | regulation of cytokine production involved in inflammatory response | 0.000275 | 0.001043 | 3 |
| BP | GO:1900026 | positive regulation of substrate adhesion-dependent cell spreading | 0.000275 | 0.001043 | 3 |
| BP | GO:0010742 | macrophage derived foam cell differentiation | 0.0003 | 0.001132 | 3 |
| BP | GO:0090077 | foam cell differentiation | 0.0003 | 0.001132 | 3 |
| BP | GO:0002690 | positive regulation of leukocyte chemotaxis | 0.000314 | 0.001177 | 4 |
| BP | GO:0045639 | positive regulation of myeloid cell differentiation | 0.000314 | 0.001177 | 4 |
| BP | GO:1904951 | positive regulation of establishment of protein localization | 0.000315 | 0.001177 | 8 |
| BP | GO:0051098 | regulation of binding | 0.000318 | 0.001187 | 7 |
| BP | GO:0045862 | positive regulation of proteolysis | 0.000324 | 0.001204 | 7 |
| BP | GO:0034122 | negative regulation of toll-like receptor signaling pathway | 0.000326 | 0.001208 | 3 |
| BP | GO:0070741 | response to interleukin-6 | 0.000326 | 0.001208 | 3 |
| BP | GO:0034614 | cellular response to reactive oxygen species | 0.000348 | 0.001285 | 5 |
| BP | GO:0043491 | protein kinase B signaling | 0.000354 | 0.001293 | 6 |
| BP | GO:0002534 | cytokine production involved in inflammatory response | 0.000354 | 0.001293 | 3 |
| BP | GO:0016486 | peptide hormone processing | 0.000354 | 0.001293 | 3 |
| BP | GO:0045742 | positive regulation of epidermal growth factor receptor signaling pathway | 0.000354 | 0.001293 | 3 |
| BP | GO:0033273 | response to vitamin | 0.000358 | 0.001303 | 4 |
| BP | GO:0030316 | osteoclast differentiation | 0.000373 | 0.001351 | 4 |
| BP | GO:0043154 | negative regulation of cysteine-type endopeptidase activity involved in apoptotic process | 0.000373 | 0.001351 | 4 |
| BP | GO:1902105 | regulation of leukocyte differentiation | 0.000385 | 0.001391 | 6 |
| BP | GO:0051341 | regulation of oxidoreductase activity | 0.000389 | 0.001401 | 4 |
| BP | GO:0022409 | positive regulation of cell-cell adhesion | 0.000393 | 0.001412 | 6 |
| BP | GO:0002793 | positive regulation of peptide secretion | 0.000409 | 0.001468 | 6 |
| BP | GO:0001990 | regulation of systemic arterial blood pressure by hormone | 0.000414 | 0.001469 | 3 |
| BP | GO:0030225 | macrophage differentiation | 0.000414 | 0.001469 | 3 |
| BP | GO:0061028 | establishment of endothelial barrier | 0.000414 | 0.001469 | 3 |
| BP | GO:1901186 | positive regulation of ERBB signaling pathway | 0.000414 | 0.001469 | 3 |
| BP | GO:0002474 | antigen processing and presentation of peptide antigen via MHC class I | 0.000422 | 0.001493 | 4 |
| BP | GO:0051099 | positive regulation of binding | 0.000434 | 0.00153 | 5 |
| BP | GO:0062012 | regulation of small molecule metabolic process | 0.000435 | 0.00153 | 8 |
| BP | GO:0014911 | positive regulation of smooth muscle cell migration | 0.000447 | 0.001564 | 3 |
| BP | GO:0033628 | regulation of cell adhesion mediated by integrin | 0.000447 | 0.001564 | 3 |
| BP | GO:1901652 | response to peptide | 0.000472 | 0.001647 | 8 |
| BP | GO:0002699 | positive regulation of immune effector process | 0.000496 | 0.001726 | 5 |
| BP | GO:0032309 | icosanoid secretion | 0.000516 | 0.001793 | 3 |
| BP | GO:0002705 | positive regulation of leukocyte mediated immunity | 0.000534 | 0.001849 | 4 |
| BP | GO:0060326 | cell chemotaxis | 0.000541 | 0.001869 | 6 |
| BP | GO:0050731 | positive regulation of peptidyl-tyrosine phosphorylation | 0.000549 | 0.001894 | 5 |
| BP | GO:0010883 | regulation of lipid storage | 0.000554 | 0.001896 | 3 |
| BP | GO:0045601 | regulation of endothelial cell differentiation | 0.000554 | 0.001896 | 3 |
| BP | GO:0014066 | regulation of phosphatidylinositol 3-kinase signaling | 0.000554 | 0.001896 | 4 |
| BP | GO:0071887 | leukocyte apoptotic process | 0.000575 | 0.001963 | 4 |
| BP | GO:0006775 | fat-soluble vitamin metabolic process | 0.000592 | 0.002017 | 3 |
| BP | GO:0008630 | intrinsic apoptotic signaling pathway in response to DNA damage | 0.000597 | 0.002026 | 4 |
| BP | GO:0009157 | deoxyribonucleoside monophosphate biosynthetic process | 0.000608 | 0.002043 | 2 |
| BP | GO:0042756 | drinking behavior | 0.000608 | 0.002043 | 2 |
| BP | GO:1901033 | positive regulation of response to reactive oxygen species | 0.000608 | 0.002043 | 2 |
| BP | GO:2000109 | regulation of macrophage apoptotic process | 0.000608 | 0.002043 | 2 |
| BP | GO:1901214 | regulation of neuron death | 0.000629 | 0.00211 | 6 |
| BP | GO:0071715 | icosanoid transport | 0.000633 | 0.002113 | 3 |
| BP | GO:1901571 | fatty acid derivative transport | 0.000633 | 0.002113 | 3 |
| BP | GO:0048002 | antigen processing and presentation of peptide antigen | 0.00067 | 0.002229 | 5 |
| BP | GO:0061098 | positive regulation of protein tyrosine kinase activity | 0.000675 | 0.002237 | 3 |
| BP | GO:1901031 | regulation of response to reactive oxygen species | 0.000675 | 0.002237 | 3 |
| BP | GO:0010811 | positive regulation of cell-substrate adhesion | 0.000689 | 0.002276 | 4 |
| BP | GO:0051353 | positive regulation of oxidoreductase activity | 0.00072 | 0.002372 | 3 |
| BP | GO:0016264 | gap junction assembly | 0.000741 | 0.002419 | 2 |
| BP | GO:0031652 | positive regulation of heat generation | 0.000741 | 0.002419 | 2 |
| BP | GO:0045073 | regulation of chemokine biosynthetic process | 0.000741 | 0.002419 | 2 |
| BP | GO:0045351 | type I interferon biosynthetic process | 0.000741 | 0.002419 | 2 |
| BP | GO:0006953 | acute-phase response | 0.000766 | 0.002481 | 3 |
| BP | GO:0048260 | positive regulation of receptor-mediated endocytosis | 0.000766 | 0.002481 | 3 |
| BP | GO:1905953 | negative regulation of lipid localization | 0.000766 | 0.002481 | 3 |
| BP | GO:0045766 | positive regulation of angiogenesis | 0.000772 | 0.002497 | 5 |
| BP | GO:0002688 | regulation of leukocyte chemotaxis | 0.000791 | 0.002538 | 4 |
| BP | GO:0032675 | regulation of interleukin-6 production | 0.000791 | 0.002538 | 4 |
| BP | GO:2000117 | negative regulation of cysteine-type endopeptidase activity | 0.000791 | 0.002538 | 4 |
| BP | GO:0003044 | regulation of systemic arterial blood pressure mediated by a chemical signal | 0.000813 | 0.002586 | 3 |
| BP | GO:0010518 | positive regulation of phospholipase activity | 0.000813 | 0.002586 | 3 |
| BP | GO:1900024 | regulation of substrate adhesion-dependent cell spreading | 0.000813 | 0.002586 | 3 |
| BP | GO:2000107 | negative regulation of leukocyte apoptotic process | 0.000813 | 0.002586 | 3 |
| BP | GO:0050663 | cytokine secretion | 0.000828 | 0.002626 | 5 |
| BP | GO:0007568 | aging | 0.000855 | 0.002706 | 6 |
| BP | GO:0032663 | regulation of interleukin-2 production | 0.000863 | 0.002712 | 3 |
| BP | GO:2001238 | positive regulation of extrinsic apoptotic signaling pathway | 0.000863 | 0.002712 | 3 |
| BP | GO:0007043 | cell-cell junction assembly | 0.000873 | 0.002712 | 4 |
| BP | GO:0032680 | regulation of tumor necrosis factor production | 0.000873 | 0.002712 | 4 |
| BP | GO:0001660 | fever generation | 0.000887 | 0.002712 | 2 |
| BP | GO:0009265 | 2'-deoxyribonucleotide biosynthetic process | 0.000887 | 0.002712 | 2 |
| BP | GO:0030656 | regulation of vitamin metabolic process | 0.000887 | 0.002712 | 2 |
| BP | GO:0032490 | detection of molecule of bacterial origin | 0.000887 | 0.002712 | 2 |
| BP | GO:0042033 | chemokine biosynthetic process | 0.000887 | 0.002712 | 2 |
| BP | GO:0045414 | regulation of interleukin-8 biosynthetic process | 0.000887 | 0.002712 | 2 |
| BP | GO:0046385 | deoxyribose phosphate biosynthetic process | 0.000887 | 0.002712 | 2 |
| BP | GO:0050755 | chemokine metabolic process | 0.000887 | 0.002712 | 2 |
| BP | GO:0051798 | positive regulation of hair follicle development | 0.000887 | 0.002712 | 2 |
| BP | GO:0071888 | macrophage apoptotic process | 0.000887 | 0.002712 | 2 |
| BP | GO:2000121 | regulation of removal of superoxide radicals | 0.000887 | 0.002712 | 2 |
| BP | GO:0051101 | regulation of DNA binding | 0.000932 | 0.002844 | 4 |
| BP | GO:0006874 | cellular calcium ion homeostasis | 0.000954 | 0.002905 | 7 |
| BP | GO:0032640 | tumor necrosis factor production | 0.000962 | 0.002923 | 4 |
| BP | GO:0048008 | platelet-derived growth factor receptor signaling pathway | 0.000968 | 0.002933 | 3 |
| BP | GO:1903555 | regulation of tumor necrosis factor superfamily cytokine production | 0.000993 | 0.003004 | 4 |
| BP | GO:0002709 | regulation of T cell mediated immunity | 0.001023 | 0.003086 | 3 |
| BP | GO:0009162 | deoxyribonucleoside monophosphate metabolic process | 0.001046 | 0.003086 | 2 |
| BP | GO:0017014 | protein nitrosylation | 0.001046 | 0.003086 | 2 |
| BP | GO:0018119 | peptidyl-cysteine S-nitrosylation | 0.001046 | 0.003086 | 2 |
| BP | GO:0031650 | regulation of heat generation | 0.001046 | 0.003086 | 2 |
| BP | GO:0032310 | prostaglandin secretion | 0.001046 | 0.003086 | 2 |
| BP | GO:0042228 | interleukin-8 biosynthetic process | 0.001046 | 0.003086 | 2 |
| BP | GO:0042635 | positive regulation of hair cycle | 0.001046 | 0.003086 | 2 |
| BP | GO:0042976 | activation of Janus kinase activity | 0.001046 | 0.003086 | 2 |
| BP | GO:0045651 | positive regulation of macrophage differentiation | 0.001046 | 0.003086 | 2 |
| BP | GO:0070431 | nucleotide-binding oligomerization domain containing 2 signaling pathway | 0.001046 | 0.003086 | 2 |
| BP | GO:0051222 | positive regulation of protein transport | 0.001051 | 0.003094 | 7 |
| BP | GO:0002822 | regulation of adaptive immune response based on somatic recombination of immune receptors built from immunoglobulin superfamily domains | 0.001057 | 0.003108 | 4 |
| BP | GO:0001885 | endothelial cell development | 0.00108 | 0.003168 | 3 |
| BP | GO:0048732 | gland development | 0.001094 | 0.003201 | 7 |
| BP | GO:0055074 | calcium ion homeostasis | 0.001109 | 0.003238 | 7 |
| BP | GO:0032722 | positive regulation of chemokine production | 0.00114 | 0.003306 | 3 |
| BP | GO:0043388 | positive regulation of DNA binding | 0.00114 | 0.003306 | 3 |
| BP | GO:0070527 | platelet aggregation | 0.00114 | 0.003306 | 3 |
| BP | GO:0019058 | viral life cycle | 0.001157 | 0.003341 | 6 |
| BP | GO:0070997 | neuron death | 0.001157 | 0.003341 | 6 |
| BP | GO:0071706 | tumor necrosis factor superfamily cytokine production | 0.001159 | 0.003341 | 4 |
| BP | GO:0002706 | regulation of lymphocyte mediated immunity | 0.001194 | 0.003428 | 4 |
| BP | GO:0051291 | protein heterooligomerization | 0.001194 | 0.003428 | 4 |
| BP | GO:0002637 | regulation of immunoglobulin production | 0.001201 | 0.00344 | 3 |
| BP | GO:0051770 | positive regulation of nitric-oxide synthase biosynthetic process | 0.001217 | 0.003456 | 2 |
| BP | GO:0090153 | regulation of sphingolipid biosynthetic process | 0.001217 | 0.003456 | 2 |
| BP | GO:1905038 | regulation of membrane lipid metabolic process | 0.001217 | 0.003456 | 2 |
| BP | GO:2000303 | regulation of ceramide biosynthetic process | 0.001217 | 0.003456 | 2 |
| BP | GO:0032355 | response to estradiol | 0.00123 | 0.003487 | 4 |
| BP | GO:1904018 | positive regulation of vasculature development | 0.001251 | 0.003538 | 5 |
| BP | GO:0032623 | interleukin-2 production | 0.001264 | 0.003569 | 3 |
| BP | GO:0018209 | peptidyl-serine modification | 0.001274 | 0.003588 | 6 |
| BP | GO:0006066 | alcohol metabolic process | 0.001315 | 0.003695 | 6 |
| BP | GO:0010517 | regulation of phospholipase activity | 0.00133 | 0.003722 | 3 |
| BP | GO:0032768 | regulation of monooxygenase activity | 0.00133 | 0.003722 | 3 |
| BP | GO:0006720 | isoprenoid metabolic process | 0.001343 | 0.003751 | 4 |
| BP | GO:0009263 | deoxyribonucleotide biosynthetic process | 0.001401 | 0.003889 | 2 |
| BP | GO:0051044 | positive regulation of membrane protein ectodomain proteolysis | 0.001401 | 0.003889 | 2 |
| BP | GO:2000846 | regulation of corticosteroid hormone secretion | 0.001401 | 0.003889 | 2 |
| BP | GO:0019882 | antigen processing and presentation | 0.001413 | 0.003914 | 5 |
| BP | GO:0001101 | response to acid chemical | 0.001422 | 0.003916 | 6 |
| BP | GO:0014065 | phosphatidylinositol 3-kinase signaling | 0.001422 | 0.003916 | 4 |
| BP | GO:0050728 | negative regulation of inflammatory response | 0.001422 | 0.003916 | 4 |
| BP | GO:0002819 | regulation of adaptive immune response | 0.001463 | 0.004003 | 4 |
| BP | GO:0045471 | response to ethanol | 0.001463 | 0.004003 | 4 |
| BP | GO:0045807 | positive regulation of endocytosis | 0.001463 | 0.004003 | 4 |
| BP | GO:1902107 | positive regulation of leukocyte differentiation | 0.001547 | 0.004224 | 4 |
| BP | GO:0072503 | cellular divalent inorganic cation homeostasis | 0.001553 | 0.004233 | 7 |
| BP | GO:0000302 | response to reactive oxygen species | 0.00159 | 0.004276 | 5 |
| BP | GO:0009130 | pyrimidine nucleoside monophosphate biosynthetic process | 0.001597 | 0.004276 | 2 |
| BP | GO:0010663 | positive regulation of striated muscle cell apoptotic process | 0.001597 | 0.004276 | 2 |
| BP | GO:0010666 | positive regulation of cardiac muscle cell apoptotic process | 0.001597 | 0.004276 | 2 |
| BP | GO:0010744 | positive regulation of macrophage derived foam cell differentiation | 0.001597 | 0.004276 | 2 |
| BP | GO:0015732 | prostaglandin transport | 0.001597 | 0.004276 | 2 |
| BP | GO:0033033 | negative regulation of myeloid cell apoptotic process | 0.001597 | 0.004276 | 2 |
| BP | GO:0035930 | corticosteroid hormone secretion | 0.001597 | 0.004276 | 2 |
| BP | GO:1903209 | positive regulation of oxidative stress-induced cell death | 0.001597 | 0.004276 | 2 |
| BP | GO:0002548 | monocyte chemotaxis | 0.001613 | 0.004292 | 3 |
| BP | GO:0032729 | positive regulation of interferon-gamma production | 0.001613 | 0.004292 | 3 |
| BP | GO:0060193 | positive regulation of lipase activity | 0.001613 | 0.004292 | 3 |
| BP | GO:0030260 | entry into host cell | 0.001634 | 0.004314 | 4 |
| BP | GO:0044409 | entry into host | 0.001634 | 0.004314 | 4 |
| BP | GO:0051806 | entry into cell of other organism involved in symbiotic interaction | 0.001634 | 0.004314 | 4 |
| BP | GO:0051828 | entry into other organism involved in symbiotic interaction | 0.001634 | 0.004314 | 4 |
| BP | GO:0071560 | cellular response to transforming growth factor beta stimulus | 0.001652 | 0.004354 | 5 |
| BP | GO:0051924 | regulation of calcium ion transport | 0.001684 | 0.00443 | 5 |
| BP | GO:0031667 | response to nutrient levels | 0.001714 | 0.004497 | 7 |
| BP | GO:0097237 | cellular response to toxic substance | 0.001716 | 0.004497 | 5 |
| BP | GO:0006766 | vitamin metabolic process | 0.001724 | 0.004509 | 4 |
| BP | GO:0019915 | lipid storage | 0.001767 | 0.004604 | 3 |
| BP | GO:0046889 | positive regulation of lipid biosynthetic process | 0.001767 | 0.004604 | 3 |
| BP | GO:0071559 | response to transforming growth factor beta | 0.001782 | 0.004635 | 5 |
| BP | GO:0009129 | pyrimidine nucleoside monophosphate metabolic process | 0.001806 | 0.004668 | 2 |
| BP | GO:0009200 | deoxyribonucleoside triphosphate metabolic process | 0.001806 | 0.004668 | 2 |
| BP | GO:0010888 | negative regulation of lipid storage | 0.001806 | 0.004668 | 2 |
| BP | GO:0050730 | regulation of peptidyl-tyrosine phosphorylation | 0.00192 | 0.004955 | 5 |
| BP | GO:0072507 | divalent inorganic cation homeostasis | 0.001934 | 0.004982 | 7 |
| BP | GO:0010770 | positive regulation of cell morphogenesis involved in differentiation | 0.001966 | 0.005053 | 4 |
| BP | GO:0006606 | protein import into nucleus | 0.002016 | 0.005156 | 4 |
| BP | GO:0000185 | activation of MAPKKK activity | 0.002027 | 0.005156 | 2 |
| BP | GO:0051767 | nitric-oxide synthase biosynthetic process | 0.002027 | 0.005156 | 2 |
| BP | GO:0051769 | regulation of nitric-oxide synthase biosynthetic process | 0.002027 | 0.005156 | 2 |
| BP | GO:2000831 | regulation of steroid hormone secretion | 0.002027 | 0.005156 | 2 |
| BP | GO:0043270 | positive regulation of ion transport | 0.002028 | 0.005156 | 5 |
| BP | GO:0002521 | leukocyte differentiation | 0.002125 | 0.005392 | 7 |
| BP | GO:0006970 | response to osmotic stress | 0.002193 | 0.005553 | 3 |
| BP | GO:0001818 | negative regulation of cytokine production | 0.002219 | 0.005608 | 5 |
| BP | GO:0002544 | chronic inflammatory response | 0.002259 | 0.005669 | 2 |
| BP | GO:0033622 | integrin activation | 0.002259 | 0.005669 | 2 |
| BP | GO:0035994 | response to muscle stretch | 0.002259 | 0.005669 | 2 |
| BP | GO:0098581 | detection of external biotic stimulus | 0.002259 | 0.005669 | 2 |
| BP | GO:0060627 | regulation of vesicle-mediated transport | 0.002304 | 0.005771 | 7 |
| CC | GO:0009897 | external side of plasma membrane | 1.98E-11 | 1.44E-09 | 13 |
| CC | GO:0045121 | membrane raft | 5.73E-10 | 1.44E-08 | 12 |
| CC | GO:0098857 | membrane microdomain | 5.95E-10 | 1.44E-08 | 12 |
| CC | GO:0098589 | membrane region | 8.93E-10 | 1.62E-08 | 12 |
| CC | GO:0005788 | endoplasmic reticulum lumen | 6.68E-09 | 9.70E-08 | 11 |
| CC | GO:0008385 | IkappaB kinase complex | 4.59E-08 | 4.76E-07 | 4 |
| CC | GO:0035631 | CD40 receptor complex | 4.59E-08 | 4.76E-07 | 4 |
| CC | GO:0072562 | blood microparticle | 2.56E-07 | 2.32E-06 | 8 |
| CC | GO:0043235 | receptor complex | 7.08E-07 | 5.50E-06 | 10 |
| CC | GO:0031091 | platelet alpha granule | 7.57E-07 | 5.50E-06 | 6 |
| CC | GO:0031093 | platelet alpha granule lumen | 3.83E-06 | 2.53E-05 | 5 |
| CC | GO:0010008 | endosome membrane | 2.79E-05 | 0.000164 | 9 |
| CC | GO:0005796 | Golgi lumen | 3.15E-05 | 0.000164 | 5 |
| CC | GO:0044445 | cytosolic part | 3.17E-05 | 0.000164 | 7 |
| CC | GO:0044440 | endosomal part | 5.84E-05 | 0.000283 | 9 |
| CC | GO:1902554 | serine/threonine protein kinase complex | 0.000231 | 0.00105 | 4 |
| CC | GO:1902911 | protein kinase complex | 0.000414 | 0.00177 | 4 |
| CC | GO:0005938 | cell cortex | 0.000613 | 0.002473 | 6 |
| CC | GO:0034774 | secretory granule lumen | 0.000934 | 0.003572 | 6 |
| CC | GO:0060205 | cytoplasmic vesicle lumen | 0.001218 | 0.004278 | 6 |
| CC | GO:0031983 | vesicle lumen | 0.001237 | 0.004278 | 6 |
| MF | GO:0032813 | tumor necrosis factor receptor superfamily binding | 1.87E-08 | 3.01E-06 | 6 |
| MF | GO:0008236 | serine-type peptidase activity | 7.65E-08 | 4.09E-06 | 10 |
| MF | GO:0017171 | serine hydrolase activity | 9.05E-08 | 4.09E-06 | 10 |
| MF | GO:0005164 | tumor necrosis factor receptor binding | 1.02E-07 | 4.09E-06 | 5 |
| MF | GO:0004175 | endopeptidase activity | 1.72E-07 | 5.54E-06 | 12 |
| MF | GO:0004252 | serine-type endopeptidase activity | 3.57E-07 | 9.58E-06 | 9 |
| MF | GO:0031625 | ubiquitin protein ligase binding | 2.22E-06 | 5.11E-05 | 9 |
| MF | GO:0044389 | ubiquitin-like protein ligase binding | 3.19E-06 | 6.43E-05 | 9 |
| MF | GO:0043177 | organic acid binding | 7.34E-06 | 0.000131 | 7 |
| MF | GO:0005126 | cytokine receptor binding | 9.30E-06 | 0.00015 | 8 |
| MF | GO:0097110 | scaffold protein binding | 7.11E-05 | 0.001028 | 4 |
| MF | GO:0031406 | carboxylic acid binding | 7.66E-05 | 0.001028 | 6 |
| MF | GO:0048037 | cofactor binding | 8.63E-05 | 0.001069 | 9 |
| MF | GO:0002020 | protease binding | 0.000143 | 0.001646 | 5 |
| MF | GO:0001530 | lipopolysaccharide binding | 0.000212 | 0.002275 | 3 |
| MF | GO:0008201 | heparin binding | 0.000319 | 0.003209 | 5 |
| MF | GO:0004867 | serine-type endopeptidase inhibitor activity | 0.000449 | 0.004257 | 4 |
| MF | GO:0004866 | endopeptidase inhibitor activity | 0.000483 | 0.004306 | 5 |
| MF | GO:0015026 | coreceptor activity | 0.000525 | 0.004306 | 3 |
| MF | GO:0061135 | endopeptidase regulator activity | 0.000564 | 0.004306 | 5 |
| MF | GO:0030414 | peptidase inhibitor activity | 0.000609 | 0.004306 | 5 |
| MF | GO:0019957 | C-C chemokine binding | 0.000615 | 0.004306 | 2 |
| MF | GO:0070513 | death domain binding | 0.000615 | 0.004306 | 2 |
| MF | GO:0031996 | thioesterase binding | 0.00075 | 0.004824 | 2 |
| MF | GO:1990825 | sequence-specific mRNA binding | 0.00075 | 0.004824 | 2 |
| MF | GO:0050661 | NADP binding | 0.000779 | 0.004824 | 3 |
| MF | GO:0005542 | folic acid binding | 0.000897 | 0.005162 | 2 |
| MF | GO:0042975 | peroxisome proliferator activated receptor binding | 0.000897 | 0.005162 | 2 |
