# Supplementary material for: Investigating the multi-target pharmacological mechanism of danhong injection acting on unstable angina by combined network pharmacology and molecular docking
Source: BMC Complement Med Ther. 2020 Mar 2;20:66. doi: 10.1186/s12906-020-2853-5 (PMC7076845; doi:10.1186/s12906-020-2853-5)
Supplement: Supplementary file 3 — Additional file 3 Supplementary 3. Table S3. The information of GO enrichment analysis for each cluster. [file 12906_2020_2853_MOESM3_ESM.docx]

Table S3. The information of GO enrichment analysis for each cluster

Module 1:

| ONTOLOGY | ID | Description | pvalue | qvalue | Count |
| --- | --- | --- | --- | --- | --- |
| BP | GO:0007249 | I-kappaB kinase/NF-kappaB signaling | 3.25E-32 | 1.45E-29 | 17 |
| BP | GO:0051092 | positive regulation of NF-kappaB transcription factor activity | 2.14E-30 | 4.80E-28 | 15 |
| BP | GO:0070423 | nucleotide-binding oligomerization domain containing signaling pathway | 8.10E-27 | 1.05E-24 | 11 |
| BP | GO:0035872 | nucleotide-binding domain, leucine rich repeat containing receptor signaling pathway | 1.14E-26 | 1.05E-24 | 11 |
| BP | GO:0051091 | positive regulation of DNA binding transcription factor activity | 1.18E-26 | 1.05E-24 | 15 |
| BP | GO:0002753 | cytoplasmic pattern recognition receptor signaling pathway | 3.22E-24 | 2.40E-22 | 11 |
| BP | GO:0043123 | positive regulation of I-kappaB kinase/NF-kappaB signaling | 6.97E-24 | 4.45E-22 | 13 |
| BP | GO:0051090 | regulation of DNA binding transcription factor activity | 3.90E-23 | 2.18E-21 | 15 |
| BP | GO:0002224 | toll-like receptor signaling pathway | 1.45E-22 | 7.23E-21 | 12 |
| BP | GO:0031349 | positive regulation of defense response | 2.19E-22 | 9.78E-21 | 15 |
| BP | GO:0043122 | regulation of I-kappaB kinase/NF-kappaB signaling | 3.34E-22 | 1.36E-20 | 13 |
| BP | GO:0010803 | regulation of tumor necrosis factor-mediated signaling pathway | 2.13E-21 | 7.95E-20 | 10 |
| BP | GO:0051403 | stress-activated MAPK cascade | 3.65E-21 | 1.26E-19 | 13 |
| BP | GO:0002221 | pattern recognition receptor signaling pathway | 5.53E-21 | 1.77E-19 | 12 |
| BP | GO:0002758 | innate immune response-activating signal transduction | 7.88E-21 | 2.35E-19 | 13 |
| BP | GO:0031098 | stress-activated protein kinase signaling cascade | 1.43E-20 | 3.99E-19 | 13 |
| BP | GO:0002218 | activation of innate immune response | 1.78E-20 | 4.69E-19 | 13 |
| BP | GO:0045089 | positive regulation of innate immune response | 1.45E-19 | 3.61E-18 | 13 |
| BP | GO:0070498 | interleukin-1-mediated signaling pathway | 4.03E-19 | 9.50E-18 | 10 |
| BP | GO:0033209 | tumor necrosis factor-mediated signaling pathway | 6.83E-19 | 1.53E-17 | 11 |
| BP | GO:0071356 | cellular response to tumor necrosis factor | 1.56E-18 | 3.32E-17 | 12 |
| BP | GO:0045088 | regulation of innate immune response | 1.77E-18 | 3.59E-17 | 13 |
| BP | GO:0034612 | response to tumor necrosis factor | 3.27E-18 | 6.36E-17 | 12 |
| BP | GO:0002223 | stimulatory C-type lectin receptor signaling pathway | 3.47E-18 | 6.46E-17 | 10 |
| BP | GO:0002220 | innate immune response activating cell surface receptor signaling pathway | 4.45E-18 | 7.96E-17 | 10 |
| BP | GO:0071347 | cellular response to interleukin-1 | 9.64E-17 | 1.66E-15 | 10 |
| BP | GO:0001959 | regulation of cytokine-mediated signaling pathway | 1.09E-16 | 1.80E-15 | 10 |
| BP | GO:0038095 | Fc-epsilon receptor signaling pathway | 1.63E-16 | 2.61E-15 | 10 |
| BP | GO:0060759 | regulation of response to cytokine stimulus | 1.73E-16 | 2.66E-15 | 10 |
| BP | GO:0030522 | intracellular receptor signaling pathway | 1.82E-16 | 2.71E-15 | 11 |
| BP | GO:0035666 | TRIF-dependent toll-like receptor signaling pathway | 2.84E-16 | 4.09E-15 | 7 |
| BP | GO:0070555 | response to interleukin-1 | 3.33E-16 | 4.66E-15 | 10 |
| BP | GO:0007254 | JNK cascade | 5.05E-16 | 6.84E-15 | 10 |
| BP | GO:0002756 | MyD88-independent toll-like receptor signaling pathway | 6.11E-16 | 8.04E-15 | 7 |
| BP | GO:0070266 | necroptotic process | 1.51E-15 | 1.93E-14 | 7 |
| BP | GO:0000187 | activation of MAPK activity | 3.45E-15 | 4.29E-14 | 9 |
| BP | GO:0097300 | programmed necrotic cell death | 4.06E-15 | 4.83E-14 | 7 |
| BP | GO:0038093 | Fc receptor signaling pathway | 4.11E-15 | 4.83E-14 | 10 |
| BP | GO:0043406 | positive regulation of MAP kinase activity | 9.74E-15 | 1.12E-13 | 10 |
| BP | GO:0070265 | necrotic cell death | 2.40E-14 | 2.69E-13 | 7 |
| BP | GO:0050852 | T cell receptor signaling pathway | 5.77E-14 | 6.30E-13 | 9 |
| BP | GO:0032147 | activation of protein kinase activity | 7.42E-14 | 7.90E-13 | 10 |
| BP | GO:0043405 | regulation of MAP kinase activity | 1.19E-13 | 1.24E-12 | 10 |
| BP | GO:0038061 | NIK/NF-kappaB signaling | 1.24E-13 | 1.26E-12 | 8 |
| BP | GO:0071902 | positive regulation of protein serine/threonine kinase activity | 1.64E-13 | 1.63E-12 | 10 |
| BP | GO:0002755 | MyD88-dependent toll-like receptor signaling pathway | 5.65E-13 | 5.49E-12 | 6 |
| BP | GO:0007252 | I-kappaB phosphorylation | 1.29E-12 | 1.21E-11 | 5 |
| BP | GO:0050851 | antigen receptor-mediated signaling pathway | 1.30E-12 | 1.21E-11 | 9 |
| BP | GO:0016579 | protein deubiquitination | 1.72E-12 | 1.56E-11 | 9 |
| BP | GO:0002429 | immune response-activating cell surface receptor signaling pathway | 1.74E-12 | 1.56E-11 | 10 |
| BP | GO:0070646 | protein modification by small protein removal | 2.71E-12 | 2.37E-11 | 9 |
| BP | GO:0002768 | immune response-regulating cell surface receptor signaling pathway | 3.44E-12 | 2.96E-11 | 10 |
| BP | GO:0060544 | regulation of necroptotic process | 6.66E-12 | 5.62E-11 | 5 |
| BP | GO:0001819 | positive regulation of cytokine production | 3.80E-11 | 3.15E-10 | 9 |
| BP | GO:0010939 | regulation of necrotic cell death | 6.09E-11 | 4.95E-10 | 5 |
| BP | GO:0046330 | positive regulation of JNK cascade | 1.24E-09 | 9.87E-09 | 6 |
| BP | GO:0002718 | regulation of cytokine production involved in immune response | 3.74E-09 | 2.94E-08 | 5 |
| BP | GO:0032874 | positive regulation of stress-activated MAPK cascade | 4.06E-09 | 3.13E-08 | 6 |
| BP | GO:0070304 | positive regulation of stress-activated protein kinase signaling cascade | 4.22E-09 | 3.20E-08 | 6 |
| BP | GO:0043507 | positive regulation of JUN kinase activity | 5.06E-09 | 3.77E-08 | 5 |
| BP | GO:0046328 | regulation of JNK cascade | 7.99E-09 | 5.86E-08 | 6 |
| BP | GO:0032496 | response to lipopolysaccharide | 1.16E-08 | 8.40E-08 | 7 |
| BP | GO:0008625 | extrinsic apoptotic signaling pathway via death domain receptors | 1.20E-08 | 8.55E-08 | 5 |
| BP | GO:0002367 | cytokine production involved in immune response | 1.36E-08 | 9.35E-08 | 5 |
| BP | GO:0043506 | regulation of JUN kinase activity | 1.36E-08 | 9.35E-08 | 5 |
| BP | GO:0002237 | response to molecule of bacterial origin | 1.68E-08 | 1.14E-07 | 7 |
| BP | GO:0002720 | positive regulation of cytokine production involved in immune response | 3.40E-08 | 2.27E-07 | 4 |
| BP | GO:0032872 | regulation of stress-activated MAPK cascade | 3.46E-08 | 2.28E-07 | 6 |
| BP | GO:0070302 | regulation of stress-activated protein kinase signaling cascade | 3.56E-08 | 2.31E-07 | 6 |
| BP | GO:0002700 | regulation of production of molecular mediator of immune response | 6.25E-08 | 3.99E-07 | 5 |
| BP | GO:1901224 | positive regulation of NIK/NF-kappaB signaling | 7.10E-08 | 4.47E-07 | 4 |
| BP | GO:1901550 | regulation of endothelial cell development | 1.62E-07 | 9.95E-07 | 3 |
| BP | GO:1903140 | regulation of establishment of endothelial barrier | 1.62E-07 | 9.95E-07 | 3 |
| BP | GO:0031663 | lipopolysaccharide-mediated signaling pathway | 1.81E-07 | 1.09E-06 | 4 |
| BP | GO:0097296 | activation of cysteine-type endopeptidase activity involved in apoptotic signaling pathway | 2.11E-07 | 1.26E-06 | 3 |
| BP | GO:0002726 | positive regulation of T cell cytokine production | 2.68E-07 | 1.58E-06 | 3 |
| BP | GO:1901222 | regulation of NIK/NF-kappaB signaling | 2.78E-07 | 1.61E-06 | 4 |
| BP | GO:0002703 | regulation of leukocyte mediated immunity | 4.15E-07 | 2.38E-06 | 5 |
| BP | GO:0071222 | cellular response to lipopolysaccharide | 4.94E-07 | 2.80E-06 | 5 |
| BP | GO:0051023 | regulation of immunoglobulin secretion | 5.00E-07 | 2.80E-06 | 3 |
| BP | GO:0002699 | positive regulation of immune effector process | 5.08E-07 | 2.81E-06 | 5 |
| BP | GO:0007250 | activation of NF-kappaB-inducing kinase activity | 6.00E-07 | 3.20E-06 | 3 |
| BP | GO:1900409 | positive regulation of cellular response to oxidative stress | 6.00E-07 | 3.20E-06 | 3 |
| BP | GO:2001269 | positive regulation of cysteine-type endopeptidase activity involved in apoptotic signaling pathway | 6.00E-07 | 3.20E-06 | 3 |
| BP | GO:0071219 | cellular response to molecule of bacterial origin | 6.17E-07 | 3.25E-06 | 5 |
| BP | GO:0002702 | positive regulation of production of molecular mediator of immune response | 6.50E-07 | 3.38E-06 | 4 |
| BP | GO:0002824 | positive regulation of adaptive immune response based on somatic recombination of immune receptors built from immunoglobulin superfamily domains | 7.63E-07 | 3.92E-06 | 4 |
| BP | GO:0048305 | immunoglobulin secretion | 8.37E-07 | 4.21E-06 | 3 |
| BP | GO:1902884 | positive regulation of response to oxidative stress | 8.37E-07 | 4.21E-06 | 3 |
| BP | GO:0002708 | positive regulation of lymphocyte mediated immunity | 8.90E-07 | 4.42E-06 | 4 |
| BP | GO:0002821 | positive regulation of adaptive immune response | 9.35E-07 | 4.60E-06 | 4 |
| BP | GO:0071216 | cellular response to biotic stimulus | 1.08E-06 | 5.27E-06 | 5 |
| BP | GO:0002724 | regulation of T cell cytokine production | 1.30E-06 | 6.18E-06 | 3 |
| BP | GO:2001267 | regulation of cysteine-type endopeptidase activity involved in apoptotic signaling pathway | 1.30E-06 | 6.18E-06 | 3 |
| BP | GO:0002697 | regulation of immune effector process | 1.40E-06 | 6.61E-06 | 6 |
| BP | GO:0043281 | regulation of cysteine-type endopeptidase activity involved in apoptotic process | 1.44E-06 | 6.70E-06 | 5 |
| BP | GO:0006919 | activation of cysteine-type endopeptidase activity involved in apoptotic process | 1.49E-06 | 6.89E-06 | 4 |
| BP | GO:0050727 | regulation of inflammatory response | 1.53E-06 | 6.95E-06 | 6 |
| BP | GO:0097191 | extrinsic apoptotic signaling pathway | 1.54E-06 | 6.95E-06 | 5 |
| BP | GO:0002440 | production of molecular mediator of immune response | 1.68E-06 | 7.52E-06 | 5 |
| BP | GO:0042035 | regulation of cytokine biosynthetic process | 1.78E-06 | 7.86E-06 | 4 |
| BP | GO:0002705 | positive regulation of leukocyte mediated immunity | 2.18E-06 | 9.56E-06 | 4 |
| BP | GO:2001237 | negative regulation of extrinsic apoptotic signaling pathway | 2.45E-06 | 1.07E-05 | 4 |
| BP | GO:1903706 | regulation of hemopoiesis | 2.59E-06 | 1.11E-05 | 6 |
| BP | GO:0045637 | regulation of myeloid cell differentiation | 2.62E-06 | 1.11E-05 | 5 |
| BP | GO:0042089 | cytokine biosynthetic process | 2.65E-06 | 1.12E-05 | 4 |
| BP | GO:0032743 | positive regulation of interleukin-2 production | 2.67E-06 | 1.12E-05 | 3 |
| BP | GO:0042107 | cytokine metabolic process | 2.75E-06 | 1.14E-05 | 4 |
| BP | GO:2000116 | regulation of cysteine-type endopeptidase activity | 2.89E-06 | 1.19E-05 | 5 |
| BP | GO:0002711 | positive regulation of T cell mediated immunity | 3.62E-06 | 1.47E-05 | 3 |
| BP | GO:0002761 | regulation of myeloid leukocyte differentiation | 3.68E-06 | 1.48E-05 | 4 |
| BP | GO:0002369 | T cell cytokine production | 4.36E-06 | 1.74E-05 | 3 |
| BP | GO:0002822 | regulation of adaptive immune response based on somatic recombination of immune receptors built from immunoglobulin superfamily domains | 4.51E-06 | 1.79E-05 | 4 |
| BP | GO:0032479 | regulation of type I interferon production | 4.98E-06 | 1.95E-05 | 4 |
| BP | GO:0002706 | regulation of lymphocyte mediated immunity | 5.14E-06 | 1.95E-05 | 4 |
| BP | GO:0032606 | type I interferon production | 5.14E-06 | 1.95E-05 | 4 |
| BP | GO:0050729 | positive regulation of inflammatory response | 5.14E-06 | 1.95E-05 | 4 |
| BP | GO:0051291 | protein heterooligomerization | 5.14E-06 | 1.95E-05 | 4 |
| BP | GO:0034599 | cellular response to oxidative stress | 5.69E-06 | 2.14E-05 | 5 |
| BP | GO:0032103 | positive regulation of response to external stimulus | 5.98E-06 | 2.22E-05 | 5 |
| BP | GO:0043280 | positive regulation of cysteine-type endopeptidase activity involved in apoptotic process | 6.02E-06 | 2.22E-05 | 4 |
| BP | GO:1902042 | negative regulation of extrinsic apoptotic signaling pathway via death domain receptors | 6.13E-06 | 2.25E-05 | 3 |
| BP | GO:0002819 | regulation of adaptive immune response | 6.40E-06 | 2.33E-05 | 4 |
| BP | GO:0061028 | establishment of endothelial barrier | 6.64E-06 | 2.39E-05 | 3 |
| BP | GO:0009615 | response to virus | 8.41E-06 | 3.01E-05 | 5 |
| BP | GO:0010883 | regulation of lipid storage | 8.94E-06 | 3.13E-05 | 3 |
| BP | GO:0045601 | regulation of endothelial cell differentiation | 8.94E-06 | 3.13E-05 | 3 |
| BP | GO:0002460 | adaptive immune response based on somatic recombination of immune receptors built from immunoglobulin superfamily domains | 8.95E-06 | 3.13E-05 | 5 |
| BP | GO:2001056 | positive regulation of cysteine-type endopeptidase activity | 9.31E-06 | 3.23E-05 | 4 |
| BP | GO:0032757 | positive regulation of interleukin-8 production | 1.03E-05 | 3.53E-05 | 3 |
| BP | GO:2001236 | regulation of extrinsic apoptotic signaling pathway | 1.24E-05 | 4.23E-05 | 4 |
| BP | GO:1905953 | negative regulation of lipid localization | 1.25E-05 | 4.23E-05 | 3 |
| BP | GO:0032655 | regulation of interleukin-12 production | 1.41E-05 | 4.72E-05 | 3 |
| BP | GO:0032663 | regulation of interleukin-2 production | 1.41E-05 | 4.72E-05 | 3 |
| BP | GO:0034614 | cellular response to reactive oxygen species | 1.48E-05 | 4.91E-05 | 4 |
| BP | GO:0002763 | positive regulation of myeloid leukocyte differentiation | 1.50E-05 | 4.94E-05 | 3 |
| BP | GO:0032615 | interleukin-12 production | 1.59E-05 | 5.20E-05 | 3 |
| BP | GO:0002709 | regulation of T cell mediated immunity | 1.69E-05 | 5.47E-05 | 3 |
| BP | GO:0001885 | endothelial cell development | 1.78E-05 | 5.74E-05 | 3 |
| BP | GO:0010950 | positive regulation of endopeptidase activity | 1.83E-05 | 5.86E-05 | 4 |
| BP | GO:0002478 | antigen processing and presentation of exogenous peptide antigen | 1.96E-05 | 6.23E-05 | 4 |
| BP | GO:0002637 | regulation of immunoglobulin production | 1.99E-05 | 6.28E-05 | 3 |
| BP | GO:0032623 | interleukin-2 production | 2.10E-05 | 6.53E-05 | 3 |
| BP | GO:0042108 | positive regulation of cytokine biosynthetic process | 2.10E-05 | 6.53E-05 | 3 |
| BP | GO:0019884 | antigen processing and presentation of exogenous antigen | 2.29E-05 | 7.07E-05 | 4 |
| BP | GO:1902041 | regulation of extrinsic apoptotic signaling pathway via death domain receptors | 2.33E-05 | 7.15E-05 | 3 |
| BP | GO:0048002 | antigen processing and presentation of peptide antigen | 2.61E-05 | 7.93E-05 | 4 |
| BP | GO:0010952 | positive regulation of peptidase activity | 2.66E-05 | 8.04E-05 | 4 |
| BP | GO:0030099 | myeloid cell differentiation | 2.68E-05 | 8.04E-05 | 5 |
| BP | GO:0002573 | myeloid leukocyte differentiation | 2.83E-05 | 8.42E-05 | 4 |
| BP | GO:0032677 | regulation of interleukin-8 production | 2.84E-05 | 8.42E-05 | 3 |
| BP | GO:0019915 | lipid storage | 2.98E-05 | 8.77E-05 | 3 |
| BP | GO:0033619 | membrane protein proteolysis | 3.12E-05 | 9.13E-05 | 3 |
| BP | GO:0045670 | regulation of osteoclast differentiation | 3.27E-05 | 9.50E-05 | 3 |
| BP | GO:0052548 | regulation of endopeptidase activity | 3.39E-05 | 9.80E-05 | 5 |
| BP | GO:0050708 | regulation of protein secretion | 3.89E-05 | 0.000111 | 5 |
| BP | GO:0032637 | interleukin-8 production | 3.90E-05 | 0.000111 | 3 |
| BP | GO:1901033 | positive regulation of response to reactive oxygen species | 3.91E-05 | 0.000111 | 2 |
| BP | GO:0009612 | response to mechanical stimulus | 4.03E-05 | 0.000113 | 4 |
| BP | GO:2001234 | negative regulation of apoptotic signaling pathway | 4.34E-05 | 0.000121 | 4 |
| BP | GO:0006979 | response to oxidative stress | 4.35E-05 | 0.000121 | 5 |
| BP | GO:0032481 | positive regulation of type I interferon production | 4.43E-05 | 0.000122 | 3 |
| BP | GO:0052547 | regulation of peptidase activity | 4.59E-05 | 0.000126 | 5 |
| BP | GO:0032755 | positive regulation of interleukin-6 production | 4.61E-05 | 0.000126 | 3 |
| BP | GO:0042368 | vitamin D biosynthetic process | 4.78E-05 | 0.000129 | 2 |
| BP | GO:0002479 | antigen processing and presentation of exogenous peptide antigen via MHC class I, TAP-dependent | 4.80E-05 | 0.000129 | 3 |
| BP | GO:0019882 | antigen processing and presentation | 5.01E-05 | 0.000134 | 4 |
| BP | GO:0002791 | regulation of peptide secretion | 5.33E-05 | 0.000142 | 5 |
| BP | GO:0042590 | antigen processing and presentation of exogenous peptide antigen via MHC class I | 5.40E-05 | 0.000142 | 3 |
| BP | GO:0071260 | cellular response to mechanical stimulus | 5.40E-05 | 0.000142 | 3 |
| BP | GO:0000302 | response to reactive oxygen species | 5.56E-05 | 0.000145 | 4 |
| BP | GO:0030656 | regulation of vitamin metabolic process | 5.73E-05 | 0.000147 | 2 |
| BP | GO:0045414 | regulation of interleukin-8 biosynthetic process | 5.73E-05 | 0.000147 | 2 |
| BP | GO:0051798 | positive regulation of hair follicle development | 5.73E-05 | 0.000147 | 2 |
| BP | GO:0002456 | T cell mediated immunity | 6.04E-05 | 0.000154 | 3 |
| BP | GO:0042228 | interleukin-8 biosynthetic process | 6.77E-05 | 0.00017 | 2 |
| BP | GO:0042635 | positive regulation of hair cycle | 6.77E-05 | 0.00017 | 2 |
| BP | GO:0070431 | nucleotide-binding oligomerization domain containing 2 signaling pathway | 6.77E-05 | 0.00017 | 2 |
| BP | GO:1900407 | regulation of cellular response to oxidative stress | 6.98E-05 | 0.000174 | 3 |
| BP | GO:0001818 | negative regulation of cytokine production | 7.47E-05 | 0.000185 | 4 |
| BP | GO:0045639 | positive regulation of myeloid cell differentiation | 7.48E-05 | 0.000185 | 3 |
| BP | GO:0042362 | fat-soluble vitamin biosynthetic process | 7.89E-05 | 0.000191 | 2 |
| BP | GO:0090153 | regulation of sphingolipid biosynthetic process | 7.89E-05 | 0.000191 | 2 |
| BP | GO:1905038 | regulation of membrane lipid metabolic process | 7.89E-05 | 0.000191 | 2 |
| BP | GO:2000303 | regulation of ceramide biosynthetic process | 7.89E-05 | 0.000191 | 2 |
| BP | GO:0030316 | osteoclast differentiation | 8.55E-05 | 0.000206 | 3 |
| BP | GO:2000379 | positive regulation of reactive oxygen species metabolic process | 8.83E-05 | 0.000211 | 3 |
| BP | GO:1902105 | regulation of leukocyte differentiation | 8.86E-05 | 0.000211 | 4 |
| BP | GO:0002474 | antigen processing and presentation of peptide antigen via MHC class I | 9.42E-05 | 0.000222 | 3 |
| BP | GO:1902882 | regulation of response to oxidative stress | 9.42E-05 | 0.000222 | 3 |
| BP | GO:1903209 | positive regulation of oxidative stress-induced cell death | 0.000104 | 0.000243 | 2 |
| BP | GO:0010888 | negative regulation of lipid storage | 0.000118 | 0.000274 | 2 |
| BP | GO:0045446 | endothelial cell differentiation | 0.000131 | 0.000303 | 3 |
| BP | GO:0000185 | activation of MAPKKK activity | 0.000132 | 0.000305 | 2 |
| BP | GO:0018105 | peptidyl-serine phosphorylation | 0.00014 | 0.00032 | 4 |
| BP | GO:0035994 | response to muscle stretch | 0.000148 | 0.000337 | 2 |
| BP | GO:0032675 | regulation of interleukin-6 production | 0.000154 | 0.00035 | 3 |
| BP | GO:0032727 | positive regulation of interferon-alpha production | 0.000164 | 0.000365 | 2 |
| BP | GO:0051797 | regulation of hair follicle development | 0.000164 | 0.000365 | 2 |
| BP | GO:1901889 | negative regulation of cell junction assembly | 0.000164 | 0.000365 | 2 |
| BP | GO:2000810 | regulation of bicellular tight junction assembly | 0.000164 | 0.000365 | 2 |
| BP | GO:0050715 | positive regulation of cytokine secretion | 0.000176 | 0.000389 | 3 |
| BP | GO:0032635 | interleukin-6 production | 0.000194 | 0.000428 | 3 |
| BP | GO:0002449 | lymphocyte mediated immunity | 0.000197 | 0.000432 | 4 |
| BP | GO:0003158 | endothelium development | 0.000209 | 0.000456 | 3 |
| BP | GO:0018209 | peptidyl-serine modification | 0.000214 | 0.000465 | 4 |
| BP | GO:0009110 | vitamin biosynthetic process | 0.000218 | 0.000469 | 2 |
| BP | GO:0042359 | vitamin D metabolic process | 0.000218 | 0.000469 | 2 |
| BP | GO:0031293 | membrane protein intracellular domain proteolysis | 0.000238 | 0.000507 | 2 |
| BP | GO:0045672 | positive regulation of osteoclast differentiation | 0.000238 | 0.000507 | 2 |
| BP | GO:0046885 | regulation of hormone biosynthetic process | 0.000258 | 0.000548 | 2 |
| BP | GO:1902107 | positive regulation of leukocyte differentiation | 0.000263 | 0.000554 | 3 |
| BP | GO:0035690 | cellular response to drug | 0.000264 | 0.000554 | 4 |
| BP | GO:0032647 | regulation of interferon-alpha production | 0.00028 | 0.000585 | 2 |
| BP | GO:1905952 | regulation of lipid localization | 0.000293 | 0.00061 | 3 |
| BP | GO:0045862 | positive regulation of proteolysis | 0.000295 | 0.00061 | 4 |
| BP | GO:0032607 | interferon-alpha production | 0.000302 | 0.000623 | 2 |
| BP | GO:0042634 | regulation of hair cycle | 0.000325 | 0.000667 | 2 |
| BP | GO:0002377 | immunoglobulin production | 0.000346 | 0.000707 | 3 |
| BP | GO:0010743 | regulation of macrophage derived foam cell differentiation | 0.000349 | 0.00071 | 2 |
| BP | GO:0030856 | regulation of epithelial cell differentiation | 0.00039 | 0.000788 | 3 |
| BP | GO:0034142 | toll-like receptor 4 signaling pathway | 0.000399 | 0.000805 | 2 |
| BP | GO:2001233 | regulation of apoptotic signaling pathway | 0.000415 | 0.000832 | 4 |
| BP | GO:0032735 | positive regulation of interleukin-12 production | 0.000426 | 0.000846 | 2 |
| BP | GO:0043276 | anoikis | 0.000426 | 0.000846 | 2 |
| BP | GO:0032350 | regulation of hormone metabolic process | 0.000453 | 0.000896 | 2 |
| BP | GO:1901099 | negative regulation of signal transduction in absence of ligand | 0.000481 | 0.000944 | 2 |
| BP | GO:2001240 | negative regulation of extrinsic apoptotic signaling pathway in absence of ligand | 0.000481 | 0.000944 | 2 |
| BP | GO:0010742 | macrophage derived foam cell differentiation | 0.00051 | 0.000992 | 2 |
| BP | GO:0090077 | foam cell differentiation | 0.00051 | 0.000992 | 2 |
| BP | GO:0046890 | regulation of lipid biosynthetic process | 0.000513 | 0.000993 | 3 |
| BP | GO:0045684 | positive regulation of epidermis development | 0.000539 | 0.001037 | 2 |
| BP | GO:1903708 | positive regulation of hemopoiesis | 0.00054 | 0.001037 | 3 |
| BP | GO:0050707 | regulation of cytokine secretion | 0.000559 | 0.001069 | 3 |
| BP | GO:2000377 | regulation of reactive oxygen species metabolic process | 0.000569 | 0.00108 | 3 |
| BP | GO:0032715 | negative regulation of interleukin-6 production | 0.00057 | 0.00108 | 2 |
| BP | GO:2001235 | positive regulation of apoptotic signaling pathway | 0.000598 | 0.001128 | 3 |
| BP | GO:0042088 | T-helper 1 type immune response | 0.000633 | 0.001191 | 2 |
| BP | GO:0007179 | transforming growth factor beta receptor signaling pathway | 0.000659 | 0.001233 | 3 |
| BP | GO:0030866 | cortical actin cytoskeleton organization | 0.000666 | 0.001242 | 2 |
| BP | GO:0045746 | negative regulation of Notch signaling pathway | 0.0007 | 0.0013 | 2 |
| BP | GO:0045429 | positive regulation of nitric oxide biosynthetic process | 0.000735 | 0.001353 | 2 |
| BP | GO:1904407 | positive regulation of nitric oxide metabolic process | 0.000735 | 0.001353 | 2 |
| BP | GO:0006775 | fat-soluble vitamin metabolic process | 0.000806 | 0.001478 | 2 |
| BP | GO:0050663 | cytokine secretion | 0.000817 | 0.001492 | 3 |
| BP | GO:0070534 | protein K63-linked ubiquitination | 0.000843 | 0.001534 | 2 |
| BP | GO:0002064 | epithelial cell development | 0.000879 | 0.001577 | 3 |
| BP | GO:0000060 | protein import into nucleus, translocation | 0.000881 | 0.001577 | 2 |
| BP | GO:1901031 | regulation of response to reactive oxygen species | 0.000881 | 0.001577 | 2 |
| BP | GO:2001239 | regulation of extrinsic apoptotic signaling pathway in absence of ligand | 0.000881 | 0.001577 | 2 |
| BP | GO:0035094 | response to nicotine | 0.00092 | 0.001639 | 2 |
| BP | GO:0098586 | cellular response to virus | 0.000959 | 0.001703 | 2 |
| BP | GO:0006914 | autophagy | 0.000985 | 0.001734 | 4 |
| BP | GO:0061919 | process utilizing autophagic mechanism | 0.000985 | 0.001734 | 4 |
| BP | GO:0002521 | leukocyte differentiation | 0.000992 | 0.001741 | 4 |
| BP | GO:0030865 | cortical cytoskeleton organization | 0.001041 | 0.001811 | 2 |
| BP | GO:2001238 | positive regulation of extrinsic apoptotic signaling pathway | 0.001041 | 0.001811 | 2 |
| BP | GO:0032371 | regulation of sterol transport | 0.001082 | 0.001863 | 2 |
| BP | GO:0032374 | regulation of cholesterol transport | 0.001082 | 0.001863 | 2 |
| BP | GO:0070830 | bicellular tight junction assembly | 0.001082 | 0.001863 | 2 |
| BP | GO:0032515 | negative regulation of phosphoprotein phosphatase activity | 0.001125 | 0.001929 | 2 |
| BP | GO:1903428 | positive regulation of reactive oxygen species biosynthetic process | 0.001169 | 0.001995 | 2 |
| BP | GO:0032722 | positive regulation of chemokine production | 0.001258 | 0.002132 | 2 |
| BP | GO:0046513 | ceramide biosynthetic process | 0.001258 | 0.002132 | 2 |
| BP | GO:0071560 | cellular response to transforming growth factor beta stimulus | 0.001281 | 0.002162 | 3 |
| BP | GO:0035308 | negative regulation of protein dephosphorylation | 0.001304 | 0.002193 | 2 |
| BP | GO:0097237 | cellular response to toxic substance | 0.001313 | 0.0022 | 3 |
| BP | GO:0071559 | response to transforming growth factor beta | 0.001346 | 0.002247 | 3 |
| BP | GO:0072659 | protein localization to plasma membrane | 0.001363 | 0.002267 | 3 |
| BP | GO:0032768 | regulation of monooxygenase activity | 0.001398 | 0.002299 | 2 |
| BP | GO:0045428 | regulation of nitric oxide biosynthetic process | 0.001398 | 0.002299 | 2 |
| BP | GO:0051865 | protein autoubiquitination | 0.001398 | 0.002299 | 2 |
| BP | GO:0050714 | positive regulation of protein secretion | 0.001431 | 0.002345 | 3 |
| BP | GO:0034121 | regulation of toll-like receptor signaling pathway | 0.001495 | 0.002432 | 2 |
| BP | GO:0043297 | apical junction assembly | 0.001495 | 0.002432 | 2 |
| BP | GO:0016239 | positive regulation of macroautophagy | 0.001595 | 0.002577 | 2 |
| BP | GO:0032729 | positive regulation of interferon-gamma production | 0.001595 | 0.002577 | 2 |
| BP | GO:0046889 | positive regulation of lipid biosynthetic process | 0.001699 | 0.002724 | 2 |
| BP | GO:1902930 | regulation of alcohol biosynthetic process | 0.001699 | 0.002724 | 2 |
| BP | GO:0072593 | reactive oxygen species metabolic process | 0.001763 | 0.002817 | 3 |
| BP | GO:0002793 | positive regulation of peptide secretion | 0.001823 | 0.002902 | 3 |
| BP | GO:0032760 | positive regulation of tumor necrosis factor production | 0.00186 | 0.002951 | 2 |
| BP | GO:1990778 | protein localization to cell periphery | 0.001967 | 0.003106 | 3 |
| BP | GO:0006809 | nitric oxide biosynthetic process | 0.001972 | 0.003106 | 2 |
| BP | GO:0038034 | signal transduction in absence of ligand | 0.002028 | 0.003162 | 2 |
| BP | GO:0097192 | extrinsic apoptotic signaling pathway in absence of ligand | 0.002028 | 0.003162 | 2 |
| BP | GO:1903557 | positive regulation of tumor necrosis factor superfamily cytokine production | 0.002028 | 0.003162 | 2 |
| BP | GO:0046209 | nitric oxide metabolic process | 0.002204 | 0.003423 | 2 |
| BP | GO:0032642 | regulation of chemokine production | 0.002263 | 0.003504 | 2 |
| BP | GO:1901214 | regulation of neuron death | 0.0023 | 0.003548 | 3 |
| BP | GO:1903201 | regulation of oxidative stress-induced cell death | 0.002324 | 0.003561 | 2 |
| BP | GO:1905954 | positive regulation of lipid localization | 0.002324 | 0.003561 | 2 |
| BP | GO:0000271 | polysaccharide biosynthetic process | 0.002386 | 0.00363 | 2 |
| BP | GO:2001057 | reactive nitrogen species metabolic process | 0.002386 | 0.00363 | 2 |
| BP | GO:0097193 | intrinsic apoptotic signaling pathway | 0.002515 | 0.003814 | 3 |
| CC | GO:0008385 | IkappaB kinase complex | 1.54E-10 | 2.10E-09 | 4 |
| CC | GO:0035631 | CD40 receptor complex | 1.54E-10 | 2.10E-09 | 4 |
| CC | GO:0043235 | receptor complex | 4.94E-10 | 4.51E-09 | 8 |
| CC | GO:0045121 | membrane raft | 4.53E-09 | 2.54E-08 | 7 |
| CC | GO:0098857 | membrane microdomain | 4.63E-09 | 2.54E-08 | 7 |
| CC | GO:0098589 | membrane region | 5.93E-09 | 2.71E-08 | 7 |
| CC | GO:0010008 | endosome membrane | 7.16E-08 | 2.80E-07 | 7 |
| CC | GO:0044440 | endosomal part | 1.37E-07 | 4.68E-07 | 7 |
| CC | GO:1902554 | serine/threonine protein kinase complex | 9.05E-07 | 2.75E-06 | 4 |
| CC | GO:1902911 | protein kinase complex | 1.67E-06 | 4.57E-06 | 4 |
| CC | GO:0044445 | cytosolic part | 2.46E-06 | 6.11E-06 | 5 |
| CC | GO:0009898 | cytoplasmic side of plasma membrane | 1.64E-05 | 3.69E-05 | 4 |
| CC | GO:0098802 | plasma membrane receptor complex | 1.75E-05 | 3.69E-05 | 4 |
| CC | GO:0098562 | cytoplasmic side of membrane | 2.66E-05 | 5.20E-05 | 4 |
| CC | GO:0061695 | transferase complex, transferring phosphorus-containing groups | 9.63E-05 | 0.000176 | 4 |
| MF | GO:0032813 | tumor necrosis factor receptor superfamily binding | 5.98E-10 | 2.33E-08 | 5 |
| MF | GO:0005164 | tumor necrosis factor receptor binding | 1.87E-08 | 3.63E-07 | 4 |
| MF | GO:0031625 | ubiquitin protein ligase binding | 2.99E-07 | 3.77E-06 | 6 |
| MF | GO:0044389 | ubiquitin-like protein ligase binding | 3.87E-07 | 3.77E-06 | 6 |
| MF | GO:0005126 | cytokine receptor binding | 5.10E-06 | 3.97E-05 | 5 |
| MF | GO:0097110 | scaffold protein binding | 2.37E-05 | 0.000154 | 3 |
| MF | GO:0070513 | death domain binding | 3.96E-05 | 0.00022 | 2 |
| MF | GO:0031996 | thioesterase binding | 4.83E-05 | 0.000235 | 2 |
| MF | GO:0046982 | protein heterodimerization activity | 8.58E-05 | 0.000371 | 5 |
| MF | GO:0031435 | mitogen-activated protein kinase kinase kinase binding | 0.000105 | 0.00041 | 2 |
| MF | GO:0070530 | K63-linked polyubiquitin modification-dependent protein binding | 0.000184 | 0.00065 | 2 |
| MF | GO:0031593 | polyubiquitin modification-dependent protein binding | 0.000853 | 0.00277 | 2 |

Module 2:

| ONTOLOGY | ID | Description | pvalue | qvalue | Count |
| --- | --- | --- | --- | --- | --- |
| BP | GO:0070098 | chemokine-mediated signaling pathway | 2.15E-09 | 3.65E-07 | 4 |
| BP | GO:0006874 | cellular calcium ion homeostasis | 7.45E-09 | 4.68E-07 | 5 |
| BP | GO:0055074 | calcium ion homeostasis | 8.48E-09 | 4.68E-07 | 5 |
| BP | GO:0072503 | cellular divalent inorganic cation homeostasis | 1.14E-08 | 4.68E-07 | 5 |
| BP | GO:0072507 | divalent inorganic cation homeostasis | 1.38E-08 | 4.68E-07 | 5 |
| BP | GO:0002407 | dendritic cell chemotaxis | 1.93E-08 | 5.45E-07 | 3 |
| BP | GO:0036336 | dendritic cell migration | 3.18E-08 | 7.71E-07 | 3 |
| BP | GO:0030595 | leukocyte chemotaxis | 8.25E-08 | 1.75E-06 | 4 |
| BP | GO:2000107 | negative regulation of leukocyte apoptotic process | 2.00E-07 | 3.77E-06 | 3 |
| BP | GO:0060326 | cell chemotaxis | 2.76E-07 | 4.68E-06 | 4 |
| BP | GO:2000106 | regulation of leukocyte apoptotic process | 1.03E-06 | 1.59E-05 | 3 |
| BP | GO:0006816 | calcium ion transport | 1.45E-06 | 2.05E-05 | 4 |
| BP | GO:0071887 | leukocyte apoptotic process | 1.86E-06 | 2.42E-05 | 3 |
| BP | GO:0070838 | divalent metal ion transport | 2.19E-06 | 2.56E-05 | 4 |
| BP | GO:0072511 | divalent inorganic cation transport | 2.27E-06 | 2.56E-05 | 4 |
| BP | GO:0050900 | leukocyte migration | 2.43E-06 | 2.57E-05 | 4 |
| BP | GO:2000109 | regulation of macrophage apoptotic process | 2.89E-06 | 2.88E-05 | 2 |
| BP | GO:0071888 | macrophage apoptotic process | 4.23E-06 | 3.98E-05 | 2 |
| BP | GO:0042976 | activation of Janus kinase activity | 5.00E-06 | 4.46E-05 | 2 |
| BP | GO:0033033 | negative regulation of myeloid cell apoptotic process | 7.69E-06 | 6.52E-05 | 2 |
| BP | GO:0090026 | positive regulation of monocyte chemotaxis | 8.71E-06 | 7.03E-05 | 2 |
| BP | GO:0006925 | inflammatory cell apoptotic process | 1.22E-05 | 9.37E-05 | 2 |
| BP | GO:0071677 | positive regulation of mononuclear cell migration | 1.34E-05 | 9.91E-05 | 2 |
| BP | GO:0050920 | regulation of chemotaxis | 1.45E-05 | 0.0001 | 3 |
| BP | GO:0090025 | regulation of monocyte chemotaxis | 1.48E-05 | 0.0001 | 2 |
| BP | GO:2000406 | positive regulation of T cell migration | 1.92E-05 | 0.000125 | 2 |
| BP | GO:0051924 | regulation of calcium ion transport | 2.14E-05 | 0.000134 | 3 |
| BP | GO:2001025 | positive regulation of response to drug | 2.25E-05 | 0.000136 | 2 |
| BP | GO:0033032 | regulation of myeloid cell apoptotic process | 2.42E-05 | 0.000137 | 2 |
| BP | GO:0043270 | positive regulation of ion transport | 2.43E-05 | 0.000137 | 3 |
| BP | GO:0051954 | positive regulation of amine transport | 2.97E-05 | 0.000158 | 2 |
| BP | GO:2000403 | positive regulation of lymphocyte migration | 2.97E-05 | 0.000158 | 2 |
| BP | GO:0033028 | myeloid cell apoptotic process | 3.17E-05 | 0.000163 | 2 |
| BP | GO:2000404 | regulation of T cell migration | 3.38E-05 | 0.000168 | 2 |
| BP | GO:0032103 | positive regulation of response to external stimulus | 4.24E-05 | 0.000205 | 3 |
| BP | GO:0071675 | regulation of mononuclear cell migration | 4.49E-05 | 0.000206 | 2 |
| BP | GO:0007204 | positive regulation of cytosolic calcium ion concentration | 4.51E-05 | 0.000206 | 3 |
| BP | GO:0014911 | positive regulation of smooth muscle cell migration | 4.98E-05 | 0.000222 | 2 |
| BP | GO:0009615 | response to virus | 5.23E-05 | 0.000227 | 3 |
| BP | GO:0019058 | viral life cycle | 5.53E-05 | 0.000234 | 3 |
| BP | GO:0051480 | regulation of cytosolic calcium ion concentration | 6.02E-05 | 0.000249 | 3 |
| BP | GO:0032147 | activation of protein kinase activity | 6.18E-05 | 0.000249 | 3 |
| BP | GO:0044764 | multi-organism cellular process | 6.32E-05 | 0.000249 | 2 |
| BP | GO:0061098 | positive regulation of protein tyrosine kinase activity | 6.61E-05 | 0.000255 | 2 |
| BP | GO:0019932 | second-messenger-mediated signaling | 7.00E-05 | 0.000264 | 3 |
| BP | GO:0050433 | regulation of catecholamine secretion | 7.20E-05 | 0.000265 | 2 |
| BP | GO:0010518 | positive regulation of phospholipase activity | 7.51E-05 | 0.000265 | 2 |
| BP | GO:2000401 | regulation of lymphocyte migration | 7.51E-05 | 0.000265 | 2 |
| BP | GO:0050432 | catecholamine secretion | 8.14E-05 | 0.000276 | 2 |
| BP | GO:0072678 | T cell migration | 8.14E-05 | 0.000276 | 2 |
| BP | GO:0010959 | regulation of metal ion transport | 8.36E-05 | 0.000278 | 3 |
| BP | GO:0010517 | regulation of phospholipase activity | 0.000105 | 0.000344 | 2 |
| BP | GO:0050918 | positive chemotaxis | 0.000109 | 0.000349 | 2 |
| BP | GO:0002548 | monocyte chemotaxis | 0.000121 | 0.000371 | 2 |
| BP | GO:0060193 | positive regulation of lipase activity | 0.000121 | 0.000371 | 2 |
| BP | GO:0051937 | catecholamine transport | 0.000128 | 0.000389 | 2 |
| BP | GO:0050769 | positive regulation of neurogenesis | 0.000138 | 0.000411 | 3 |
| BP | GO:0014068 | positive regulation of phosphatidylinositol 3-kinase signaling | 0.000149 | 0.000437 | 2 |
| BP | GO:0014910 | regulation of smooth muscle cell migration | 0.000172 | 0.000494 | 2 |
| BP | GO:0015844 | monoamine transport | 0.000177 | 0.000499 | 2 |
| BP | GO:0051952 | regulation of amine transport | 0.000186 | 0.000509 | 2 |
| BP | GO:0061097 | regulation of protein tyrosine kinase activity | 0.000186 | 0.000509 | 2 |
| BP | GO:0071674 | mononuclear cell migration | 0.000196 | 0.000524 | 2 |
| BP | GO:0030335 | positive regulation of cell migration | 0.000199 | 0.000524 | 3 |
| BP | GO:0046427 | positive regulation of JAK-STAT cascade | 0.000201 | 0.000524 | 2 |
| BP | GO:0051962 | positive regulation of nervous system development | 0.000208 | 0.000526 | 3 |
| BP | GO:0014909 | smooth muscle cell migration | 0.000211 | 0.000526 | 2 |
| BP | GO:1904894 | positive regulation of STAT cascade | 0.000211 | 0.000526 | 2 |
| BP | GO:0015837 | amine transport | 0.000222 | 0.000537 | 2 |
| BP | GO:0060191 | regulation of lipase activity | 0.000222 | 0.000537 | 2 |
| BP | GO:0048661 | positive regulation of smooth muscle cell proliferation | 0.000232 | 0.000555 | 2 |
| BP | GO:0002690 | positive regulation of leukocyte chemotaxis | 0.000238 | 0.00056 | 2 |
| BP | GO:2001023 | regulation of response to drug | 0.00026 | 0.000604 | 2 |
| BP | GO:0014812 | muscle cell migration | 0.000272 | 0.000622 | 2 |
| BP | GO:0072676 | lymphocyte migration | 0.000278 | 0.000627 | 2 |
| BP | GO:0014066 | regulation of phosphatidylinositol 3-kinase signaling | 0.000321 | 0.000715 | 2 |
| BP | GO:0051928 | positive regulation of calcium ion transport | 0.000366 | 0.000806 | 2 |
| BP | GO:0002688 | regulation of leukocyte chemotaxis | 0.000387 | 0.000841 | 2 |
| BP | GO:0046718 | viral entry into host cell | 0.000445 | 0.000954 | 2 |
| BP | GO:0002687 | positive regulation of leukocyte migration | 0.000475 | 0.001006 | 2 |
| BP | GO:0014065 | phosphatidylinositol 3-kinase signaling | 0.00053 | 0.001096 | 2 |
| BP | GO:0050921 | positive regulation of chemotaxis | 0.00053 | 0.001096 | 2 |
| BP | GO:0030260 | entry into host cell | 0.000572 | 0.001127 | 2 |
| BP | GO:0044409 | entry into host | 0.000572 | 0.001127 | 2 |
| BP | GO:0051806 | entry into cell of other organism involved in symbiotic interaction | 0.000572 | 0.001127 | 2 |
| BP | GO:0051828 | entry into other organism involved in symbiotic interaction | 0.000572 | 0.001127 | 2 |
| BP | GO:0048660 | regulation of smooth muscle cell proliferation | 0.000641 | 0.001249 | 2 |
| BP | GO:0048659 | smooth muscle cell proliferation | 0.000668 | 0.001287 | 2 |
| BP | GO:0046425 | regulation of JAK-STAT cascade | 0.000743 | 0.001415 | 2 |
| BP | GO:1904892 | regulation of STAT cascade | 0.000763 | 0.001436 | 2 |
| BP | GO:0048015 | phosphatidylinositol-mediated signaling | 0.000782 | 0.001457 | 2 |
| BP | GO:0048017 | inositol lipid-mediated signaling | 0.000812 | 0.001496 | 2 |
| BP | GO:0019722 | calcium-mediated signaling | 0.000915 | 0.001668 | 2 |
| BP | GO:0007259 | JAK-STAT cascade | 0.000947 | 0.00169 | 2 |
| BP | GO:0071222 | cellular response to lipopolysaccharide | 0.000947 | 0.00169 | 2 |
| BP | GO:0002685 | regulation of leukocyte migration | 0.000969 | 0.001693 | 2 |
| BP | GO:0097696 | STAT cascade | 0.000969 | 0.001693 | 2 |
| BP | GO:0097529 | myeloid leukocyte migration | 0.00098 | 0.001695 | 2 |
| BP | GO:0050731 | positive regulation of peptidyl-tyrosine phosphorylation | 0.001002 | 0.001716 | 2 |
| BP | GO:0071219 | cellular response to molecule of bacterial origin | 0.001036 | 0.001755 | 2 |
| BP | GO:0050679 | positive regulation of epithelial cell proliferation | 0.001152 | 0.001933 | 2 |
| BP | GO:0015893 | drug transport | 0.001188 | 0.001973 | 2 |
| BP | GO:0071216 | cellular response to biotic stimulus | 0.001299 | 0.002137 | 2 |
| BP | GO:0009612 | response to mechanical stimulus | 0.001363 | 0.002199 | 2 |
| BP | GO:0033002 | muscle cell proliferation | 0.001389 | 0.002199 | 2 |
| BP | GO:0048588 | developmental cell growth | 0.001389 | 0.002199 | 2 |
| BP | GO:0051701 | interaction with host | 0.001389 | 0.002199 | 2 |
| MF | GO:0019957 | C-C chemokine binding | 2.92E-06 | 2.46E-05 | 2 |
| MF | GO:0019956 | chemokine binding | 1.94E-05 | 3.55E-05 | 2 |
| MF | GO:0001637 | G-protein coupled chemoattractant receptor activity | 2.11E-05 | 3.55E-05 | 2 |
| MF | GO:0004435 | phosphatidylinositol phospholipase C activity | 2.11E-05 | 3.55E-05 | 2 |
| MF | GO:0004950 | chemokine receptor activity | 2.11E-05 | 3.55E-05 | 2 |
| MF | GO:0004629 | phospholipase C activity | 2.63E-05 | 3.69E-05 | 2 |
| MF | GO:0001664 | G-protein coupled receptor binding | 3.56E-05 | 3.82E-05 | 3 |
| MF | GO:0042056 | chemoattractant activity | 3.63E-05 | 3.82E-05 | 2 |
| MF | GO:0015026 | coreceptor activity | 5.57E-05 | 5.21E-05 | 2 |
| MF | GO:0008009 | chemokine activity | 7.60E-05 | 6.40E-05 | 2 |
| MF | GO:0042379 | chemokine receptor binding | 0.000138 | 0.000106 | 2 |
| MF | GO:0001618 | virus receptor activity | 0.000174 | 0.000112 | 2 |
| MF | GO:0104005 | hijacked molecular function | 0.000174 | 0.000112 | 2 |
| MF | GO:0048018 | receptor ligand activity | 0.000186 | 0.000112 | 3 |
| MF | GO:0008081 | phosphoric diester hydrolase activity | 0.000263 | 0.000145 | 2 |
| MF | GO:0004896 | cytokine receptor activity | 0.000275 | 0.000145 | 2 |
| MF | GO:0004620 | phospholipase activity | 0.000312 | 0.000154 | 2 |
| MF | GO:0019955 | cytokine binding | 0.000337 | 0.000158 | 2 |
| MF | GO:0016298 | lipase activity | 0.000529 | 0.000229 | 2 |
| MF | GO:0008528 | G-protein coupled peptide receptor activity | 0.000562 | 0.000229 | 2 |
| MF | GO:0001653 | peptide receptor activity | 0.00057 | 0.000229 | 2 |
| MF | GO:0008083 | growth factor activity | 0.000842 | 0.000322 | 2 |
| MF | GO:0005125 | cytokine activity | 0.001526 | 0.000559 | 2 |
| MF | GO:0005126 | cytokine receptor binding | 0.002423 | 0.00085 | 2 |
| MF | GO:0031702 | type 1 angiotensin receptor binding | 0.002846 | 0.000959 | 1 |
| MF | GO:0016004 | phospholipase activator activity | 0.003131 | 0.000976 | 1 |
| MF | GO:0031701 | angiotensin receptor binding | 0.003131 | 0.000976 | 1 |
| MF | GO:0016493 | C-C chemokine receptor activity | 0.003415 | 0.001027 | 1 |
| MF | GO:0060229 | lipase activator activity | 0.003699 | 0.001074 | 1 |
| MF | GO:0042578 | phosphoric ester hydrolase activity | 0.004117 | 0.001156 | 2 |
| MF | GO:0045236 | CXCR chemokine receptor binding | 0.004835 | 0.001272 | 1 |
| MF | GO:0048019 | receptor antagonist activity | 0.004835 | 0.001272 | 1 |
| MF | GO:0003779 | actin binding | 0.005197 | 0.001326 | 2 |
| MF | GO:0030296 | protein tyrosine kinase activator activity | 0.005403 | 0.001338 | 1 |

Module 3:

| ONTOLOGY | ID | Description | pvalue | qvalue | Count |
| --- | --- | --- | --- | --- | --- |
| BP | GO:0030195 | negative regulation of blood coagulation | 3.58E-14 | 2.75E-12 | 6 |
| BP | GO:1900047 | negative regulation of hemostasis | 3.58E-14 | 2.75E-12 | 6 |
| BP | GO:0050819 | negative regulation of coagulation | 6.45E-14 | 3.30E-12 | 6 |
| BP | GO:0007596 | blood coagulation | 1.58E-13 | 4.66E-12 | 8 |
| BP | GO:0007599 | hemostasis | 1.78E-13 | 4.66E-12 | 8 |
| BP | GO:0050817 | coagulation | 1.82E-13 | 4.66E-12 | 8 |
| BP | GO:0061045 | negative regulation of wound healing | 3.10E-13 | 6.80E-12 | 6 |
| BP | GO:0030193 | regulation of blood coagulation | 5.08E-13 | 8.68E-12 | 6 |
| BP | GO:1900046 | regulation of hemostasis | 5.08E-13 | 8.68E-12 | 6 |
| BP | GO:0042730 | fibrinolysis | 7.09E-13 | 1.09E-11 | 5 |
| BP | GO:0050818 | regulation of coagulation | 8.04E-13 | 1.11E-11 | 6 |
| BP | GO:1903035 | negative regulation of response to wounding | 8.65E-13 | 1.11E-11 | 6 |
| BP | GO:0050878 | regulation of body fluid levels | 3.39E-12 | 4.00E-11 | 8 |
| BP | GO:0061041 | regulation of wound healing | 1.41E-11 | 1.55E-10 | 6 |
| BP | GO:0032102 | negative regulation of response to external stimulus | 1.68E-11 | 1.72E-10 | 7 |
| BP | GO:1903034 | regulation of response to wounding | 4.30E-11 | 4.13E-10 | 6 |
| BP | GO:0045861 | negative regulation of proteolysis | 4.81E-09 | 4.35E-08 | 6 |
| BP | GO:0090303 | positive regulation of wound healing | 9.74E-09 | 8.32E-08 | 4 |
| BP | GO:0051918 | negative regulation of fibrinolysis | 1.10E-08 | 8.88E-08 | 3 |
| BP | GO:1903036 | positive regulation of response to wounding | 1.95E-08 | 1.50E-07 | 4 |
| BP | GO:0051917 | regulation of fibrinolysis | 3.33E-08 | 2.43E-07 | 3 |
| BP | GO:0010951 | negative regulation of endopeptidase activity | 6.72E-08 | 4.69E-07 | 5 |
| BP | GO:0010466 | negative regulation of peptidase activity | 8.64E-08 | 5.77E-07 | 5 |
| BP | GO:0030194 | positive regulation of blood coagulation | 2.37E-07 | 1.46E-06 | 3 |
| BP | GO:1900048 | positive regulation of hemostasis | 2.37E-07 | 1.46E-06 | 3 |
| BP | GO:0016485 | protein processing | 2.59E-07 | 1.52E-06 | 5 |
| BP | GO:0050820 | positive regulation of coagulation | 2.66E-07 | 1.52E-06 | 3 |
| BP | GO:0051604 | protein maturation | 6.01E-07 | 3.30E-06 | 5 |
| BP | GO:0052548 | regulation of endopeptidase activity | 8.08E-07 | 4.28E-06 | 5 |
| BP | GO:0052547 | regulation of peptidase activity | 1.10E-06 | 5.65E-06 | 5 |
| BP | GO:0051346 | negative regulation of hydrolase activity | 1.30E-06 | 6.45E-06 | 5 |
| BP | GO:0010810 | regulation of cell-substrate adhesion | 1.60E-06 | 7.70E-06 | 4 |
| BP | GO:0033627 | cell adhesion mediated by integrin | 2.51E-06 | 1.17E-05 | 3 |
| BP | GO:0014910 | regulation of smooth muscle cell migration | 5.83E-06 | 2.64E-05 | 3 |
| BP | GO:0014909 | smooth muscle cell migration | 7.95E-06 | 3.49E-05 | 3 |
| BP | GO:0014812 | muscle cell migration | 1.16E-05 | 4.96E-05 | 3 |
| BP | GO:0031589 | cell-substrate adhesion | 1.23E-05 | 5.00E-05 | 4 |
| BP | GO:0017187 | peptidyl-glutamic acid carboxylation | 1.27E-05 | 5.00E-05 | 2 |
| BP | GO:0018214 | protein carboxylation | 1.27E-05 | 5.00E-05 | 2 |
| BP | GO:0050727 | regulation of inflammatory response | 3.33E-05 | 0.000128 | 4 |
| BP | GO:0007597 | blood coagulation, intrinsic pathway | 3.93E-05 | 0.000147 | 2 |
| BP | GO:0030335 | positive regulation of cell migration | 6.41E-05 | 0.000234 | 4 |
| BP | GO:0070613 | regulation of protein processing | 6.70E-05 | 0.000237 | 3 |
| BP | GO:0006465 | signal peptide processing | 6.89E-05 | 0.000237 | 2 |
| BP | GO:1903317 | regulation of protein maturation | 6.94E-05 | 0.000237 | 3 |
| BP | GO:0002446 | neutrophil mediated immunity | 7.16E-05 | 0.000239 | 4 |
| BP | GO:0031639 | plasminogen activation | 7.46E-05 | 0.000244 | 2 |
| BP | GO:0072378 | blood coagulation, fibrin clot formation | 8.06E-05 | 0.000258 | 2 |
| BP | GO:0072376 | protein activation cascade | 8.65E-05 | 0.000271 | 3 |
| BP | GO:0018200 | peptidyl-glutamic acid modification | 0.000129 | 0.000393 | 2 |
| BP | GO:0007160 | cell-matrix adhesion | 0.00013 | 0.000393 | 3 |
| BP | GO:0033628 | regulation of cell adhesion mediated by integrin | 0.000178 | 0.000527 | 2 |
| BP | GO:0048260 | positive regulation of receptor-mediated endocytosis | 0.000257 | 0.000747 | 2 |
| BP | GO:0031638 | zymogen activation | 0.000291 | 0.000828 | 2 |
| BP | GO:0032103 | positive regulation of response to external stimulus | 0.000339 | 0.000947 | 3 |
| BP | GO:0002532 | production of molecular mediator involved in inflammatory response | 0.000363 | 0.000997 | 2 |
| BP | GO:0010812 | negative regulation of cell-substrate adhesion | 0.000403 | 0.001086 | 2 |
| BP | GO:0030198 | extracellular matrix organization | 0.00055 | 0.001459 | 3 |
| BP | GO:0051851 | modification by host of symbiont morphology or physiology | 0.000564 | 0.001469 | 2 |
| BP | GO:0051702 | interaction with symbiont | 0.000629 | 0.001611 | 2 |
| BP | GO:0048259 | regulation of receptor-mediated endocytosis | 0.00068 | 0.001713 | 2 |
| BP | GO:0050829 | defense response to Gram-negative bacterium | 0.000788 | 0.001954 | 2 |
| BP | GO:0043062 | extracellular structure organization | 0.000845 | 0.002061 | 3 |
| BP | GO:0043154 | negative regulation of cysteine-type endopeptidase activity involved in apoptotic process | 0.000924 | 0.002219 | 2 |
| BP | GO:0001952 | regulation of cell-matrix adhesion | 0.001251 | 0.002923 | 2 |
| BP | GO:0030449 | regulation of complement activation | 0.001274 | 0.002923 | 2 |
| BP | GO:0051817 | modification of morphology or physiology of other organism involved in symbiotic interaction | 0.001274 | 0.002923 | 2 |
| BP | GO:2000257 | regulation of protein activation cascade | 0.001298 | 0.002934 | 2 |
| BP | GO:2000117 | negative regulation of cysteine-type endopeptidase activity | 0.001371 | 0.003053 | 2 |
| BP | GO:0043312 | neutrophil degranulation | 0.00153 | 0.003359 | 3 |
| BP | GO:0002283 | neutrophil activation involved in immune response | 0.001557 | 0.003371 | 3 |
| BP | GO:0042119 | neutrophil activation | 0.001651 | 0.003524 | 3 |
| BP | GO:0002920 | regulation of humoral immune response | 0.001733 | 0.003649 | 2 |
| BP | GO:0002576 | platelet degranulation | 0.001816 | 0.003772 | 2 |
| BP | GO:0050728 | negative regulation of inflammatory response | 0.001873 | 0.003838 | 2 |
| BP | GO:0045807 | positive regulation of endocytosis | 0.001901 | 0.003845 | 2 |
| CC | GO:0005788 | endoplasmic reticulum lumen | 1.17E-07 | 1.35E-06 | 5 |
| CC | GO:0035579 | specific granule membrane | 9.17E-06 | 5.14E-05 | 3 |
| CC | GO:0005796 | Golgi lumen | 1.33E-05 | 5.14E-05 | 3 |
| CC | GO:0042581 | specific granule | 4.97E-05 | 0.000144 | 3 |
| CC | GO:0072562 | blood microparticle | 7.42E-05 | 0.000172 | 3 |
| CC | GO:0009897 | external side of plasma membrane | 0.000281 | 0.000542 | 3 |
| CC | GO:0030667 | secretory granule membrane | 0.000329 | 0.000545 | 3 |
| CC | GO:0031093 | platelet alpha granule lumen | 0.000448 | 0.000648 | 2 |
| CC | GO:0031091 | platelet alpha granule | 0.000807 | 0.001038 | 2 |
| MF | GO:0004252 | serine-type endopeptidase activity | 4.64E-06 | 2.67E-05 | 4 |
| MF | GO:0008236 | serine-type peptidase activity | 7.09E-06 | 2.67E-05 | 4 |
| MF | GO:0017171 | serine hydrolase activity | 7.61E-06 | 2.67E-05 | 4 |
| MF | GO:0008201 | heparin binding | 5.78E-05 | 0.000125 | 3 |
| MF | GO:0004175 | endopeptidase activity | 5.95E-05 | 0.000125 | 4 |
| MF | GO:0005539 | glycosaminoglycan binding | 0.000144 | 0.000253 | 3 |
| MF | GO:1901681 | sulfur compound binding | 0.000188 | 0.000282 | 3 |
| MF | GO:0004867 | serine-type endopeptidase inhibitor activity | 0.001019 | 0.00134 | 2 |
