# Supplementary material for: Investigating the multi-target pharmacological mechanism of danhong injection acting on unstable angina by combined network pharmacology and molecular docking
Source: BMC Complement Med Ther. 2020 Mar 2;20:66. doi: 10.1186/s12906-020-2853-5 (PMC7076845; doi:10.1186/s12906-020-2853-5)
Supplement: Supplementary file 4 — Additional file 4 Supplementary 4. Table S4. The information of KEGG pathway analysis of DHI-UA PPI network. [file 12906_2020_2853_MOESM4_ESM.docx]

Table S4. The information of KEGG pathway analysis of DHI-UA PPI network

| ID | Description | pvalue | qvalue | geneID | Count |
| --- | --- | --- | --- | --- | --- |
| hsa04064 | NF-kappa B signaling pathway | 1.22E-24 | 1.14E-22 | 7124/7099/8737/7189/8517/330/6885/23118/10454/4790/3551/1147/7186/7132/4792/23643/8717/5328/6387/5743/142 | 21 |
| hsa04668 | TNF signaling pathway | 3.93E-22 | 1.84E-20 | 7124/8737/8517/330/6885/23118/10454/4790/3551/1147/7186/7132/4792/8717/841/5608/7133/6352/6401/5743 | 20 |
| hsa04610 | Complement and coagulation cascades | 9.72E-20 | 3.03E-18 | 2147/2243/5054/5340/2266/2244/2152/462/7056/2159/5328/5329/2155/5327/3053/2158/7448 | 17 |
| hsa05145 | Toxoplasmosis | 4.62E-19 | 1.08E-17 | 7124/7099/7189/8517/330/6885/23118/10454/4790/3551/1147/7132/4792/23643/841/5608/1234/4843 | 18 |
| hsa04620 | Toll-like receptor signaling pathway | 3.74E-18 | 7.00E-17 | 7124/7099/8737/7189/8517/6885/23118/10454/4790/3551/1147/4792/23643/7098/841/5608/6352 | 17 |
| hsa05170 | Human immunodeficiency virus 1 infection | 1.88E-16 | 2.94E-15 | 7124/7099/8737/7189/8517/6885/23118/10454/4790/3551/1147/7186/7132/4792/8717/841/5608/7133/7852/1234 | 20 |
| hsa04657 | IL-17 signaling pathway | 1.82E-14 | 2.43E-13 | 7124/7189/8517/6885/23118/4790/3551/1147/7186/4792/8717/841/5743/4312 | 14 |
| hsa05142 | Chagas disease (American trypanosomiasis) | 5.90E-14 | 6.91E-13 | 7124/7099/7189/8517/4790/3551/1147/7132/4792/841/5054/6352/4843/1636 | 14 |
| hsa04622 | RIG-I-like receptor signaling pathway | 2.77E-13 | 2.88E-12 | 7124/8737/7189/8517/6885/4790/3551/1147/7186/4792/8717/841 | 12 |
| hsa05130 | Pathogenic Escherichia coli infection | 3.32E-13 | 3.11E-12 | 7124/7099/8737/7189/8517/6885/23118/10454/4790/3551/1147/7186/2147/7132/4792/8717/841 | 17 |
| hsa04621 | NOD-like receptor signaling pathway | 8.57E-13 | 7.29E-12 | 7124/7099/8737/7189/8517/330/6885/23118/10454/4790/3551/1147/7186/4792/841/6352 | 16 |
| hsa05163 | Human cytomegalovirus infection | 1.95E-12 | 1.52E-11 | 7124/8737/8517/4790/3551/1147/7186/7132/4792/8717/841/5608/6352/7852/6387/1234/5743 | 17 |
| hsa05135 | Yersinia infection | 1.15E-11 | 8.26E-11 | 7124/7099/7189/8517/6885/23118/10454/4790/3551/1147/7186/4792/5608 | 13 |
| hsa04380 | Osteoclast differentiation | 2.63E-11 | 1.76E-10 | 7124/7189/8517/6885/23118/10454/4790/3551/1147/7186/7132/4792/5608 | 13 |
| hsa05161 | Hepatitis B | 3.77E-11 | 2.36E-10 | 7124/7099/7189/8517/6885/23118/10454/4790/3551/1147/4792/7098/841/5608 | 14 |
| hsa05131 | Shigellosis | 5.04E-11 | 2.95E-10 | 7124/7099/8737/7189/8517/6885/23118/10454/4790/3551/1147/7186/7132/4792/8717/6352 | 16 |
| hsa05169 | Epstein-Barr virus infection | 5.69E-11 | 2.98E-10 | 7124/8737/7189/8517/6885/23118/10454/4790/3551/1147/7186/4792/8717/841/5608 | 15 |
| hsa04210 | Apoptosis | 5.73E-11 | 2.98E-10 | 7124/8737/8517/330/4790/3551/1147/7186/7132/4792/8717/841/142 | 13 |
| hsa04920 | Adipocytokine signaling pathway | 1.78E-10 | 8.79E-10 | 7124/8517/4790/3551/1147/7186/7132/4792/8717/7133 | 10 |
| hsa05160 | Hepatitis C | 3.00E-10 | 1.40E-09 | 7124/8737/7189/8517/4790/3551/1147/7186/7132/4792/8717/7098/841 | 13 |
| hsa05140 | Leishmaniasis | 5.46E-10 | 2.44E-09 | 7124/7099/7189/6885/23118/10454/4790/4792/4843/5743 | 10 |
| hsa05164 | Influenza A | 9.49E-10 | 4.04E-09 | 7124/7099/8517/4790/3551/1147/7132/4792/8717/7098/841/5340/6352 | 13 |
| hsa05167 | Kaposi sarcoma-associated herpesvirus infection | 2.88E-09 | 1.17E-08 | 8517/4790/3551/1147/7186/7132/4792/8717/7098/841/5608/1234/5743 | 13 |
| hsa05222 | Small cell lung cancer | 3.26E-09 | 1.27E-08 | 7189/8517/330/4790/3551/1147/7186/4792/4843/5743 | 10 |
| hsa01523 | Antifolate resistance | 4.42E-09 | 1.66E-08 | 7124/8517/4790/3551/1147/7298/1719 | 7 |
| hsa05162 | Measles | 1.38E-08 | 4.98E-08 | 7099/7189/8517/6885/23118/4790/3551/1147/4792/8717/841 | 11 |
| hsa05418 | Fluid shear stress and atherosclerosis | 1.49E-08 | 5.17E-08 | 7124/8517/6885/4790/3551/1147/7132/5608/7056/5327/6401 | 11 |
| hsa05168 | Herpes simplex virus 1 infection | 3.15E-07 | 1.05E-06 | 7124/7189/8517/330/6885/23118/10454/4790/3551/1147/7186/7132/4792/8717/7098/841/6352 | 17 |
| hsa05235 | PD-L1 expression and PD-1 checkpoint pathway in cancer | 6.09E-07 | 1.97E-06 | 7099/7189/8517/4790/3551/1147/4792/5608 | 8 |
| hsa04010 | MAPK signaling pathway | 6.81E-07 | 2.13E-06 | 7124/7189/8517/6885/23118/10454/4790/3551/1147/7186/7132/8717/5608 | 13 |
| hsa04217 | Necroptosis | 7.42E-07 | 2.21E-06 | 7124/7099/8737/330/7186/7132/8717/7098/841/142 | 10 |
| hsa04623 | Cytosolic DNA-sensing pathway | 7.55E-07 | 2.21E-06 | 8737/8517/4790/3551/1147/4792/6352 | 7 |
| hsa04625 | C-type lectin receptor signaling pathway | 2.01E-06 | 5.72E-06 | 7124/8517/4790/3551/1147/4792/841/5743 | 8 |
| hsa05215 | Prostate cancer | 1.41E-05 | 3.87E-05 | 8517/4790/3551/1147/4792/5328/5327 | 7 |
| hsa04061 | Viral protein interaction with cytokine and cytokine receptor | 1.72E-05 | 4.47E-05 | 7124/7132/7133/6352/7852/6387/1234 | 7 |
| hsa04933 | AGE-RAGE signaling pathway in diabetic complications | 1.72E-05 | 4.47E-05 | 7124/4790/5054/2152/7056/183/6401 | 7 |
| hsa04660 | T cell receptor signaling pathway | 2.22E-05 | 5.56E-05 | 7124/8517/6885/4790/3551/1147/4792 | 7 |
| hsa05120 | Epithelial cell signaling in Helicobacter pylori infection | 2.25E-05 | 5.56E-05 | 8517/4790/3551/1147/4792/6352 | 6 |
| hsa04062 | Chemokine signaling pathway | 2.31E-05 | 5.56E-05 | 8517/4790/3551/1147/4792/6352/7852/6387/1234 | 9 |
| hsa05133 | Pertussis | 3.61E-05 | 8.46E-05 | 7124/7099/7189/4790/23643/4843 | 6 |
| hsa05165 | Human papillomavirus infection | 7.42E-05 | 0.00017 | 7124/8517/4790/3551/1147/7132/8717/7098/841/7448/5743 | 11 |
| hsa05134 | Legionellosis | 0.000102 | 0.000227 | 7124/7099/4790/4792/841 | 5 |
| hsa05152 | Tuberculosis | 0.000111 | 0.000242 | 7124/7099/7189/4790/7132/8717/841/4843 | 8 |
| hsa04659 | Th17 cell differentiation | 0.000244 | 0.00052 | 8517/4790/3551/1147/4792/196 | 6 |
| hsa04931 | Insulin resistance | 0.000257 | 0.000535 | 7124/4790/3551/7132/4792/183 | 6 |
| hsa05220 | Chronic myeloid leukemia | 0.000397 | 0.00081 | 8517/4790/3551/1147/4792 | 5 |
| hsa04662 | B cell receptor signaling pathway | 0.000565 | 0.001126 | 8517/4790/3551/1147/4792 | 5 |
| hsa05144 | Malaria | 0.00075 | 0.001465 | 7124/7099/6401/6403 | 4 |
| hsa05014 | Amyotrophic lateral sclerosis (ALS) | 0.000809 | 0.001547 | 7124/7132/5608/7133 | 4 |
| hsa04614 | Renin-angiotensin system | 0.000861 | 0.001613 | 183/5972/1636 | 3 |
| hsa04658 | Th1 and Th2 cell differentiation | 0.000955 | 0.001755 | 8517/4790/3551/1147/4792 | 5 |
| hsa05323 | Rheumatoid arthritis | 0.001003 | 0.001808 | 7124/7099/6352/6387/4312 | 5 |
| hsa05203 | Viral carcinogenesis | 0.001314 | 0.002322 | 8517/4790/7186/4792/8717/841/1234 | 7 |
| hsa04932 | Non-alcoholic fatty liver disease (NAFLD) | 0.001415 | 0.002454 | 7124/4790/3551/7186/7132/841 | 6 |
| hsa05166 | Human T-cell leukemia virus 1 infection | 0.002149 | 0.00366 | 7124/8517/4790/3551/1147/7132/4792 | 7 |
| hsa05221 | Acute myeloid leukemia | 0.002247 | 0.003757 | 8517/4790/3551/1147 | 4 |
| hsa04215 | Apoptosis - multiple species | 0.002286 | 0.003757 | 330/7132/841 | 3 |
| hsa04071 | Sphingolipid signaling pathway | 0.002988 | 0.004827 | 7124/4790/7186/7132/8717 | 5 |
| hsa05212 | Pancreatic cancer | 0.003558 | 0.005572 | 8517/4790/3551/1147 | 4 |
| hsa04611 | Platelet activation | 0.003569 | 0.005572 | 2147/2243/2266/2244/6915 | 5 |
| hsa05204 | Chemical carcinogenesis | 0.004674 | 0.007178 | 5743/1576/1557/3290 | 4 |
| hsa05150 | Staphylococcus aureus infection | 0.008142 | 0.012303 | 5340/2266/6403/6404 | 4 |
| hsa05146 | Amoebiasis | 0.010038 | 0.014927 | 7124/7099/4790/4843 | 4 |
| hsa04060 | Cytokine-cytokine receptor interaction | 0.010631 | 0.015562 | 7124/7132/7133/6352/7852/6387/1234 | 7 |
| hsa00670 | One carbon pool by folate | 0.011724 | 0.016898 | 7298/1719 | 2 |
| hsa04066 | HIF-1 signaling pathway | 0.012588 | 0.017868 | 7099/4790/5054/4843 | 4 |
| hsa00900 | Terpenoid backbone biosynthesis | 0.014103 | 0.01972 | 3157/3156 | 2 |
| hsa05321 | Inflammatory bowel disease (IBD) | 0.016578 | 0.022839 | 7124/7099/4790 | 3 |
| hsa04722 | Neurotrophin signaling pathway | 0.016897 | 0.022942 | 7189/4790/3551/4792 | 4 |
| hsa04924 | Renin secretion | 0.019425 | 0.025998 | 183/5972/1636 | 3 |
| hsa04926 | Relaxin signaling pathway | 0.022036 | 0.029076 | 4790/4792/4843/4312 | 4 |
| hsa00591 | Linoleic acid metabolism | 0.023882 | 0.031075 | 1576/1557 | 2 |
| hsa03320 | PPAR signaling pathway | 0.025021 | 0.032111 | 7316/3157/4312 | 3 |
| hsa05205 | Proteoglycans in cancer | 0.0268 | 0.033929 | 7124/7099/5328/5329/7448 | 5 |
| hsa05132 | Salmonella infection | 0.028569 | 0.035687 | 7099/4790/4843 | 3 |
| hsa05143 | African trypanosomiasis | 0.037554 | 0.046168 | 7124/6401 | 2 |
| hsa04150 | mTOR signaling pathway | 0.037946 | 0.046168 | 7124/3551/1147/7132 | 4 |
| hsa05410 | Hypertrophic cardiomyopathy (HCM) | 0.038547 | 0.046298 | 7124/183/1636 | 3 |
| hsa04930 | Type II diabetes mellitus | 0.055663 | 0.066009 | 7124/3551 | 2 |
| hsa04672 | Intestinal immune network for IgA production | 0.06226 | 0.072909 | 7852/6387 | 2 |
| hsa05202 | Transcriptional misregulation in cancer | 0.068341 | 0.079043 | 330/4790/5328/5327 | 4 |
| hsa04151 | PI3K-Akt signaling pathway | 0.072639 | 0.082989 | 7099/8517/4790/3551/1147/7448 | 6 |
| hsa00072 | Synthesis and degradation of ketone bodies | 0.080068 | 0.090374 | 3157 | 1 |
| hsa00240 | Pyrimidine metabolism | 0.081039 | 0.090382 | 1854/7298 | 2 |
| hsa00130 | Ubiquinone and other terpenoid-quinone biosynthesis | 0.087718 | 0.096389 | 6898 | 1 |
| hsa00140 | Steroid hormone biosynthesis | 0.088483 | 0.096389 | 1576/3290 | 2 |
| hsa00590 | Arachidonic acid metabolism | 0.096122 | 0.103507 | 5743/1557 | 2 |
| hsa04371 | Apelin signaling pathway | 0.10501 | 0.111793 | 5054/5327/4843 | 3 |
| hsa04664 | Fc epsilon RI signaling pathway | 0.109253 | 0.115003 | 7124/5608 | 2 |
| hsa00982 | Drug metabolism - cytochrome P450 | 0.120081 | 0.122279 | 1576/1557 | 2 |
| hsa04115 | p53 signaling pathway | 0.120081 | 0.122279 | 841/5054 | 2 |
| hsa04976 | Bile secretion | 0.120081 | 0.122279 | 1576/3156 | 2 |
| hsa04514 | Cell adhesion molecules (CAMs) | 0.122749 | 0.122416 | 6401/6403/6404 | 3 |
| hsa01524 | Platinum drug resistance | 0.122829 | 0.122416 | 330/841 | 2 |
| hsa04014 | Ras signaling pathway | 0.126361 | 0.124611 | 8517/4790/3551/1147 | 4 |
| hsa00980 | Metabolism of xenobiotics by cytochrome P450 | 0.131164 | 0.1278 | 1576/3290 | 2 |
| hsa00360 | Phenylalanine metabolism | 0.132324 | 0.1278 | 6898 | 1 |
| hsa00983 | Drug metabolism - other enzymes | 0.139627 | 0.133478 | 1576/1854 | 2 |
| hsa04218 | Cellular senescence | 0.147318 | 0.139408 | 4790/5054/5608 | 3 |
| hsa04080 | Neuroactive ligand-receptor interaction | 0.151178 | 0.14163 | 2147/5340/183/2908/6915 | 5 |
| hsa00220 | Arginine biosynthesis | 0.16086 | 0.149208 | 4843 | 1 |
| hsa05010 | Alzheimer disease | 0.169264 | 0.155464 | 7124/7132/841 | 3 |
| hsa05414 | Dilated cardiomyopathy (DCM) | 0.189492 | 0.172353 | 7124/183 | 2 |
| hsa00790 | Folate biosynthesis | 0.195233 | 0.175868 | 1719 | 1 |
| hsa00650 | Butanoate metabolism | 0.208591 | 0.186111 | 3157 | 1 |
| hsa04020 | Calcium signaling pathway | 0.215689 | 0.190629 | 7852/4843/6915 | 3 |
| hsa05310 | Asthma | 0.228219 | 0.199818 | 7124 | 1 |
| hsa04670 | Leukocyte transendothelial migration | 0.238293 | 0.206707 | 7852/6387 | 2 |
| hsa03410 | Base excision repair | 0.241038 | 0.207169 | 142 | 1 |
| hsa04726 | Serotonergic synapse | 0.247544 | 0.210827 | 5743/1557 | 2 |
| hsa05020 | Prion diseases | 0.253646 | 0.213658 | 6352 | 1 |
| hsa05206 | MicroRNAs in cancer | 0.256231 | 0.213658 | 4790/3551/5328/5743 | 4 |
| hsa00350 | Tyrosine metabolism | 0.259873 | 0.213658 | 6898 | 1 |
| hsa04810 | Regulation of actin cytoskeleton | 0.259991 | 0.213658 | 2147/7852/6387 | 3 |
| hsa04152 | AMPK signaling pathway | 0.262991 | 0.214244 | 6885/3156 | 2 |
| hsa05330 | Allograft rejection | 0.272173 | 0.217934 | 7124 | 1 |
| hsa05340 | Primary immunodeficiency | 0.272173 | 0.217934 | 8517 | 1 |
| hsa05219 | Bladder cancer | 0.290247 | 0.2285 | 4312 | 1 |
| hsa05332 | Graft-versus-host disease | 0.290247 | 0.2285 | 7124 | 1 |
| hsa04068 | FoxO signaling pathway | 0.29698 | 0.231853 | 3551/1147 | 2 |
| hsa04940 | Type I diabetes mellitus | 0.30205 | 0.233862 | 7124 | 1 |
| hsa04120 | Ubiquitin mediated proteolysis | 0.312376 | 0.239874 | 7189/330 | 2 |
| hsa04140 | Autophagy - animal | 0.315448 | 0.240264 | 7189/6885 | 2 |
| hsa00280 | Valine, leucine and isoleucine degradation | 0.330718 | 0.248087 | 3157 | 1 |
| hsa00270 | Cysteine and methionine metabolism | 0.336311 | 0.248087 | 6898 | 1 |
| hsa04913 | Ovarian steroidogenesis | 0.336311 | 0.248087 | 5743 | 1 |
| hsa05030 | Cocaine addiction | 0.336311 | 0.248087 | 4790 | 1 |
| hsa00330 | Arginine and proline metabolism | 0.341858 | 0.24844 | 4843 | 1 |
| hsa04144 | Endocytosis | 0.342094 | 0.24844 | 7189/7852/1234 | 3 |
| hsa04072 | Phospholipase D signaling pathway | 0.349023 | 0.251523 | 2147/183 | 2 |
| hsa04923 | Regulation of lipolysis in adipocytes | 0.368915 | 0.262687 | 5743 | 1 |
| hsa04934 | Cushing syndrome | 0.370123 | 0.262687 | 183/196 | 2 |
| hsa04370 | VEGF signaling pathway | 0.389769 | 0.27455 | 5743 | 1 |
| hsa05416 | Viral myocarditis | 0.394875 | 0.276071 | 841 | 1 |
| hsa04927 | Cortisol synthesis and secretion | 0.419783 | 0.291312 | 183 | 1 |
| hsa00830 | Retinol metabolism | 0.429461 | 0.295836 | 1576 | 1 |
| hsa04917 | Prolactin signaling pathway | 0.443681 | 0.302177 | 4790 | 1 |
| hsa04360 | Axon guidance | 0.445927 | 0.302177 | 7852/6387 | 2 |
| hsa04520 | Adherens junction | 0.448343 | 0.302177 | 6885 | 1 |
| hsa01230 | Biosynthesis of amino acids | 0.466609 | 0.312242 | 6898 | 1 |
| hsa04612 | Antigen processing and presentation | 0.479916 | 0.318869 | 7124 | 1 |
| hsa04510 | Focal adhesion | 0.49545 | 0.326872 | 330/7448 | 2 |
| hsa04146 | Peroxisome | 0.501372 | 0.328466 | 4843 | 1 |
| hsa04512 | ECM-receptor interaction | 0.521955 | 0.339576 | 7448 | 1 |
| hsa04211 | Longevity regulating pathway | 0.525971 | 0.339829 | 4790 | 1 |
| hsa04024 | cAMP signaling pathway | 0.539642 | 0.345231 | 4790/4792 | 2 |
| hsa04912 | GnRH signaling pathway | 0.541702 | 0.345231 | 5608 | 1 |
| hsa04350 | TGF-beta signaling pathway | 0.545553 | 0.345336 | 7124 | 1 |
| hsa04925 | Aldosterone synthesis and secretion | 0.560644 | 0.352404 | 183 | 1 |
| hsa04640 | Hematopoietic cell lineage | 0.564339 | 0.352404 | 7124 | 1 |
| hsa04750 | Inflammatory mediator regulation of TRP channels | 0.568003 | 0.352404 | 5608 | 1 |
| hsa04919 | Thyroid hormone signaling pathway | 0.63213 | 0.387063 | 10499 | 1 |
| hsa04935 | Growth hormone synthesis, secretion and action | 0.63213 | 0.387063 | 5608 | 1 |
| hsa04650 | Natural killer cell mediated cytotoxicity | 0.667699 | 0.404336 | 7124 | 1 |
| hsa04270 | Vascular smooth muscle contraction | 0.670505 | 0.404336 | 183 | 1 |
| hsa05322 | Systemic lupus erythematosus | 0.673288 | 0.404336 | 7124 | 1 |
| hsa04910 | Insulin signaling pathway | 0.684191 | 0.407265 | 3551 | 1 |
| hsa04915 | Estrogen signaling pathway | 0.68686 | 0.407265 | 10499 | 1 |
| hsa04723 | Retrograde endocannabinoid signaling | 0.712359 | 0.417651 | 5743 | 1 |
| hsa04261 | Adrenergic signaling in cardiomyocytes | 0.714794 | 0.417651 | 183 | 1 |
| hsa04145 | Phagosome | 0.721976 | 0.417651 | 7099 | 1 |
| hsa04921 | Oxytocin signaling pathway | 0.72433 | 0.417651 | 5743 | 1 |
| hsa04390 | Hippo signaling pathway | 0.726665 | 0.417651 | 5054 | 1 |
| hsa04310 | Wnt signaling pathway | 0.74027 | 0.422875 | 6885 | 1 |
| hsa04141 | Protein processing in endoplasmic reticulum | 0.753207 | 0.427658 | 7186 | 1 |
| hsa05016 | Huntington disease | 0.803996 | 0.453745 | 841 | 1 |
| hsa04015 | Rap1 signaling pathway | 0.830535 | 0.465916 | 5608 | 1 |
