# Supplementary material for: Investigating the multi-target pharmacological mechanism of danhong injection acting on unstable angina by combined network pharmacology and molecular docking
Source: BMC Complement Med Ther. 2020 Mar 2;20:66. doi: 10.1186/s12906-020-2853-5 (PMC7076845; doi:10.1186/s12906-020-2853-5)
Supplement: Supplementary file 5 — Additional file 5 Supplementary 5. Table S5. The information of KEGG pathway enrichment analysis for each cluster. [file 12906_2020_2853_MOESM5_ESM.docx]

Table S5. The information of KEGG pathway enrichment analysis for each cluster

Module 1:

| ID | Description | pvalue | qvalue | geneID | Count |
| --- | --- | --- | --- | --- | --- |
| hsa04064 | NF-kappa B signaling pathway | 2.68E-30 | 9.61E-29 | 7132/8517/10454/8737/8717/7186/7124/23118/6885/7099/3551/1147/7189/330/4792/4790 | 16 |
| hsa04668 | TNF signaling pathway | 3.48E-24 | 6.22E-23 | 7132/8517/10454/8737/8717/7186/7124/23118/6885/3551/1147/330/4792/4790 | 14 |
| hsa05130 | Pathogenic Escherichia coli infection | 9.26E-23 | 1.10E-21 | 7132/8517/10454/8737/8717/7186/7124/23118/6885/7099/3551/1147/7189/4792/4790 | 15 |
| hsa05170 | Human immunodeficiency virus 1 infection | 1.96E-22 | 1.75E-21 | 7132/8517/10454/8737/8717/7186/7124/23118/6885/7099/3551/1147/7189/4792/4790 | 15 |
| hsa05145 | Toxoplasmosis | 9.64E-22 | 6.11E-21 | 7132/8517/10454/7124/23118/6885/7099/3551/1147/7189/330/4792/4790 | 13 |
| hsa05131 | Shigellosis | 1.02E-21 | 6.11E-21 | 7132/8517/10454/8737/8717/7186/7124/23118/6885/7099/3551/1147/7189/4792/4790 | 15 |
| hsa04621 | NOD-like receptor signaling pathway | 3.92E-21 | 2.00E-20 | 8517/10454/8737/7186/7124/23118/6885/7099/3551/1147/7189/330/4792/4790 | 14 |
| hsa04620 | Toll-like receptor signaling pathway | 7.73E-20 | 3.46E-19 | 8517/10454/8737/7124/23118/6885/7099/3551/1147/7189/4792/4790 | 12 |
| hsa04622 | RIG-I-like receptor signaling pathway | 1.30E-19 | 5.15E-19 | 8517/8737/8717/7186/7124/6885/3551/1147/7189/4792/4790 | 11 |
| hsa05135 | Yersinia infection | 4.67E-19 | 1.67E-18 | 8517/10454/7186/7124/23118/6885/7099/3551/1147/7189/4792/4790 | 12 |
| hsa04380 | Osteoclast differentiation | 1.05E-18 | 3.40E-18 | 7132/8517/10454/7186/7124/23118/6885/3551/1147/7189/4792/4790 | 12 |
| hsa05169 | Epstein-Barr virus infection | 2.57E-18 | 7.66E-18 | 8517/10454/8737/8717/7186/7124/23118/6885/3551/1147/7189/4792/4790 | 13 |
| hsa04657 | IL-17 signaling pathway | 4.07E-18 | 1.12E-17 | 8517/8717/7186/7124/23118/6885/3551/1147/7189/4792/4790 | 11 |
| hsa04210 | Apoptosis | 2.78E-16 | 7.12E-16 | 7132/8517/8737/8717/7186/7124/3551/1147/330/4792/4790 | 11 |
| hsa05160 | Hepatitis C | 1.22E-15 | 2.91E-15 | 7132/8517/8737/8717/7186/7124/3551/1147/7189/4792/4790 | 11 |
| hsa05161 | Hepatitis B | 2.00E-15 | 4.48E-15 | 8517/10454/7124/23118/6885/7099/3551/1147/7189/4792/4790 | 11 |
| hsa04920 | Adipocytokine signaling pathway | 3.77E-15 | 7.93E-15 | 7132/8517/8717/7186/7124/3551/1147/4792/4790 | 9 |
| hsa05168 | Herpes simplex virus 1 infection | 5.68E-15 | 1.13E-14 | 7132/8517/10454/8717/7186/7124/23118/6885/3551/1147/7189/330/4792/4790 | 14 |
| hsa04010 | MAPK signaling pathway | 2.88E-14 | 5.43E-14 | 7132/8517/10454/8717/7186/7124/23118/6885/3551/1147/7189/4790 | 12 |
| hsa05162 | Measles | 3.15E-14 | 5.64E-14 | 8517/8717/23118/6885/7099/3551/1147/7189/4792/4790 | 10 |
| hsa05142 | Chagas disease (American trypanosomiasis) | 1.48E-13 | 2.52E-13 | 7132/8517/7124/7099/3551/1147/7189/4792/4790 | 9 |
| hsa05140 | Leishmaniasis | 1.21E-12 | 1.98E-12 | 10454/7124/23118/6885/7099/7189/4792/4790 | 8 |
| hsa05163 | Human cytomegalovirus infection | 4.44E-12 | 6.92E-12 | 7132/8517/8737/8717/7186/7124/3551/1147/4792/4790 | 10 |
| hsa05222 | Small cell lung cancer | 5.29E-12 | 7.89E-12 | 8517/7186/3551/1147/7189/330/4792/4790 | 8 |
| hsa05164 | Influenza A | 1.60E-11 | 2.28E-11 | 7132/8517/8717/7124/7099/3551/1147/4792/4790 | 9 |
| hsa05235 | PD-L1 expression and PD-1 checkpoint pathway in cancer | 3.09E-10 | 4.26E-10 | 8517/7099/3551/1147/7189/4792/4790 | 7 |
| hsa04660 | T cell receptor signaling pathway | 9.37E-10 | 1.24E-09 | 8517/7124/6885/3551/1147/4792/4790 | 7 |
| hsa05167 | Kaposi sarcoma-associated herpesvirus infection | 1.58E-09 | 2.01E-09 | 7132/8517/8717/7186/3551/1147/4792/4790 | 8 |
| hsa04623 | Cytosolic DNA-sensing pathway | 2.25E-09 | 2.78E-09 | 8517/8737/3551/1147/4792/4790 | 6 |
| hsa01523 | Antifolate resistance | 3.86E-09 | 4.60E-09 | 8517/7124/3551/1147/4790 | 5 |
| hsa05418 | Fluid shear stress and atherosclerosis | 7.24E-09 | 8.36E-09 | 7132/8517/7124/6885/3551/1147/4790 | 7 |
| hsa04217 | Necroptosis | 2.11E-08 | 2.36E-08 | 7132/8737/8717/7186/7124/7099/330 | 7 |
| hsa04625 | C-type lectin receptor signaling pathway | 4.79E-08 | 5.19E-08 | 8517/7124/3551/1147/4792/4790 | 6 |
| hsa05166 | Human T-cell leukemia virus 1 infection | 1.69E-07 | 1.78E-07 | 7132/8517/7124/3551/1147/4792/4790 | 7 |
| hsa05120 | Epithelial cell signaling in Helicobacter pylori infection | 2.62E-07 | 2.68E-07 | 8517/3551/1147/4792/4790 | 5 |
| hsa05220 | Chronic myeloid leukemia | 3.96E-07 | 3.94E-07 | 8517/3551/1147/4792/4790 | 5 |
| hsa04662 | B cell receptor signaling pathway | 5.81E-07 | 5.62E-07 | 8517/3551/1147/4792/4790 | 5 |
| hsa04658 | Th1 and Th2 cell differentiation | 1.03E-06 | 9.74E-07 | 8517/3551/1147/4792/4790 | 5 |
| hsa05152 | Tuberculosis | 1.25E-06 | 1.15E-06 | 7132/8717/7124/7099/7189/4790 | 6 |
| hsa05215 | Prostate cancer | 1.35E-06 | 1.20E-06 | 8517/3551/1147/4792/4790 | 5 |
| hsa04659 | Th17 cell differentiation | 2.19E-06 | 1.91E-06 | 8517/3551/1147/4792/4790 | 5 |
| hsa04931 | Insulin resistance | 2.30E-06 | 1.96E-06 | 7132/7124/3551/4792/4790 | 5 |
| hsa05165 | Human papillomavirus infection | 2.71E-06 | 2.26E-06 | 7132/8517/8717/7124/3551/1147/4790 | 7 |
| hsa04071 | Sphingolipid signaling pathway | 3.71E-06 | 3.02E-06 | 7132/8717/7186/7124/4790 | 5 |
| hsa05134 | Legionellosis | 5.28E-06 | 4.20E-06 | 7124/7099/4792/4790 | 4 |
| hsa05221 | Acute myeloid leukemia | 1.01E-05 | 7.87E-06 | 8517/3551/1147/4790 | 4 |
| hsa04932 | Non-alcoholic fatty liver disease (NAFLD) | 1.12E-05 | 8.52E-06 | 7132/7186/7124/3551/4790 | 5 |
| hsa05133 | Pertussis | 1.67E-05 | 1.22E-05 | 7124/7099/7189/4790 | 4 |
| hsa05212 | Pancreatic cancer | 1.67E-05 | 1.22E-05 | 8517/3551/1147/4790 | 4 |
| hsa04062 | Chemokine signaling pathway | 3.54E-05 | 2.53E-05 | 8517/3551/1147/4792/4790 | 5 |
| hsa05203 | Viral carcinogenesis | 4.76E-05 | 3.34E-05 | 8517/8717/7186/4792/4790 | 5 |
| hsa04722 | Neurotrophin signaling pathway | 9.79E-05 | 6.74E-05 | 3551/7189/4792/4790 | 4 |
| hsa04150 | mTOR signaling pathway | 0.000259 | 0.000175 | 7132/7124/3551/1147 | 4 |
| hsa05321 | Inflammatory bowel disease (IBD) | 0.000327 | 0.000217 | 7124/7099/4790 | 3 |
| hsa04151 | PI3K-Akt signaling pathway | 0.000677 | 0.000441 | 8517/7099/3551/1147/4790 | 5 |
| hsa05146 | Amoebiasis | 0.001225 | 0.000783 | 7124/7099/4790 | 3 |
| hsa04014 | Ras signaling pathway | 0.001249 | 0.000784 | 8517/3551/1147/4790 | 4 |
| hsa04215 | Apoptosis - multiple species | 0.002058 | 0.00127 | 7132/330 | 2 |
| hsa04930 | Type II diabetes mellitus | 0.004219 | 0.002559 | 7124/3551 | 2 |
| hsa05144 | Malaria | 0.004969 | 0.002964 | 7124/7099 | 2 |
| hsa05014 | Amyotrophic lateral sclerosis (ALS) | 0.005165 | 0.00303 | 7132/7124 | 2 |
| hsa05132 | Salmonella infection | 0.012344 | 0.007126 | 7099/4790 | 2 |
| hsa05323 | Rheumatoid arthritis | 0.016441 | 0.00934 | 7124/7099 | 2 |
| hsa04061 | Viral protein interaction with cytokine and cytokine receptor | 0.018858 | 0.010383 | 7132/7124 | 2 |
| hsa04933 | AGE-RAGE signaling pathway in diabetic complications | 0.018858 | 0.010383 | 7124/4790 | 2 |
| hsa04066 | HIF-1 signaling pathway | 0.022173 | 0.012024 | 7099/4790 | 2 |
| hsa04926 | Relaxin signaling pathway | 0.030333 | 0.016203 | 4792/4790 | 2 |
| hsa04068 | FoxO signaling pathway | 0.031207 | 0.016425 | 3551/1147 | 2 |
| hsa04120 | Ubiquitin mediated proteolysis | 0.033434 | 0.017326 | 7189/330 | 2 |
| hsa04140 | Autophagy - animal | 0.033887 | 0.017326 | 6885/7189 | 2 |
| hsa05010 | Alzheimer disease | 0.050678 | 0.025546 | 7132/7124 | 2 |
| hsa05202 | Transcriptional misregulation in cancer | 0.058879 | 0.029267 | 330/4790 | 2 |
| hsa05310 | Asthma | 0.064355 | 0.031551 | 7124 | 1 |
| hsa05205 | Proteoglycans in cancer | 0.069293 | 0.033513 | 7124/7099 | 2 |
| hsa05143 | African trypanosomiasis | 0.076352 | 0.035944 | 7124 | 1 |
| hsa04024 | cAMP signaling pathway | 0.076558 | 0.035944 | 4792/4790 | 2 |
| hsa05330 | Allograft rejection | 0.078337 | 0.035944 | 7124 | 1 |
| hsa05340 | Primary immunodeficiency | 0.078337 | 0.035944 | 8517 | 1 |
| hsa05332 | Graft-versus-host disease | 0.084269 | 0.038177 | 7124 | 1 |
| hsa04940 | Type I diabetes mellitus | 0.088204 | 0.03946 | 7124 | 1 |
| hsa05030 | Cocaine addiction | 0.099913 | 0.044146 | 4790 | 1 |
| hsa04060 | Cytokine-cytokine receptor interaction | 0.128961 | 0.056286 | 7132/7124 | 2 |
| hsa04664 | Fc epsilon RI signaling pathway | 0.136064 | 0.05867 | 7124 | 1 |
| hsa04917 | Prolactin signaling pathway | 0.139788 | 0.058947 | 4790 | 1 |
| hsa05206 | MicroRNAs in cancer | 0.140609 | 0.058947 | 3551/4790 | 2 |
| hsa04520 | Adherens junction | 0.141645 | 0.058947 | 6885 | 1 |
| hsa01524 | Platinum drug resistance | 0.145347 | 0.059792 | 330 | 1 |
| hsa03320 | PPAR signaling pathway | 0.150872 | 0.06136 | 7316 | 1 |
| hsa04612 | Antigen processing and presentation | 0.154537 | 0.062144 | 7124 | 1 |
| hsa04211 | Longevity regulating pathway | 0.174428 | 0.069303 | 4790 | 1 |
| hsa05410 | Hypertrophic cardiomyopathy (HCM) | 0.176214 | 0.069303 | 7124 | 1 |
| hsa04350 | TGF-beta signaling pathway | 0.183323 | 0.071316 | 7124 | 1 |
| hsa05414 | Dilated cardiomyopathy (DCM) | 0.186856 | 0.071908 | 7124 | 1 |
| hsa04640 | Hematopoietic cell lineage | 0.192128 | 0.073151 | 7124 | 1 |
| hsa04152 | AMPK signaling pathway | 0.228142 | 0.085948 | 6885 | 1 |
| hsa04650 | Natural killer cell mediated cytotoxicity | 0.246397 | 0.091859 | 7124 | 1 |
| hsa05322 | Systemic lupus erythematosus | 0.249673 | 0.09212 | 7124 | 1 |
| hsa04910 | Insulin signaling pathway | 0.256183 | 0.093558 | 3551 | 1 |
| hsa04145 | Phagosome | 0.280127 | 0.101269 | 7099 | 1 |
| hsa04218 | Cellular senescence | 0.292598 | 0.103682 | 4790 | 1 |
| hsa04310 | Wnt signaling pathway | 0.292598 | 0.103682 | 6885 | 1 |
| hsa04141 | Protein processing in endoplasmic reticulum | 0.301818 | 0.105901 | 7186 | 1 |
| hsa04510 | Focal adhesion | 0.350538 | 0.121802 | 330 | 1 |
| hsa04144 | Endocytosis | 0.4183 | 0.143949 | 7189 | 1 |

Module 2:

| ID | Description | pvalue | qvalue | geneID | Count |
| --- | --- | --- | --- | --- | --- |
| hsa04061 | Viral protein interaction with cytokine and cytokine receptor | 1.17E-07 | 2.71E-06 | 6387/7852/1234/6352 | 4 |
| hsa04062 | Chemokine signaling pathway | 1.52E-06 | 1.76E-05 | 6387/7852/1234/6352 | 4 |
| hsa05163 | Human cytomegalovirus infection | 3.06E-06 | 2.36E-05 | 6387/7852/1234/6352 | 4 |
| hsa04060 | Cytokine-cytokine receptor interaction | 8.92E-06 | 5.16E-05 | 6387/7852/1234/6352 | 4 |
| hsa04672 | Intestinal immune network for IgA production | 0.000368 | 0.001705 | 6387/7852 | 2 |
| hsa05323 | Rheumatoid arthritis | 0.001324 | 0.005112 | 6387/6352 | 2 |
| hsa04670 | Leukocyte transendothelial migration | 0.001915 | 0.006336 | 6387/7852 | 2 |
| hsa04360 | Axon guidance | 0.004932 | 0.014277 | 6387/7852 | 2 |
| hsa05170 | Human immunodeficiency virus 1 infection | 0.006718 | 0.015702 | 7852/1234 | 2 |
| hsa04810 | Regulation of actin cytoskeleton | 0.00678 | 0.015702 | 6387/7852 | 2 |
| hsa04144 | Endocytosis | 0.009187 | 0.019342 | 7852/1234 | 2 |
| hsa04614 | Renin-angiotensin system | 0.014393 | 0.027775 | 183 | 1 |
| hsa05020 | Prion diseases | 0.021836 | 0.038898 | 6352 | 1 |
| hsa04623 | Cytosolic DNA-sensing pathway | 0.039029 | 0.058781 | 6352 | 1 |
| hsa04927 | Cortisol synthesis and secretion | 0.040247 | 0.058781 | 183 | 1 |
| hsa04924 | Renin secretion | 0.042681 | 0.058781 | 183 | 1 |
| hsa05120 | Epithelial cell signaling in Helicobacter pylori infection | 0.043289 | 0.058781 | 6352 | 1 |
| hsa05410 | Hypertrophic cardiomyopathy (HCM) | 0.055377 | 0.058781 | 183 | 1 |
| hsa05414 | Dilated cardiomyopathy (DCM) | 0.05898 | 0.058781 | 183 | 1 |
| hsa04925 | Aldosterone synthesis and secretion | 0.060179 | 0.058781 | 183 | 1 |
| hsa04933 | AGE-RAGE signaling pathway in diabetic complications | 0.061376 | 0.058781 | 183 | 1 |
| hsa04064 | NF-kappa B signaling pathway | 0.062572 | 0.058781 | 6387 | 1 |
| hsa05142 | Chagas disease (American trypanosomiasis) | 0.062572 | 0.058781 | 6352 | 1 |
| hsa04620 | Toll-like receptor signaling pathway | 0.063767 | 0.058781 | 6352 | 1 |
| hsa04931 | Insulin resistance | 0.066153 | 0.058781 | 183 | 1 |
| hsa04668 | TNF signaling pathway | 0.068534 | 0.058781 | 6352 | 1 |
| hsa05145 | Toxoplasmosis | 0.068534 | 0.058781 | 1234 | 1 |
| hsa04270 | Vascular smooth muscle contraction | 0.080366 | 0.066468 | 183 | 1 |
| hsa04072 | Phospholipase D signaling pathway | 0.089745 | 0.069728 | 183 | 1 |
| hsa04261 | Adrenergic signaling in cardiomyocytes | 0.090329 | 0.069728 | 183 | 1 |
| hsa04934 | Cushing syndrome | 0.093824 | 0.07009 | 183 | 1 |
| hsa05164 | Influenza A | 0.102516 | 0.07419 | 6352 | 1 |
| hsa04621 | NOD-like receptor signaling pathway | 0.108848 | 0.07609 | 6352 | 1 |
| hsa05167 | Kaposi sarcoma-associated herpesvirus infection | 0.111714 | 0.07609 | 1234 | 1 |
| hsa04020 | Calcium signaling pathway | 0.115714 | 0.076563 | 7852 | 1 |
| hsa05203 | Viral carcinogenesis | 0.120269 | 0.077366 | 1234 | 1 |
| hsa05131 | Shigellosis | 0.139972 | 0.087607 | 6352 | 1 |
| hsa04080 | Neuroactive ligand-receptor interaction | 0.196447 | 0.119719 | 183 | 1 |
| hsa05168 | Herpes simplex virus 1 infection | 0.273126 | 0.16218 | 6352 | 1 |

Module 3:

| ID | Description | pvalue | qvalue | geneID | Count |
| --- | --- | --- | --- | --- | --- |
| hsa04610 | Complement and coagulation cascades | 1.09E-15 | 3.22E-14 | 7448/5329/2147/3053/5340/5054/2159/5328 | 8 |
| hsa05205 | Proteoglycans in cancer | 0.001249 | 0.018412 | 7448/5329/5328 | 3 |
| hsa04080 | Neuroactive ligand-receptor interaction | 0.053854 | 0.235773 | 2147/5340 | 2 |
| hsa05014 | Amyotrophic lateral sclerosis (ALS) | 0.056332 | 0.235773 | 7133 | 1 |
| hsa04920 | Adipocytokine signaling pathway | 0.075529 | 0.235773 | 7133 | 1 |
| hsa04115 | p53 signaling pathway | 0.078695 | 0.235773 | 5054 | 1 |
| hsa04512 | ECM-receptor interaction | 0.095416 | 0.235773 | 7448 | 1 |
| hsa05150 | Staphylococcus aureus infection | 0.103674 | 0.235773 | 5340 | 1 |
| hsa05215 | Prostate cancer | 0.104702 | 0.235773 | 5328 | 1 |
| hsa04061 | Viral protein interaction with cytokine and cytokine receptor | 0.107779 | 0.235773 | 7133 | 1 |
| hsa04933 | AGE-RAGE signaling pathway in diabetic complications | 0.107779 | 0.235773 | 5054 | 1 |
| hsa04064 | NF-kappa B signaling pathway | 0.109825 | 0.235773 | 5328 | 1 |
| hsa05142 | Chagas disease (American trypanosomiasis) | 0.109825 | 0.235773 | 5054 | 1 |
| hsa04066 | HIF-1 signaling pathway | 0.116952 | 0.235773 | 5054 | 1 |
| hsa04668 | TNF signaling pathway | 0.119991 | 0.235773 | 7133 | 1 |
| hsa04611 | Platelet activation | 0.132055 | 0.24326 | 2147 | 1 |
| hsa04371 | Apelin signaling pathway | 0.144958 | 0.246035 | 5054 | 1 |
| hsa04072 | Phospholipase D signaling pathway | 0.155743 | 0.246035 | 2147 | 1 |
| hsa04390 | Hippo signaling pathway | 0.161574 | 0.246035 | 5054 | 1 |
| hsa04218 | Cellular senescence | 0.16737 | 0.246035 | 5054 | 1 |
| hsa05164 | Influenza A | 0.17695 | 0.246035 | 5340 | 1 |
| hsa05202 | Transcriptional misregulation in cancer | 0.192075 | 0.246035 | 5328 | 1 |
| hsa04510 | Focal adhesion | 0.204181 | 0.246035 | 7448 | 1 |
| hsa05130 | Pathogenic Escherichia coli infection | 0.206952 | 0.246035 | 2147 | 1 |
| hsa05170 | Human immunodeficiency virus 1 infection | 0.216126 | 0.246035 | 7133 | 1 |
| hsa04810 | Regulation of actin cytoskeleton | 0.217038 | 0.246035 | 2147 | 1 |
| hsa04060 | Cytokine-cytokine receptor interaction | 0.287866 | 0.314239 | 7133 | 1 |
| hsa05206 | MicroRNAs in cancer | 0.301162 | 0.317013 | 5328 | 1 |
| hsa05165 | Human papillomavirus infection | 0.317473 | 0.322658 | 7448 | 1 |
| hsa04151 | PI3K-Akt signaling pathway | 0.336598 | 0.330693 | 7448 | 1 |
